# Discovering novel clues of natural selection on four worldwide goat breeds

**DOI:** 10.1038/s41598-023-27490-x

**Published:** 2023-02-06

**Authors:** Arianna Manunza, Johanna Ramirez Diaz, Brian L Sayre, Paolo Cozzi, Tania Bobbo, Tatiana Deniskova, Arsen Dotsev, Natalia Zinovieva, Alessandra Stella

**Affiliations:** 1 Institute of Agricultural Biology and Biotechnology – Italian National Research Council – CNR, Via Edoardo Bassini 15, 20133 Milan, Italy; 2 Department of Biology, Virginia State University, PO Box 9064, Petersburg, VA 23806 USA; 3 L.K. Ernst Federal Research Center for Animal Husbandry, Dubrovitsy 60, Podolsk Municipal District, Moscow Region, 142132 Russia

**Keywords:** Evolution, Genetics

## Abstract

In goat breeds, the domestication followed by artificial selection for economically important traits have shaped genetic variation within populations, leading to the fixation of specific alleles for specific traits. This led to the formation and evolution of many different breeds specialised and raised for a particular purpose. However, and despite the intensity of artificial selection, natural selection continues acting, possibly leaving a more diluted contribution over time, whose traces may be more difficult to capture. In order to explore selection footprints as response of environmental adaptation, we analysed a total of 993 goats from four transboundary goats breeds (Angora, Boer, Nubian and Saanen) genotyped with the SNP chip 50 K using outlier detection, runs of homozygosity and haplotype-based detection methods. Our results showed that all methods identified footprints on chromosome 6 (from 30 to 49 Mb) for two specific populations of Nubian goats sampled in Egypt. In Angora and Saanen breeds, we detected two selective sweeps using HapFLK, on chromosome 21 (from 52 to 55 Mb) and chromosome 25 (from 1 to 5 Mb) respectively. The analysis of runs of homozygosity showed some hotspots in all breeds. The overall investigation of the selected regions detected combining the different approaches and the gene ontology exploration revealed both novel and well-known loci related to adaptation, especially for heat stress. Our findings can help to better understand the balance between the two selective pressures in commercial goat breeds providing new insights on the molecular mechanisms of adaptation.

## Introduction

*Capra hircus* is one of the most important worldwide farmed species and its domestication dates back to the early Neolithic era (ca. 11,000 YBP) in the Fertile Crescent^1^. Goats have been selected during centuries for different traits (milk, meat, wool or leather) show high resistance to stress and have a great ability to adapt to various agro-climatic conditions^2^. This quick adaptation implies several changes in physiology, morphology, behaviour, phenotype, and at the basis of all, in genetics. To analyse these genetic changes and the footprints that they left on the genome, the genome-wide scanning technology using SNP Arrays is a powerful and efficient tool widely used^3^ since several decades. For large transboundary breeds, genomic information has been used to identify candidate regions for traits of commercial interest and for application in breeding (genomic selection), investigating mainly the effect of artificial selection on the genome. In small local breed, genomic information has been exploited to investigate the ability to respond to environmental changes and challenges. This subdivision is generally based on the assumption that strong selection pressure applied to commercial breeds lead to negative impacts on their ability to adapt in comparison with traditional breeds, due to stronger connection to their original environments^4^. The role of the natural selection in shaping the genetic architecture of the highly selected, transboundary breeds has not yet been investigated. Little is known about how transboundary breeds have adapted to a wide range of different environments and management conditions. Indeed, natural selection continues acting, possibly leaving smaller but detectable contributions. To investigate this issue, it is important to first accurately choose an ideal model which go through strong anthropogenic selection over centuries and also to transboundary transport. Different methodologies that can be applied to detect selection signatures^5^, generally based on the comparison of statistics on genotypes at intra-populations versus inter-populations level. Two main categories of statistics have been developed at (1) intra-population (2) and inter-populations level. The first one is based on site frequency spectrum (SFS), linkage disequilibrium (LD) and reduced local variability. The second one focuses on single site differentiation and haplotype-based differentiation. Each of these approaches includes associated statistics and specific bioinformatic tools.

Objective of the study was to reveal breed-specific selection signatures linked to environmental variables and thus to identify loci potentially relevant for adaptation in commercial breeds. Our results will contribute to advancing knowledge on climate-driven adaptive evolution and to better understand the molecular mechanisms involved in this process. Moreover, results may find application in selective breeding and conservation management programs.

## Results

Publicly available genotypes of four commercial goat breeds were used to reveal breed-specific selection signature linked to environmental variable. Combining three different statistical methods, we detected several polymorphisms that revealed loci potentially affecting adaptation to agro-climatic conditions.

### Population relationships, clustering and outlier variant detection

**Figure 1:**
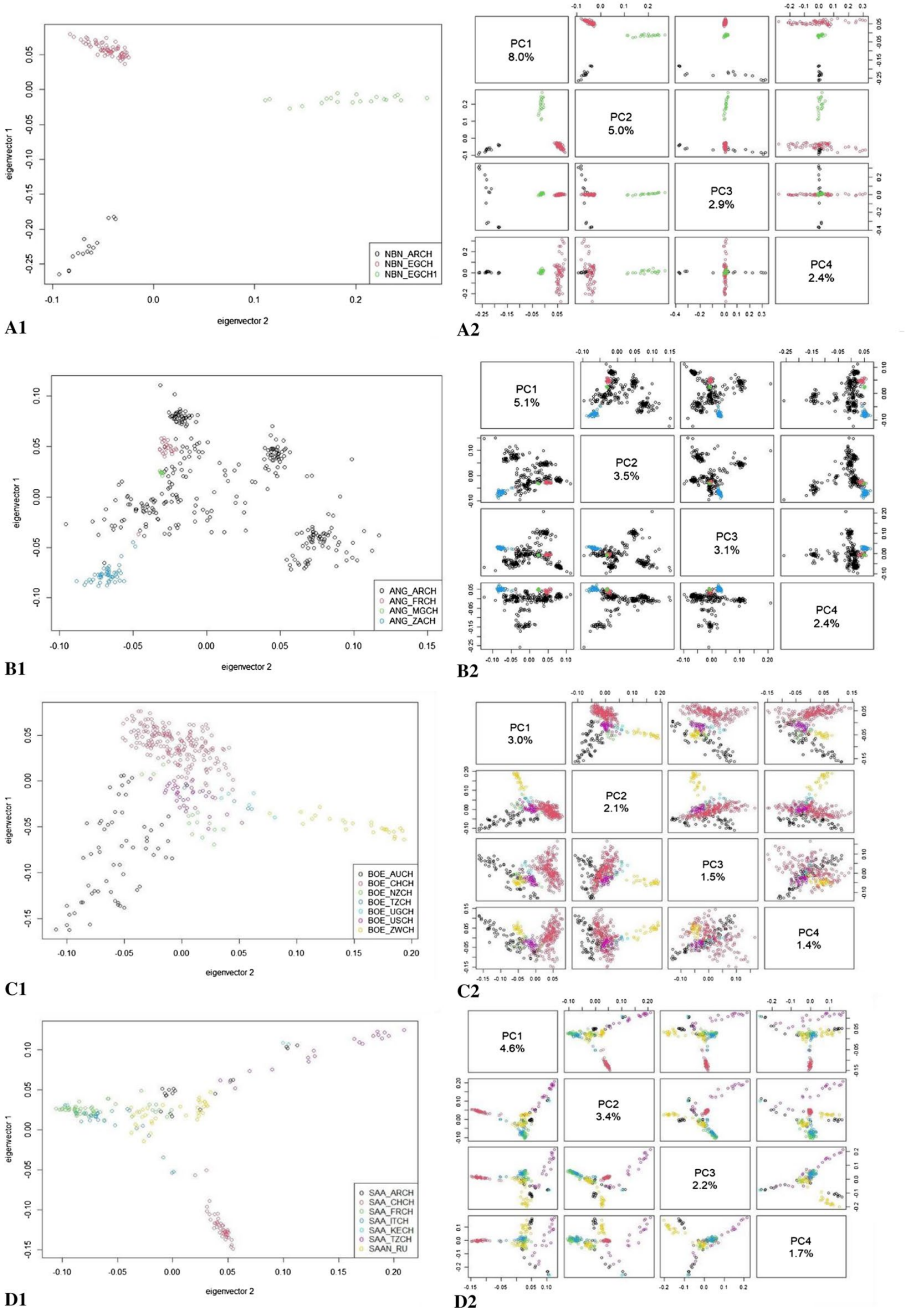
Distribution of samples in relation to their scores on the first and second principal components obtained after principal component analysis for the four breeds. Each point represents a single individual and for each breed a different colour was assigned. The legend that explains the correspondence between breeds and colour is in the lower right corner. (**A**) In the Nubian breed, after separation due to a subpopulation from Egypt, the percentage of variance explained up to 13% (eigenvector 1, X-axis, 8% and eigenvector 2, Y-axis, 5%). (**B**) In the Angora breed, the two components explained 8.6% of total variation (eigenvector 1, X-axis, 5.1% of variation and eigenvector 2, Y-axis, 3.5%). (**C**) In the Boer breed, the total percentage of variance is 5.1% (eigenvector 1, X-axis, 3% and eigenvector 2, Y-axis, 2.1%). (**D**) In the Saanen breed, the variance was a total of 8% (eigenvector 1, X-axis, 4.6% and eigenvector 2, Y-axis, 3.4%).

Figure 1 presents the PCA results where we can see that all populations clustered according to their geographic origin. The Nubian breed shows a clear separation not only between the two geographic areas (Argentina and Egypt) but also within the NBN_EGCH population (Fig. 1, A1) with the first two principal components that explain about 13% of genetic diversity (Fig. 1, A2). In Angora breed, all populations constitute well-defined clusters (Fig. 1, B1 and B2) except the Argentine population. Finally, in the Boer and Saanen breeds there are no well-defined clusters, even if it is possible to highlight a weak subdivision across them (Fig. 1, C1 and D1). The percentage of variance explained by the first two principal components indicates a low genetic diversity (Boer = 5% and Saanen = 8%, Fig. 1, C2 and D2).

**Figure 2:**
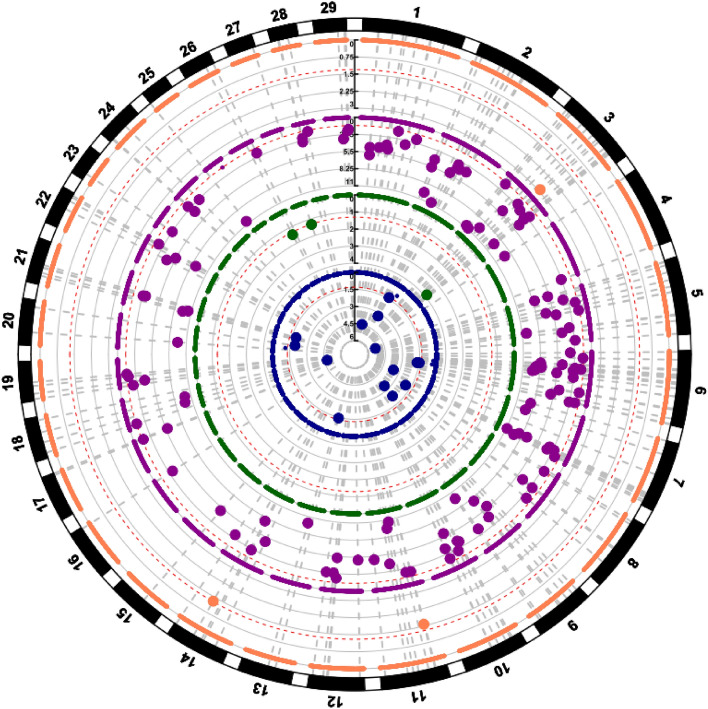
Circular Manhattan plot of outliers SNP detected with PCadapt analysis. One different colour is assigned to each breed: coral for Saanen, purple for Nubian, green for Boer and blue for Angora. The red dashed line indicates the threshold of significance of 0.05%. Every point is a SNP and with amplified the significant ones.

The second step of the analysis with PCAdapt identified several polymorphisms as outliers and putative signs of local adaptation. The number of outliers as well as the corresponding chromosomes and the genes falling into the putative genomic region under selection are summarised in Table 1 for all breeds. We found a total of twelve outliers in Angora, and three in both Saanen and Boer breeds remaining after Bonferroni’s correction (Table 1, Fig. 2).

**Table 1:** List of candidate gene retrieved inside the genomic regions included in the interval of 1 Mb on both sides of each outlier SNP detected by PCAdapt.

Breed	Chr	Position	Putative genes
Nubian	2	41557749	*ZDBF2, ADAM23, METTL21A, FASTKD2, CREB1, KLF7, CPO, MDH1B, DYTN, FAM237A, EEF1B2, NDUFS1, GPR1, NRP2, PARD3B*
	3	100143884 101377443	*RNF115, NUDT17, PIAS3, ANKRD35, ITGA10, RBM8A, LIX1L, ANKRD34A, POLR3GL, TXNIP, HJV, H2BC18, H2AC21, BOLA1, SV2A, MTMR11, OTUD7B, VPS45, PLEKHO1, ANP32E, CA14, APH1A, CIART, MRPS21, PRPF3, RPRD2, TARS2, ECM1, ADAMTSL4, MCL1, ENSA, HORMAD1, CTSS, CTSK, ARNT, SETDB1, CERS2, ANXA9, MINDY1, PRUNE1, BNIPL, CDC42SE1, MLLT11, GABPB2, SEMA6C, TNFAIP8L2, LYSMD1, SCNM1, TMOD4, VPS72, PIP5K1A, PSMD4, ZNF687, PI4KB, RFX5, SELENBP1, PSMB4, POGZ, TUFT1, SNX27, CELF3, RIIAD1, MRPL9, OAZ3, TDRKH, LINGO4, RORC, C2CD4D, THEM5, THEM4, TCHHL1*
	4	1257240	*PTPRN2, HSP40, UBE3C, MNX1, NOM1, LMBR1, RNF32*
	5	3006775 42494415 80765343 116979586	*CCDC91, PTHLH, REP15, PPFIBP1, TRHDE, KLHL42, CCDC91, PTPRB, CNOT2, ABCD2, CNOT2, CPNE8, KCNMB4, KIF21A, MYRFL, PTPRB, ATXN10, WNT7B, MIRLET7A, MIRLET7B, PPARA, CDPF1, PKDREJ, TTC38, TRMU, CELSR1, TBC1D22A*
	6	1951800 14358997 29025606 30831884 34602486 37401587 38473627 38696985 39295173 39985135 40745285 40875771 41727639 43160722 46214946 49627140 52110326 52236308	*BMPR1B, PDLIM5, HPGDS, SMARCAD1, ATOH1, GRID2, CCSER1, MMRN1, SNCA, GPRIN3, TIGD2, FAM13A, NAP1L5, PYURF, HERC5, HERC6, PPM1K, ABCG2, PKD2, SPP1, MEPE, IBSP, LAP3, MED28, FAM184B, DCAF16, NCAPG, LCORL, SLIT2, PACRGL, KCNIP4, ADGRA3, GBA3, PPARGC1A, DHX15, SOD3, CCDC149, LGI2, SEPSECS, PI4K2B, ZCCHC4, ANAPC4, SLC34A2, SEL1L3, RBPJ, CCKAR, TBC1D19, STIM2, SNORA70*
Angora	1	108667531	*RARRES1, LXN, VEPH1, PTX3, GFM1, SHOX2, IQCJ, MFSD1, MLF1*
	2	82677792	*KYNU, GTDC1*
	5	8209663	*PPP1R12A, SYT, NAV3, PAWR*
	6	71967098 71996981	*SRD5A3, KDR, NMU, THEM165, PDCL2, CLOCK, EXOC1L, CEO135, CRACD, AASDH, ARL9, THEGL, HOPX, NOA1, POLR2B*
	7	56050271	*KCTD16, NRC1, FGF1*
	9	5706994 14534276	*MEI4, IRAK1BP1, PHIP, LCA5, SH3BGRL2, CLVS2*
	13	29008963	*CDNF, HSPA14, SNORD22, MEIG1, DCLRE1C, ACBD7, RPP38, NMT2, FAM171A1, ITGA8, MINDY3*
	18	61158064	*Zinc Finger Protein Family*
	20	60102747	*DNAH5*
	21	31490029	*HYKK, PSMA4, CHRNA5, CHRNA3, CHRNB4, UBE2Q2, NRG4, FBXO22, TMEM266, ETFA, ISL2, RCN2, SCARPER, PSTPIP1, TSPAN3, PEAK1, LINGO1*
Boer	3	89901645	*RAP1A, INKA2, DDX20, KCND3, ST7L, CAPZA1, CTTNBP2NL, WNT2B, MOV10, RHOC, PPM1J*
	26	36351768	*HELLS, TBC1D12, NOC3L, PLCE1, SLC35G1, LGI1, FRA10AC1, PDE6C, RBP4, FFAR4, CEP55, MYOF, CYP26A1, CYP26C1, EXOC6, HHEX, KIF11*
	27	40094623	*ANGPT2, MCPH1*
Saanen	3	71857518	*DNTTIP2, GCLM, ABCA4, ARHGAP2, ABCD3, SLC44A3, CNN3, ALG14, TLCD4, RWDD3*
	11	19476181	*CRIM1, FEZ2, VIT, STRN, HEATR5B, GPATCH11, EIF2AK2, SULT6B1, CEBPZ, NDUFAF7, PRKD3, QPCT, CDC42EP3, RMDN2, CYP1B*
	14	59228427	*PENK, SDR16C5, CHCHD7, PLAG1, MOS, LYN, TGS1, TMEM68, XKR4, RP1, SOX17, RGS20, ATP6V1H*

**Figure 3:**
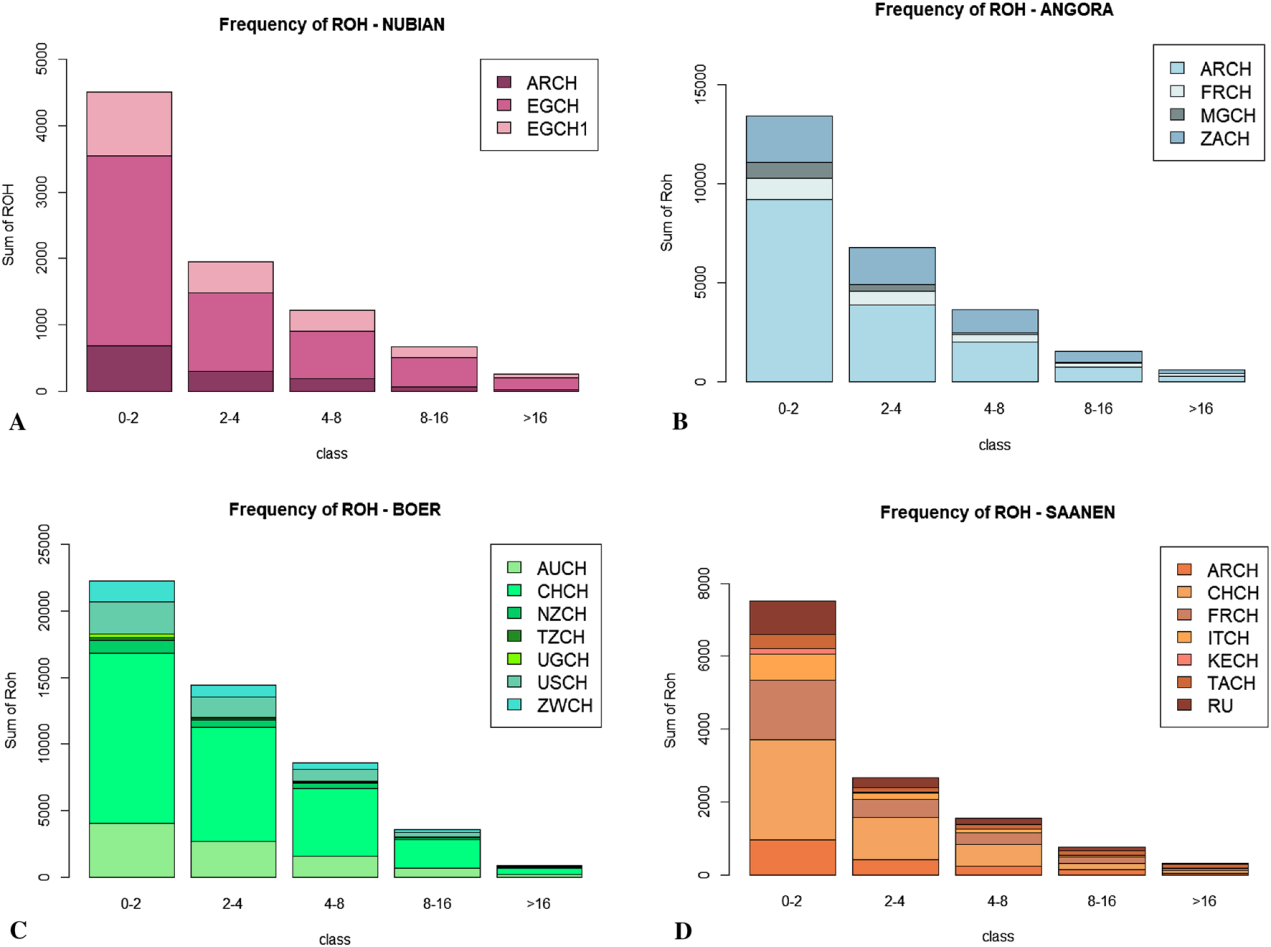
Frequency of Runs of Homozygosity for each class of length. Histograms are built with different colours for each breed (A = Nubian, B = Angora, C = Boer and D = Saanen) and every population is indicated with a different shade of the same colour.

In the Nubian breed we observed the highest number of putative markers under selection even after the Bonferroni correction (Supplementary Table 2 and Fig. 3). Since we found a strong overlap across three different analyses of a large, potential genomic region under selection in the CH6, we decided to re-analyse the Nubian dataset excluding this chromosome. After comparisons with the modified analysis, the number of outliers confirmed by both analysis with PCAdapt in Nubian breed was eight (into the CH2, CH3, CH4 and CH5, Table 2).

**Table 2:** List of candidate gene retrieved inside the genomic regions included in the interval of 1 Mb on both sides of selective sweep detected by HapFLK.

Breed	Chr	Genomic region in Mb	Putative genes
ANGORA	21	50–56	*LRFN5, C14orf28, KLHL28, TOGARAM1, PRPF39, FKBP3* *FANCM, MIS18BP1, TGM5, TGM7, LCMT2, ADAL, ZSCAN29, TUBGCP4, TP53BP1, 5S_rRNA,* *MAP1A, PPIP5K1, STRC* *CATSPER2, PDIA3, U6, ELL3* *SERF2, SERINC4, HYPK, MFAP1* *WDR76, FRMD5, GPR68*
SAANEN	25	1–5	*UNKL, C16orf91, CCDC154, CLCN7 PTX4, TELO2, IFT140* *TMEM204, CRAMP1, JPT2, MAPK8IP3, NME3, MRPS34* *SPSB3, NUBP2, IGFALS, HAGH, FAHD1, MEIOB, HS3ST6* *RPL3L, MSRB1, NDUFB10, RNF151, TBL3, NOXO1, GFER* *SYNGR3, ZNF598, SLC9A3R2, NTHL1, TSC2, PKD1, RAB26, TRAF7, CASKIN1, MLST8, BRICD5, PGP, E4F1, DNASE1L2, ECI1, ABCA3, CCNF, TEDC2, NTN3, TP6V0C, PDPK1, KCTD5, PRSS22, PRSS33, SRRM2, FLYWCH1, KREMEN2, PAQR4, PKMYT1, CLDN6, TNFRSF12A* *HCFC1R1, THOC6, BICDL2, MMP25, ZSCAN10, NF205, ZNF213, ZNF263, TIGD7, ZNF75A, OR2C1, ZNF174* *NAA60, C16orf90, CLUAP1, NLRC3, SLX4, DNASE1, TRAP1, CREBBP, ADCY9, SRL, TFAP4, GLIS2, VASN* *DNAJA3, NMRAL1, HMOX2, CDIP1, UBALD1, MGRN1* *NUDT16L1, ANKS3, SEPTIN12, ROGDI, GLYR1, UBN1* *PPL, SEC14L5, NAGPA, C16orf89*

Supplementary Figs. 1–4 show the Admixture analysis and the Supplementary Fig. 5 the cross-validation errors for each breed. These results agreed with the PCA and, together with the pairwise F_ST_ values, are in agreement with^1^ (Supplementary Table 1). In particular, both Admixture and F_ST_ confirmed the quite strong genetic structure found in the Nubian breed (Supplementary Table 1 and Supplementary Fig. 3).

### Runs of Homozygosity

The Supplementary Figs. 7, 8, 9 and 10 have the Manhattan plots for the four breeds and for each population. In the Nubian breed, based on estimation of the genomic inbreeding coefficient (FROH), it is evident that both populations from Egypt had a higher level of inbreeding compared with the population from Argentina (0.13 and 0.14 for NBN_EGCH and NBN_EGCH1, and 0.11 for NBN_ARCH, Supplementary Table 3). Looking at the distribution of ROHs per class (Fig. 3A) the NBN_EGCH group revealed a higher amount of longer ROH (8–16 and > 16 Mb) compared to NBN_EGCH1 (4–8 Mb) and NBN_ARCH (2–4 Mb).

**Figure 4:**
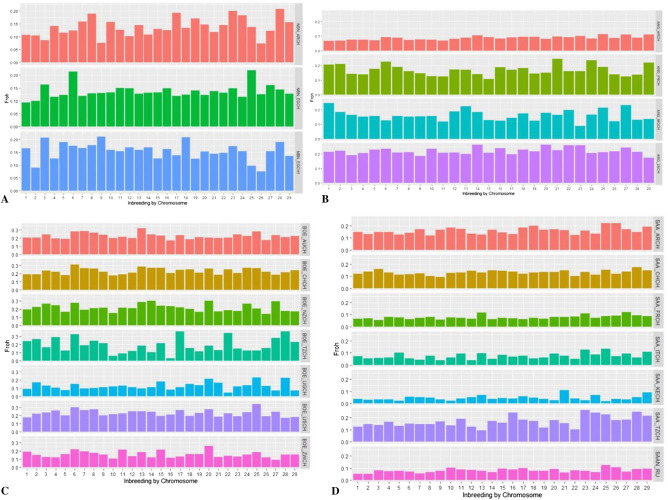
Distribution of genomic inbreeding coefficient (FROH) or ROH-based inbreeding per chromosome and for each breed. Every bar represents a chromosome and a different colour is associated with a population for every breed. A = Nubian, B = Angora, C = Boer and D = Saanen.

**Figure 5:**
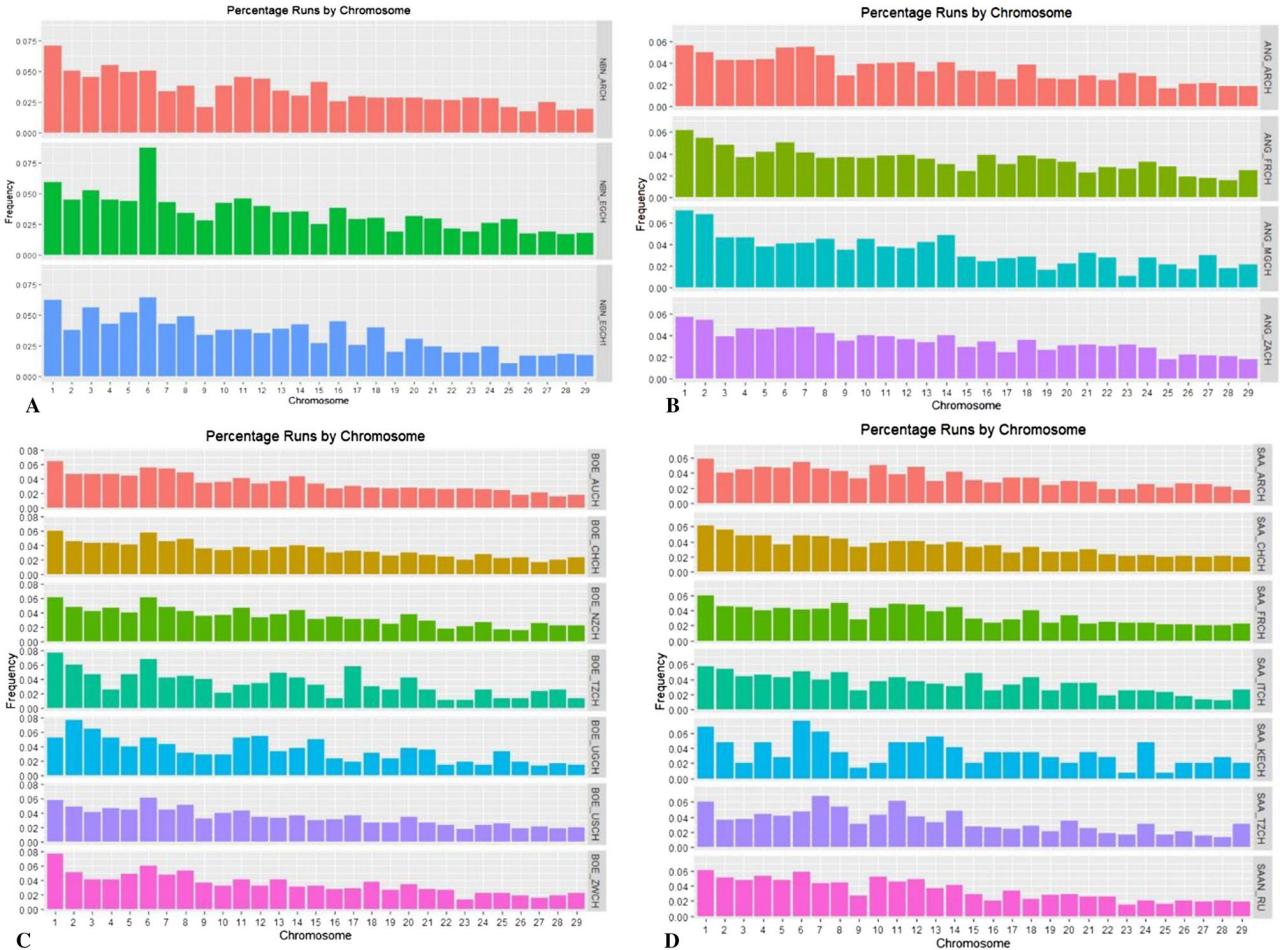
Percentage of Runs Of Homozygosity. The bars exhibit the frequency of ROH per chromosome identified in every population for the four breeds. A different colour is assigned to each population within every breed. A = Nubian, B = Angora, C = Boer and D = Saanen.

The Nubian populations from Egypt showed a high incidence of variants in ROH creating a peak on CH6, with a percentage of variants that overcomes 75% (Supplementary Fig. 9). The NBN_EGCH also showed another remarkable peak on CH25 and NBN_EGCH1 on CH18. We found similar patterns of homozygosity considering the FROH (Fig. 4A) and the percentage of ROH per chromosome (Fig. 5A).

**Figure 6:**
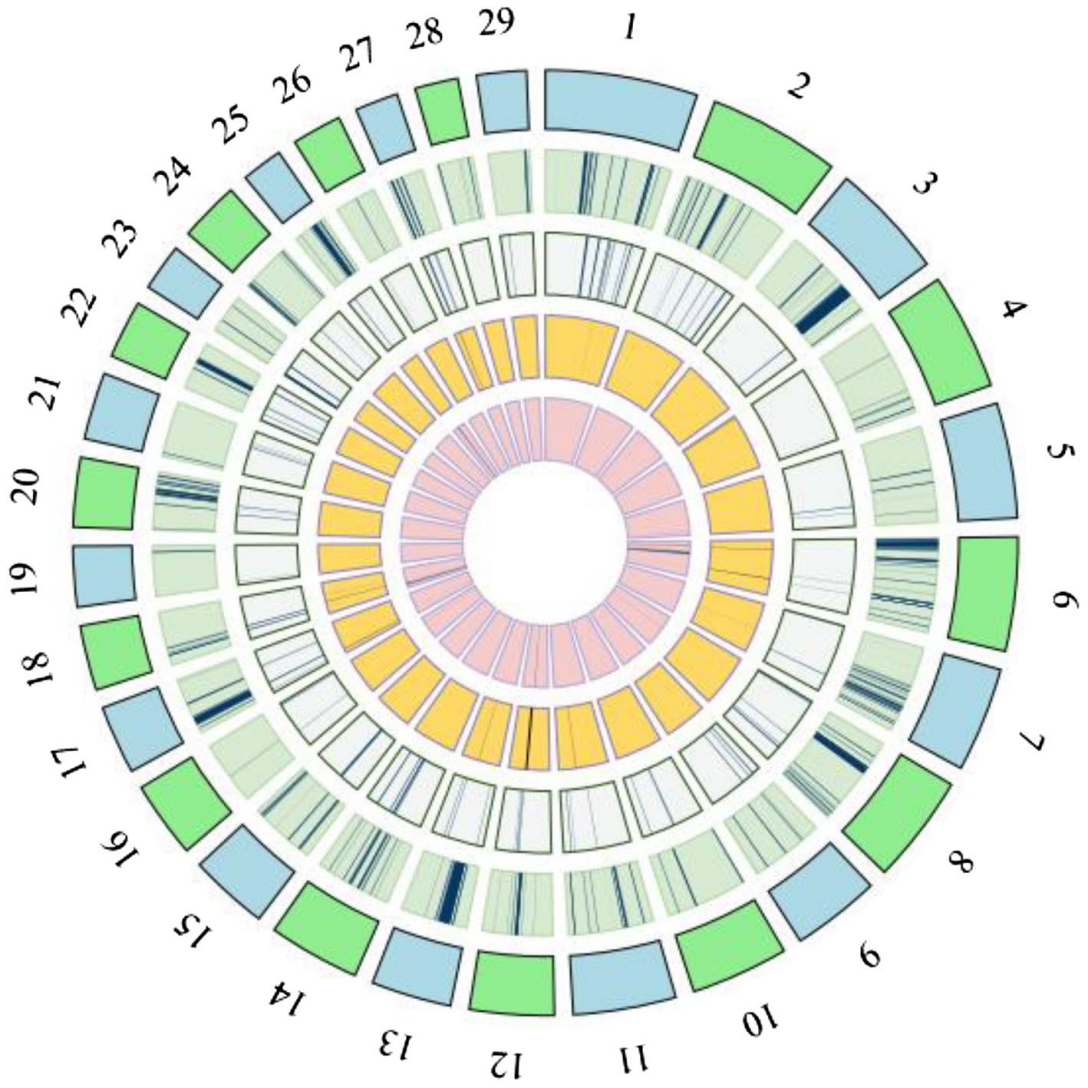
Graphical representation of the proportion of the genome covered by ROH Islands. One different colour is assigned to each breed and the order of breeds is based on the abundance of ROH Islands for a better visualization. From the most external to the centre: Boer (light green), Angora (light blue), Saanen (light orange) and Nubian (light pink).

Finally, we observed the presence of several hotspots of homozygosity (ROH islands) occurring mainly in both Egyptian populations but also one in the NBN_ARCH population (Fig. 6, Supplementary Table 4).

In the Angora breed, both African populations showed the same pattern of homozygosity, where the sum and the mean of ROH for each individual were relatively high (Supplementary Figs. 11 and 12). On the contrary, the population from Argentina had very short ROH whereas the population from France displayed an intermediate situation. Interestingly, the France population exhibited the highest FROH value on CH21 in respect to the rest of populations (Fig. 4B) and ANG_ZACH had the highest value of genomic inbreeding coefficient (FROH = 0.22, Supplementary Table 3 and Fig. 4B). The analysis by classes of length revealed an interesting result for ANG_ARCH and ANG_ZACH populations which show several homozygous segments within the 4–8 and 8–16 Mb classes (Fig. 3B). Only the ANG_MGCH population had some hotspots characterised by homozygous segments of about 2 Mb (Fig. 6 and Supplementary Table 3). The percentage of ROH per chromosome (Fig. 5B) presented a similar pattern for all Angora populations. In the Boer breed, the genomic inbreeding coefficient ranged from 0.22 to 0.1 (Supplementary Table 3) where BOE_AUCH, BOE_NZCH, BOE_CHCH and BOE_USCH had the highest values. These results were confirmed when we look at the sum and mean of ROH (Supplementary Figs. 11 and 12) and the distribution per length class, where it is evident that several segments exceed the 16 Mb in length especially in the BOE_CHCH population (Fig. 3C). In general, similar patterns of homozygosity are found in all Boer populations considering the FROH (Fig. 4C) and the percentage of ROH per chromosome (Fig. 5C). In the Saanen breed, the populations from Switzerland, Tanzania and Argentina showed the highest value of FROH, ranging from 0.12 to 0.14 (Supplementary Table 3) and in general we found few ROH in all groups (Supplementary Table 4, Fig. 6) and only few hotspots in SAA_KECH population (Supplementary Table 4). The distribution by length class showed several segments < 2 Mb in the SAA_ARCH, SAA_CHCH and SAA_FRCH populations, while segments that exceed 16 MB are found in the SAA_TZCH population (Fig. 3D). A similar pattern of homozygosity considering the FROH (Fig. 4D) was found within all Saanen populations, however SAA_TZCH had the highest value of genomic inbreeding coefficient (FROH > 0.2) on CH23, CH24, CH25 and CH28. Regarding the percentage of ROH per chromosome (Fig. 5D), SAA_KECH showed a different pattern compared with the other Saanen populations. Another interesting finding is related to the abundance of hotspots present in the genome of Boer populations. In particular, we found the same genomic region in the CH6, ranging from 85 to 86 Mb in all groups and an additional region ranging from 80 to 82 Mb was absent only in BOE_UGCH and BOE_ZWCH. Furthermore, we discovered several long ROH islands in other chromosomes shared by some populations or exclusive of a particular population that were not discovered in previous studies^6^ (Fig. 6 and Supplementary Table 4).

For instance, a ROH ranging from 21 to 25 Mb on CH13 is shared only by the BOE_NZCH and BOE_CHCH populations, and another ROH of 12 Mb on the same chromosome is exclusive to the BOE_AUCH population. This stretched segment was found to partially overlap several shorter segments found in all population excluding the African ones. Other regions on CH3, CH7 and CH8 (Supplementary Table 4 and Fig. 6) were shared or partially overlapped in BOE_AUCH, BOE_CHCH, BOE_USCH and BOE_NZCH, and some segments are partially overlapped with ROH islands found in a previous study^6^.

### Selective sweeps with HapFLK

**Figure 7:**
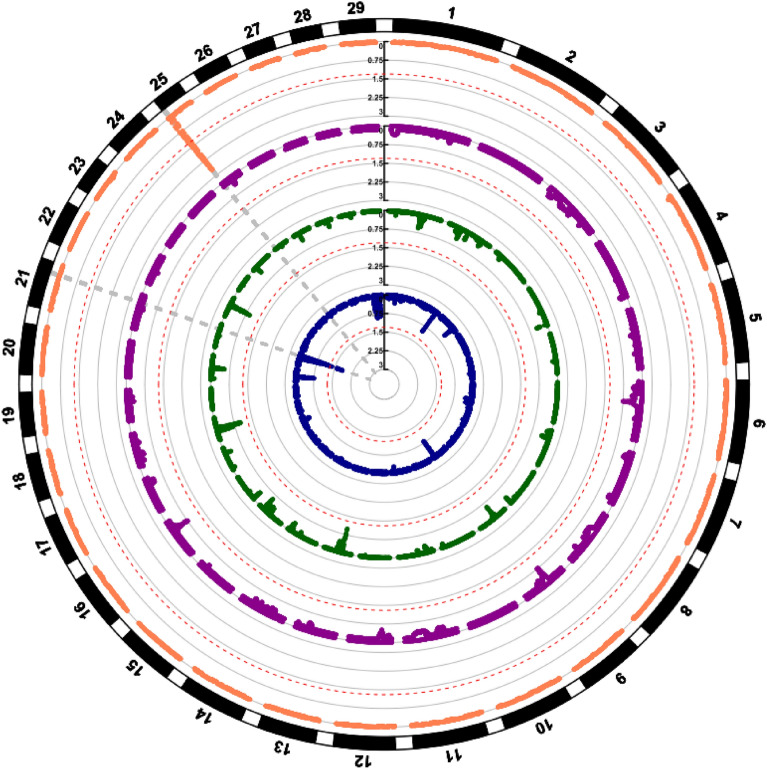
Circular Manhattan plot of selective sweeps detected with HapFLK. One different colour is the same to each breed: coral for Saanen, purple for Nubian, green for Boer and blue for Angora. The red dashed line indicates the threshold of significance of 0.05%. The grey dashed line indicates the two significant peaks in the CH25 (Saanen) and CH21 (Angora).

The HapFLK analysis detected two significant selective sweeps. The first one was a region of about 5 Mb mapping on CH25 and ranging from 1 and 5 Mb, in the Saanen breed (FDR < 0.01), whereas the second one was of about 3 Mb, spanning between 52 and 55 Mb on CH21 in the Angora breed (FDR < 0.02). For the remaining two breeds, there were no significant regions after FDR correction (Fig. 7).

However, it is necessary to remark that, while some of the peaks did not achieve the statistical threshold of 0.05, some of them showed a co-localisation with selective sweeps identified in previous studies using the same populations^6^. The corresponding putative selective sweeps and the genes falling into these genomic regions are summarised in Table 2 for the Angora and Saanen breeds. The analysis revealed several novel and well-known genes that are associated to adaptation.

Comparing the results of the three programs used in this study, we can highlight only one overlapping genomic region presumably under selection on CH6 of Nubian breed. It is worth highlighting that in the Nubian breed there were sixteen outlier SNPs falling into the putative genes under selection. These gene were *HSP40* on CH4, *PTPRB* on CH5, and *ZCCHC4*, *PPARGC1A*, *LGI2*, *SEPSECS*, *IBSP*, *CCSER1* (5 SNP), *LCORL* (2 SNP) and *KCNIP4* (2 SNP) on CH6. However, we found positional coincidences between one outlier SNP and the ROH island on CH3 of Boer breed, spanning from 79 to 95 Mb and shared by AUCH, NZCH, USCH and TZCH populations.

### Candidate gene identification and functional analysis

Candidate genes within the two Mb intervals of the putative selected regions were retrieved with the Ensembl BioMart tool. The obtained lists were further analysed using relevant literature for verifying if there were genes associated with environmental adaptation (Table 4). In all four breeds, several loci are involved in metabolism and adipogenesis, as well as feed intake, immune response and growth or which expression is affected by the availability of food. More genes indirectly related to adaptation are discovered when we checked the two large hotspots common amongst all Boer populations and summarised in Table 3.

**Table 3:** List of candidate genes retrieved inside the genomic regions included in the interval of 1 Mb on both sides of ROH islands detected by detectRUNS.

Breed	Chr	Genomic region in Mb	Putative genes
BOER	6	82–88	*STAP1, UBA6, GNRHR, TMPRSS11D, TMPRSS11A, TMPRSS11F, TMPRSS11E, YTHDC1, UGT2A2, SULT1B1, ODAM, CABS1, AMTN, AMBN, ENAM, JCHAIN, UTP3, RUFY3, GRSF1, MOB1B, DCK, SLC4A4*
	13	20–35	*PLXDC2, NEBL, SKIDA1, MLLT10, DNAJC1, COMMD3, SPAG6, PIP4K2A, ARMC3, MSRB2, PTF1A, OTUD1, KIAA1217, ARHGAP21, PRTFDC1, ENKUR, GPR158, MYO3A, GAD2, FZD8, OPTN, MCM10, UCMA, PHYH, SEPHS1, BEND7, FRMD4A, CDNF, HSPA14, SNORD22, DCLRE1C, MEIG1, ACBD7, RPP38, NMT2, FAM171A1, ITGA8, MINDY3, PTER, C1QL3, RSU1, CUBN, TRDMT1, VIM, ST8SIA6, HACD1, STAM, TMEM236, MRC1, SLC39A12, CACNB2, NSUN6, EPC1, KIF5B, ARHGAP12, ZEB1, ZNF438, SVIL, JCAD, MTPAP, MAP3K8*

All biological process terms with *P* values < 0.1, including the number of genes assigned to these terms are summarized in Table 4. The extensive analysis of the identified GO terms revealed that the identified candidate genes have been associated with diverse biological functions, such the transmission of nervous signals and metabolic processes, all of them playing a role in basic functions of the organism probably in response to environmental pressure.

**Table 4:** Gene ontology terms significantly associated with biological processes, molecular functions, and cellular components for Angora, Boer, Nubian and Saanen breeds.

Breed	Category	GO:term	*Ovis aries*	*Bos taurus*	*Homo Sapiens*	Gene name
			*P* value	*P* value	*P* value	
**Angora**	Biological process	*GO:0018149—peptide cross—linking	0.0221	0.0251	–	*TGM7, TGM5*
		GO:0035095—behavioural response to nicotine	0.0002	0.0001	0.00002	*CHRNA3, CHRNB4, CHRNA5*
		GO:0035094—response to nicotine	0.0037	0.0016	–	
		GO:0098655—cation transmembrane transport	–	0.0018	–	
		GO:0007274—neuromuscular synaptic transmission	–	0.0038	–	
		GO:0050877—nervous system process	–	–	0.0009	
		GO:0007271—synaptic transmission cholinergic	–	0.0038	0.0002	
		GO:0060084—synaptic transmission involved in micturition	0.0112	0.0105	0.026	*CHRNA3, CHRNB4*
		GO:0006940—regulation of smooth muscle contraction	0.0296	0.0245	0.0001	*CHRNB4, NMU* *CHRNA3*^+^
		GO:0045944—positive regulation of transcription from RNA polymerase II promoter	0.0841	–		*PPP1R12A, SHOX2, PHIP, NR3C1, FGF1*
		GO:0007165—signal transduction	–	0.0531	0.0005	*LINGO1, CHRN4 CHRNA3,* *PPP1R12A, CHRNA5* *CDNF*^+^ *FGF1*^+^ *NR3C1*^+^ *PPP1R12A*^+^
	Cellular components	GO:0005892—acetylcholine-gated channel complex	0.0076	0.0013	0.00008	*CHRNA3, CHRNB4 CHRNA5*
		GO:0045211—postsynaptic membrane	0.0013	0.0050	0.0004	*CHRNA3, CHRNB4, CHRNA5, KCTD16*
		GO:0005783—endoplasmic reticulum	0.0514	0.0273	0.002	*RCN2, SRP72, SRD5A3, KDR, ITGA8, ****CDNF***^**+**^
		GO: 0070161 anchoring junction	–	–	0.00004	*CHRNA3, CHRNA5, CHRNB4, KDR, KCTD16, SYT1*
		GO:0042734—presynaptic membrane	0.0626	0.0851	–	*SYT1, KCTD16*
		GO:0030054—cell junction	0.0675	0.0360	–	*CHRNA3, CHRNB4, CHRNA5, ****SYT1***
	Molecular Functions	*GO:0003810—protein-glutamine gamma-glutamyl transferase activity	0.0098	0.0101	–	*TGM7, TGM5*
		GO: 0005892-acetylcholine-gated cation-selective channel activity	–	–	0.0001	*CHRNA3, CHRNB4, CHRNA5*
		GO:0015464—acetylcholine receptor activity	0.0031	0.0006	0.0001	
		GO:0004889—acetylcholine-activated cation-selective channel activity	0.0064	0.0010	–	
**Boer**	Biological process	GO:0034653—retinoic acid catabolic process	–	0.0072	0.003	*CYP26A1, CYP26C1* *CYP26A1, CYP26C1*
		GO:0006766-vitamin metabolic process	–	–	0.004	
		GO: 0048387—negative regulation of retinoic acid receptor signalling pathway	–	–	0.005	
		GO:0016525—negative regulation of angiogenesis	–	0.0833	–	*HHEX ANGPT2*
		GO:0045444—fat cell differentiation	–	0.0998	–	*FFAR4, NOC3L*
		GO:0006898—receptor-mediated endocytosis^+^	0.0427	–	–	*CUBN, MRC1*
	Cellular components	GO:0005615—extracellular space	0.0688	0.0563	0.02	*LGI1, MOV10, RBP4, ANGPT2, ****WNT2B***
		GO: 0010008 -endosome membrane	–	–	0.009	*CUBN* *FFAR4* *MRC1*
	Molecular Functions	GO:0008401—retinoic acid 4-hydroxylase activity	–	0.0045	0.006	*CYP26A1, CYP26C1*
		GO:0001972—retinoic acid binding	–	0.0136	0.0130	
		GO:0003682—chromatin binding	–	0.0793	–	*HELLS, HHEX, NOC3L*
**Nubian**	Biological Process	GO:0051603—proteolysis involved in cellular protein catabolic process	0.0095	0.0194	0.016	*PSMB4, CTSK, CTSS*
		GO:0043486—histone exchange	0.0245	0.0481	–	*VPS72, ANP32E*
		RNA splicing	–	–	0.0001	*CELF* *DHX15* *PPARGC1A* *PRPF3* *SCNM1*
		GO:0033235—positive regulation of protein stimulation	0.0425	0.0481	–	*PIAS3, ARNT*
		GO:0007283—spermatogenesis	0.0596	–	–	*TDRKH, CELF3, HORMAD1, OAZ3*
		GO:0030574—collagen catabolic process	0.0602	–	–	*CTSK, CTSS*
		GO:0008637—apoptotic mitochondrial changes	0.0718	–	–	*NDUFS1, MCL1*
		GO:0008380—RNA splicing	0.0833	0.0605	0.001	*RBM8A CELF3 ****SCNM1*** *DHX15*^+^ *PPARGCA*^+^ *PRPF3*^+^
		GO:0006631—fatty acid metabolic process	0.0890	–	0.006	*THEM4, PPARA* *THEM5*^+^ *SNCA*^+^
		GO:0000209—protein polyubiquitination	–	0.0831	–	*KLHL42, RNF115 UBE3C*
		GO:0031290—retinal ganglion cell axon guidance	0.037	0.0410	–	*SLIT2, BMPR1B*
		GO:0060079—excitatory postsynaptic potential	0.044	–	–	*GRID2, SNCA*
		GO:0001503—ossification	0.063	–	–	*IBSP, SPP1*
		GO:0034599—cellular response to oxidative stress	0.068	–	–	*PPARGC1A, SNCA*
		GO:0031214—biomineral tissue development	–	0.0386	0.005	*SPP1, MEPE, IBSP*^+^
	Cellular components	GO:0005737—cytoplasm	0.0399	0.0415	0.0007	*PIAS3, PTPRR, RBM8A SETDB1, TUFT1,* *CELF3, PRUNE1, OTUD7B, ARNT,* *PTHLH, PSMB4, PSMD4, CNOT2, PRPF3, POGZ,* *TXNIP, TNFAIP8L2, HORMAD1, S100A11*
			–	–	0.0007	*PPARGC1A,* *ENSA* *HPGDS* *HHEX* *OAZ3* *PLEKHO1* *PKD2* *THRDE*
			–	0.0223	0.0007	*SLC34A2, KCNIP4, NCAPG, RBPJ, SOD3, MED28, HERC5, SEPSECS, DHX15, LAP3, SLIT2, HERC6, SNCA*
		GO:0032587—ruffle membrane	–	0.0364	–	*THEM4, PIP5K1A, PLEKHO1*
		GO:0016020—membrane	–	0.0407	–	*IBSP, NCAPG, PKD2, BMPR1B, MED28, SNCA*
		GO:0015629—actin cytoskeleton	0.0231	0.0325	–	*NCAPG, PDLIM5, SNCA*
		GO:0005615—extracellular space	0.0511	–	–	*IBSP, SPP1, SLIT2, SOD3, SNCA*
	Molecular Functions	GO:0016290—palmitoyl-CoA hydrolase activity	0.0324	0.0374	–	*THEM5 THEM4*
		GO:0003700—transcription factor activity sequence specific DNA binding	–	0.0259	0.007	*GABPB2, KLF7, ARNT, CREB1, MNX1, PPARA, RFX5, MYRFL*
		GO:0019212—phosphatase inhibitor activity	–	0.0031	0.02	*ANP32E, ENSA*
		GO:0043394—proteoglycan binding	0.0451	–	0.0004	*CTSK, CTSS, SLIT2*^+^
		GO:0008270—zinc ion binding	0.0602	–	0.002	*ZDBF2, RNF32* *PIAS3, RNF115* *CPO, SETDB1, RORC OTUD7B, PPARA TRHDE, DYTN,* *KLF7*^+^ *SNCA*^+^
		GO:0003677—DNA binding	0.0915	0.0494	0.0002	*KLF7, SETDB1* *RFX5, POGZ, OTUD7B MYRFL, CERS2* *ARNT, PPARA* *VPS72* *RORC*^+^ *CREB1*^+^ *HHEX*^+^ *MNX1*^+^ *PPARGC1A*^+^ *RBPJ*^+^
		GO:0005509—calcium ion binding	0.0075	0.0194	–	*HPGDS, MMRN1, KCNIP4, SLIT2, PKD2, SNCA* ***STIM2, KCNIP4***
		GO:0051219—phosphoprotein binding	–	0.0142	–	*PKD2, SNCA*
		GO:0005267—potassium channel activity	–	0.0280	–	*KCNIP4, PKD2*
**Saanen**	Biological process	* GO:0006308—DNA catabolic process	–	0.0283	–	*DNASE1, DNASE1L2*
		Retinal metabolic process	–	–	0.0006	
		* GO:0016567—protein ubiquitination	–	0.0455	0.003	*SPSB3, TRAF7, CCNF, RNF151*
		* GO:0006308—DNA catabolic process	0.0123	0.0123	0.01	*DNASE1, DNASE1L2*
		GO:0035556—intracellular signal transduction	0.0560	–	–	*LYN, PRKD3 ARHGAP29*
		GO:0042574—retinal metabolic process	–	0.0193	0.00067	*SDR16C5 CYP1B1*
		GO:0042572—retinol metabolic process	–	0.0345	–	
		GO:0045494—photoreceptor cell maintenance	–	0.0551	–	*RP1, ABCA4*
		GO: 0007603 -phototransduction visible light	–	–	0.01	*RP1, ABCA4*
		GO:0071407—cellular response to organic cyclic compound	–	0.0625	–	*RGS20, CYP1B1*
		GO:0009636—response to toxic substance	–	0.0621	–	*EIF2AK2, CYP1B1*
		GO:0001750—photoreceptor outer segment	–	0.0820	–	*RP1* *ABCA4*
	Cellular component	GO:0005634—nucleus	0.047	0.0331	0.000001	*MGRN1, CCNF, TSC2, PKMYT1, PKD1, NMRAL1, NUBP2, TFAP4, NTHL1, DNAJA3, UBN1, ZSCAN10, CDIP1, MSRB1*
		GO:0005829 -cytosol	–	–	0.002	*ABCD3* *CDC42EP3* *DNAJA3* *LYN* *MOS* *CCNF* *TSC2* *TELO2* *ZNF174*
		* GO:0031931—TORC1 complex	–	0.0352	–	*MLST8, TELO2*
	Molecular Functions	GO:0005524—ATP binding	0.0473	0.0473	0.01	*LYN, MOS, ABCD3, PRKD3, ABCA4, EIF2AK2*
		GO:0042626—ATPase activity coupled to transmembrane movement of substances	0.0547	–	–	*ABCD3 ABCA4*
		GO:0005096—GTPase activator activity	–	0.0579	–	*CDC42EP3, RGS20, ARHGAP29*
		* GO:0046872—metal ion binding	–	0.0085	0.000003	*ZNF263, ZNF174, FAHD1, ZNF75A, NUBP2, GLIS2, NTHL1, ZSCAN10, ZNF205, E4F1, HAGH, ZNF213, MSRB1* *DNAJA3*^+^ *ARHGAP29*^+^ *CDIP1*^+^ *MGRN1*^+^ *PKMYT1*^+^ *RNF151*^+^
		* GO:0003700—transcription factor activity sequence-specific DNA binding	–	0.0240	–	*ZNF263, ZNF174, TFAP4, ZSCAN10, E4F1, ZNF205, ZNF213*

*Genes from HapFLK analysis; ^+^genes present only Homo sapiens annotation; In bold: genes present only in *Bos Taurus* annotation.

## Discussion

Environmental factors are one of the forces influencing agricultural and the livestock sectors. Animals exposed to stressful environments exhibit various adaptive mechanisms, such as behavioural, physiological, endocrine, cellular, metabolic and biochemical for minimising the stressful conditions. Thus, adaptation is the natural strategy to ensure both welfare and efficiency. The action of selection leaves signs along the genome as responses to environmental and anthropogenic pressures that can be revealed using specific methodologies and bioinformatic tools. We applied three complementary methods for detecting a wider range of candidate genes that can be further investigated. The analysis with PCAdapt revealed, in Boer and Saanen breeds, a few markers potentially under selection for environment adaptation, whereas Angora and Nubian breeds showed several outliers distributed along different chromosomes.

Our findings led to the identification of target genes related to adaptation and more specifically to response at the temperature stress, energy homeostasis, photoperiod, immune/inflammatory response, reproductive and production traits. The responses to stress include not only reactions to extreme cold and hot temperatures but also the ability to adapt to harsh environmental conditions, such as poor-quality forage or water scarcity. In African indigenous chickens, the *TOGARAM1*locus, involved in the assembly of non-motile cilia and thus essential for cellular signal transduction, was found affected by heat-shock^7^. In fact, heat can induce a rapid loss of these important organelles^8^, deducing that this gene may play an important adaptive role in alleviating this effect in high temperature conditions. *PDIA3* regulates cell growth and death according to oxygen concentrations and this gene was implicated in the thermal acclimatisation process in ovine liver tissue^9^, and in sperm–egg fusion in sheep and cashmere goats^10^. These genes were detected in Angora goat populations on CH13 near to the SNP outlier and within the selective sweep on CH21. The heat-shock protein 40 (*HSP40*) and the heat shock protein family A (*HSP70*) member 14 (*HSPA14*) belong to the heat-shock proteins (HSP) family, involved in cellular responses and for protein homeostasis and survival under stress conditions. In particular, HSP70 gene has been linked with heat tolerance and higher milk production in cattle^11^. These findings indicate the putative effects of selective pressure on this gene family favouring animals with better thermotolerance, performance and stress resilience^12^. *TRPA1* is a member of the transient receptor potential (TRP) superfamily of ion channels. Studies in mice suggested that *TRPA1* channels mediate cold temperature sensing in mammalian vagal sensory neurons, evoking major protective reflexes and thermoregulatory responses^13^. This gene was found in the putative selective sweep on the CH25 of Saanen goat: in this breed, populations are from different climatic areas (continental and temperate, following the Köppen–Geiger Climate Classification; Table 5), indicating a plausible association with thermal stress and cold adaptation. Another interesting candidate gene is *TRHDE,* a gene implicated in energy homeostasis, body temperature regulation^14^, in particular adaptation to hot arid environments in goats^15^ and high-altitude in Ethiopian sheep^16^.

**Table 5:** Breed and population code, country and number of samples used in the study. We added the Köppen–Geiger Climate Classification for further considerations.

Breed	Population code	Country	Number of animals	Total	Köppen–Geiger climate classification
NUBIAN	NBN_ARCH	Argentina	15	99	Cwa
	NBN_EGCH	Egypt	64		BWh
	NBN_EGCH1	Egypt	20		BWh
ANGORA	ANG_ARCH	Argentina	285	366	BWk
	ANG_FRCH	France	26		Cfc
	ANG_MGCH	Madagascar	7		BSh
	ANG_ZACH	South Africa	48		BSk
BOER	BOE_AUCH	Australia	61	332	BSh
	BOE_NZCH	New Zealand	14		Cfb
	BOE_CHCH	Switzerland	189		Dfb
	BOE_UGCH	Uganda	5		Aw/As
	BOE_USCH	USA	34		Cfb
	BOE_TZCH	Tanzania	4		Aw/As
	BOE_ZWCH	Zimbabwe	25		Cwc
SAANEN	SAA_ARCH	Argentina	18	196	Cwa
	SAA_ITCH	Italia	24		Cfa
	SAA_FRCH	France	56		Cfc
	SAA_KECH	Kenia	2		Cfc
	SAA_SWCH	Switzerland	44		Dfb
	SAA_TZCH	Tanzania	19		Cwc
	SAA_RU*	Russia	33		Dfb

*This population is not from the AdaptMap dataset. The Köppen–Geiger Climate Classification Map ranged from the year 1980 to 2016. Legend of the Map: Aw = Tropical/Dry winter As = Tropical/Dry summer; BWh = Arid/desert/hot; BWk = Arid/desert/cold; BSh = Arid/Steppe/Hot; BSk = Arid/Steppe/Cold; Cfc = Temperate/No dry season/Cold summer; Cfb = Temperate/No dry season/warm summer; Cwc = Temperate/Dry winter/Cold summer; Cwa = Temperate/Dry winter/Hot summer; Cfa = Temperate/No dry season/Hot summer; Cfc = Temperate/No dry season/Cold summer; Dfb = Continental/No dry season/Warm summer.

From https://en.wikipedia.org/wiki/K%C3%B6ppen_climate_classification.

In this study, we identified several genes that are involved in the lipid metabolism, adipogenesis and feed intake, directly or indirectly related to energy balance. *CLVS2* participates in regulation of foetal development in cattle that underlie the effects of early maternal nutrient restriction^17^. Interestingly, if we compare the Nubian populations from Egypt and Argentina, we can observe a clear discrepancy on the resources available in terms of food and water, since Egypt is a country characterised by a hot and dry climate. The same observation applies to Angora, as the populations from Argentina and South Africa, sampled in an arid zone, shared a common result in the analysis of ROH, with a greater number of medium-large homozygote segments (4–8 Mb), and thus suggesting a certain degree of selection that is occurred not recently. The *CCSER1* locus was previously associated with the feed efficiency in beef cattle^18^ and in sheep^19^. It is worth highlighting that this gene lies close to well-known genes associated to body size, growth and height and it falls within the large genomic region we identified in Nubian. Our findings are confirmed by previous studies that reported a strong positive selection around the *ABCG2, SPP1, LAP3, NCAPG, LCORL, PKD2, IBSP, and MEPE* genes in domestic goats and sheep^19^.

In all breeds, we also pinpointed genes under selection for altitude adaptation, like *DCLRE1C, FANCM* and *PPP1R12A* in the Angora population, *MCPH1* and *ANGPT2* in the Boer group, *TRHDE* and *IBSP* in the Nubian and *TRAP1* (as discussed above), *CEBPZ, HMOX2, NMRAL1* in Saanen. *HMOX2*, involved in hypoxia response and the neighbouring *NMRAL1*, involved in synthesis of nitric oxide, are thought to be contributors to adaptation to high altitude in humans^20,21^. Edea and co-workers^22^ observed *PPP1R12A* to be associated with high altitude adaptation in Ethiopian sheep, and previous studies have already demonstrated that hypoxia increased phosphorylation of this gene^23^.

One of the most important and predictable environmental variations is seasonality in temperate zones, based on photoperiodism over the year^24^. Two out of four breeds studied here (Angora and Saanen) showed several candidate genes linked to physiological adjustments driven by photoperiodism. For example, *CLOCK* is one of the most important genes that controls circadian rhythms by regulating various physiological functions including sleep, body temperature, blood pressure, endocrine, cardiovascular and immune systems^25^. The *CLOCK* gene also has an impact on energy metabolism influencing the rhythms of feeding behaviour^26^. In the Angora breed, we found the *KDR* gene that is related to coat colour, and that falls into the same genomic segment that contains other genes like *SRD5A3, TMEM165, PDCL2, EXOC1L, CEP135, SCFD2, FIP1L1, LNX1, PDGFRA, CLOCK, NMU* and *EXOC1* found under selection in Reggiana cattle^27^.

Stress can affect the immune system by inducing alteration of inflammatory processes and the animal’s inflammatory response is a survival mechanism to cope with pathogenic or non-pathogenic challenges^28^. Oxidative stress is considered an imbalance between oxidant and antioxidant status and considered one of the key factors causing the weakening of immune system in animals that have undergone heat stress. Macrophages and neutrophils play an important role in innate immunity by producing nitric oxide. *NMRAL1*, a candidate gene in Saanen breed, is related to the synthesis of nitric oxide and maybe could play a role in the activation of inflammatory processes. Several studies suggest that exposure to heat results in oxidative stress, thus promoting cytotoxicity^29^ and cellular damage^30^. It is remarkable how we detected, again in Saanen breed and as previously mentioned, the gene *HMOX2* that is involved in the antioxidant response like its homologous *HMOX1* gene that has been reported to play a role in "tissue tolerance"—the ability to resist pathogens, inflammation, or oxidative stress-mediated damage during infection or inflammation in humans^31^. This intersection amongst oxidative imbalance, immune, and physiological responses has been already described in sheep^28^.

In the selective sweep of CH21 (Angora breed), we found several interesting genes like *FKBP* and *LRFN5. LRFN5* is involved in immune system in cattle^32^. It is worth noting that this gene maps inside a QTL region identified in sheep and involved in scrapie infection, a disease of the nervous system^33^. In the same genomic region of CH21, we also found *MAP1A*, that allows the maintenance and restructuring of adult neurons^34^ and maps inside a QTL affecting classical scrapie incubation time in a population of scrapie-infected^35^.

The thermal environment is the largest single stressor affecting the efficiency of animal production systems. Some evidence from field studies in sheep^36^ highlighted that the physiological and behavioural adaptations that allow animals to maintain homeothermy, negatively impact their growth, welfare and reproduction. Therefore, it is not surprising that our analysis revealed within the selected regions several genes that correlated to reproduction traits like fertility and productive performances, including growth and development. The expression of *NR3C1* was explored in the ovine uterus^37,38^ discovering the crucial role of endometrial functions during early pregnancy in sheep. The effects of environmental stressors are also evident in male reproductive performances. Testicular thermoregulation is imperative to produce healthy viable spermatozoa^39^. We found in our study *SCAPER, SEPTIN12, RODGI*, selected in the Saanen group whereas *HORMAD1, TDRKH, CELF3* and *OAZ3* in the Nubian group, all genes related to spermatogenesis and fertility in mice^40^ and humans^41^.

It has been reported that a reduction in wool fiber diameter is a consequence to deteriorating food quality and availability^42^. Based on our results, we observed selection signatures in the *FGF1* gene, a member of the fibroblast growth factor family and involved in the growth and development of various tissues and organs. *FGF*1 was also the target gene of a miRNA that had an effect on growth and development of hair follicles in sheep^43^.

Our results showed several novel and established genes that are correlated with milk, meat and growth traits (development, body size and height). Amongst them, the most important were retrieved in the selective sweep on the CH6 of Nubian populations, that includes *CCSER1, LAP3, MED28, FAM184B, DCAF16, NCAPG, LCORL, SLIT2, PACRGL, KCNIP4, PPARGC1A*, these loci were described in cattle^44^, sheep^19,45^ and goats^46^.

The large hotspot retrieved on CH6 and shared by all populations investigated, contained genes associated to reproduction and immune resistance. GnRHR regulates the production of gametes and gonadal hormones and it is important for reproduction control in buffalo, cattle and goats^47–49^. Interestingly, in a review investigating the evolution of GnRHR family genes and its receptors, the following genes surrounding the mammalian GnRHR1 (*STAP1, UBA6, GnRHR, TMPRSS11D, TMPRSS11A, TMPRSS11F, TMPRSS11E and YTHDC1*) and retrieved in our analysis, are conserved in human, mouse and other vertebrates^50^ suggesting that they can affect the same trait.

In the Gene Ontology (GO) analysis, all biological processes are related with neurological functions and the nervous system in Angora breed. The functional annotation exacerbates neurological pathways involving behavioural acetylcholine-mediated responses. Acetylcholine (ACh) is the neurotransmitter used for muscular activation and all biological processes converge to cholinergic transmission. These chemical signals act on regulation of smooth muscle contraction (GO:0006940) and as a component of presynaptic (GO:0042734) and postsynaptic (GO:0045211) membranes. In fact, the key genes in these pathways are *CHRNA3, CHRNB4, CHRNA5*, which are nicotinic acetylcholine receptors. Researchers demonstrated that mild hypoxia decreased ACh synthesis and the amino acid metabolism^51^. In this breed, we found genes related to hypoxia and in general to adaptation to harsh environments, thus suggesting that the nervous system regulates many processes that can affect the efficiency in maintaining homeostasis. Three out of four populations included in the Angora dataset were sampled in arid cold and with desert or steppe (Argentina and South Africa) and hot (Madagascar) environments– thus exposed to extreme conditions, whereas the French population is the only one that comes from a temperate climate.

The Boer, Saanen and Nubian groups shared a GO associated with retinoic acid activity pathway. Retinol is vitamin A, a fat-soluble compound that is required for vision, cellular proliferation and differentiation. Studies in cattle demonstrated that it regulates intramuscular adipose tissue and muscle development^52^. Retinol metabolism pathway is also involved in feed efficiency in livestock^53^ and in normal immunologic function^54^. In the Boer breed, there is another interesting GO regarding angiogenesis. As we discussed above, angiogenesis is involved in some high-altitude adaptation responses.

Boer populations belonging to this dataset come from many different climatic zones, with a wide range of environmental variations; for example, the population from Switzerland originated from a sample site with continental climate that exposed individuals to different temperatures in winter and summer, whereas the population from Australia is exposed to hot weather. Intriguingly *WNT2B*, that is a potential target gene in wool follicle development, showed a footprint of selection in this breed that is not farmed for this purpose, suggesting that it could be related to the local adaptation of some populations to the temperature regime. If additional studies would verify that a selective pressure is acting on this locus in this breed, it could be a further confirmation that natural selection continues affecting and leaving detectable traces.

In Saanen populations, the GO revealed also photoreceptor outer segment/photoreceptor cell maintenance processes that together with retinol acid activity pathway can lead us to hypothesize that some part of the genome is triggering mechanisms to the protection/maintenance of cells belonging to the visual system, and maybe adapting it to a new and variable condition of light. Moreover, in this breed, we see several genes related to DNA repair and oxidative stress that are also related to solar radiation.

In Nubian breed, the GO results showed links with energetic metabolism, protein, and fatty acids synthesis regulation, but also catabolic processes (proteins and collagen) and cellular response to oxidative stress. The phosphatase inhibitor activity is a remarkable finding, because this impedes the target enzyme activity, avoiding the protein and cellular lysis. Consequently, as the protein phosphatases, it negatively regulates the HPSs proteins^55^. Inhibitors of this protein can prevent cell and protein damage in response to thermal stress. The two populations from Egypt are subjected to many stressful factors, in particular to thermal stress, since they were sampled in arid, desertic zones with very hot temperatures, whereas the population from Argentina comes from a temperate climate, thus the local adaptation to bio-climatic conditions is evident.

Although our objective was not to compare the effectiveness of each program used to carry out the analysis for discovering genomic regions under selection, it has been possible to see a general agreement on the evident clues of the adaptative processes that synergically activate a complex gene network. In our study, we found well known loci that have been identified in previous studies in goats as well as novel genes that showed implications for biogeographical adaptation described in other species, in particular on other ruminants. Most of these studies focused on local or indigenous populations, thus highlighting a probable population-specific selection footprint. Detecting regions under selection is a complex task, and this is reflected from the intricate connections amongst genes and biological processes. Taken together, our findings indicated that natural selection operated and continues acting in commercial goat breeds despite human intervention. Moreover, they provided evidence of selection that may be specific to one or few populations (local adaptation), and this information could be useful to identify both causal variants that are involved in a particular phenotype or important adaptive traits and the affected genes. Further investigating the detected genes will shed light on the complex mechanisms involved in the adaptation process, and provide information on putative favourable variants. Such information could be use in selection/conservation programs, also via new breeding technologies.

## Materials and methods

Since the aim of this work was to detect loci that are under natural selection in artificially selected goat breeds, we addressed this issue choosing the follow four commercial and transboundary breeds: Angora, Boer, Saanen and Nubian. Each of them is known to be selected for a specific productive trait (wool, meet, milk and dual-purpose, respectively) and were transported over centuries in different countries, thus exposed to multiple environmental variables with respect to their original countries. Considering these characteristics, the four breeds studied meet our goal.

### Sampling, genotyping and quality control

**Figure 8:**
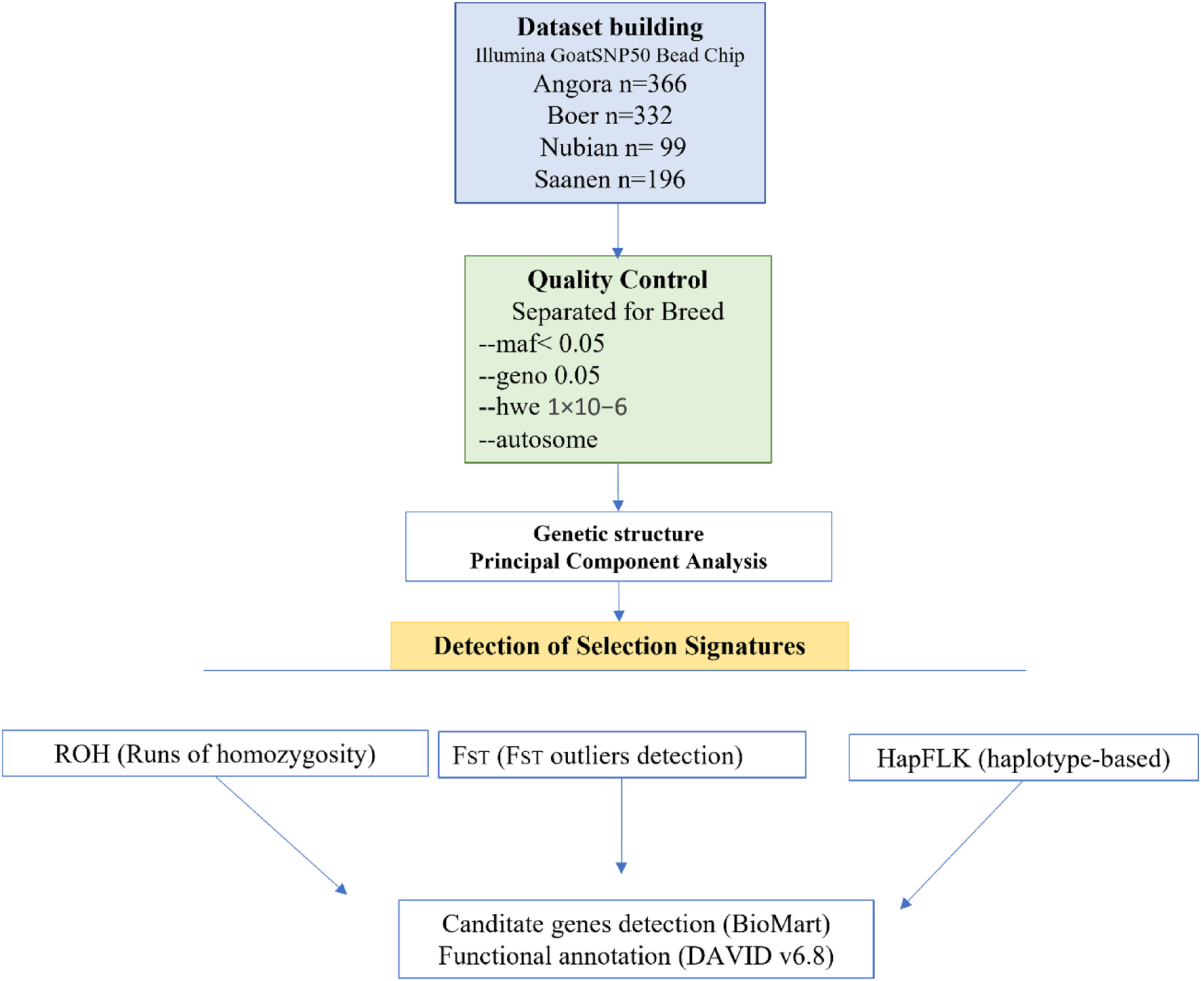
Graphical representation of workflow followed for detecting genetic signatures for adaptation.

Figure 8 describes the workflow followed for detecting genetic signatures in our dataset. Genotypic data were gathered for goat breeds with a worldwide distribution. A total of 993 individuals belonging to four commercial breeds with a worldwide distribution were included in the analysis: Angora (n = 366), Boer (n = 332), Nubian (n = 99) and Saanen (n = 163) breeds from AdaptMap project (http://www.goatadaptmap.eu/^56^) and 33 genotypes of Russian Saanen goat^57^. All individuals were previously genotyped with the Illumina GoatSNP50 BeadChip^3^. The raw dataset was updated to the latest goat genome map (ARS1.2) and the quality control was carried out using Plink v1.9110^58^ (Table 1) excluding SNPs unmapped or mapped into the sex chromosomes, SNPs with minor allele frequency < 0.05%, markers that failure the Hardy–Weinberg test at a specified significance threshold of 1 × 10^−6^, and SNP with call rate < 95%. Since we investigate breed-specific selection signatures related to adaptation, this procedure was repeated for all the four datasets, yielding a total of 44,655, 46,124, 44,800 and 47,325 for Angora, Boer, Nubian and Saanen, respectively. A first PCA analysis carried out with SNPrelate^59^ R package to explore the genetic structure Egyptian Nubian samples revealed a strong population divergence between individuals. Thus, we split the Nubian in two subpopulations: EGCH and EGCH1 (Table 1). Further analyses of this breed were done considering three populations in the Nubian dataset.

For better understand the genetic background of the four breeds, we used Arlequin 3.5.2 program^60^ to calculate the pairwise F_ST_ and the Admixture 1.3^61^ for the clusters analysis testing a number of clusters (K) equal to the number of populations composing each breed plus 3.

### Data analysis

With the goal of leveraging the potential of the methods in capturing signals for regions under selection, we combined three complementary statistics with specific programs that can be used to calculate them^62^: Runs Of Homozygosity (ROH), FST-outliers detection and HapFLK methods were applied. ROH analysis compares genomic data within populations, and it is based on the detection of reduced local variability. The last two methods rely on the degree of differentiation due to locus-specific allele frequencies among populations and can be grouped into Single site (FST-outliers) and haplotype-based differentiation (HapFLK), respectively. To carry out these analyses DetectRUNS 0.9.4 package (R core 4.1), PCAdapt^63^ and HapFLK program v1.3^64^ were used.

### Runs of homozygosity

ROH are defined as two contiguous identical by descent (IBD) stretches of homozygous genotypes of a common ancestor present in an individual and inherited from both of its parents. The identification and characterization of ROH allow to reveal the population structure as well as footprints of natural and/or anthropogenic selection^62^. This analysis was carried out by using the R package DetectRUNS 0.9.4 package (R core v4.1) applying the “sliding windows” function and with the following setting: windowSize = 15, threshold = 0.1, minSNP = 15, ROHet = FALSE, maxOppWindow = 1, maxMissWindow = 1, maxGap = 10^6^, minLengthBps = 1,000,000, minDensity = 1/10,000. We identified both ROH (length per class of ROH, total length per chromosome and sum at individual level, the frequency of SNP in each segment and visualising the homozygous segments per classes of length) and ROH islands (frequency of ROH at population level) ROH islands were plotted for all breeds using Biocircus^65^ package in R v.1.3.1073 (R core team 2020).

### F_ST_ outliers detection

PCAdapt is a R package that uses statistical tools for outlier detection based on Principal Component Analysis (PCA). Briefly, this program tests how much each variant is associated with population structure, assuming that outlier variants are indicative of local adaptation. We determined the optimal number of PCs as recommended by Luu and co-workers^63^ using the graphical PCAdapt function and keeping PCs that correspond to eigenvalues to the left of the lower straight line in the screeplot (Supplementary Fig. 6) according to “Cattell’s rule”, that were 10 for all breeds. The P-values associated to the outlier variants were corrected with Bonferroni with a threshold of 0.05.

### HapFLK analysis

With the HapFLK program v1.3 the loci under selection are revealed by comparing the genetic differentiation amongst the analysed populations with respect to the neutral drift model identifying genomic regions or loci showing deviations from neutrality (selective sweeps). The analysis was performed using the scripts available at https://forge-dga.jouy.inra.fr/projects/hapflk. The number of k (haplotype clusters) that better fits our data and estimated using the cross-validation procedure included in the fastPHASE software of^66^ was 35 for all breeds. The hapFLK statistic was computed as an average of 30 EM iterations to fit the Linkage Disequilibrium (LD) model. The *P* values obtained using the “Scaling_chi2_hapflk.py” script available at https://forge-dga.jouy.inra.fr/documents, were corrected for multiple comparisons using the false-discovery rate (FDR) method in R and SNPs (with a *P* value ≤ 0.05)were considered significant. Graphical representations of the Manhattan plots of the significant outliers and the selective sweeps retrieved with PCAdapt and HapFLK were done using CMplot package in R v.1.3.1073 (https://github.com/YinLiLin/R-CMplot).

### Searching for candidate genes and pathways related to adaptation

The next step was to compare results from the three methodologies and to verify if identify genomic regions overlapped. Then, a screening within 1 Mb downstream and upstream of each significant marker was applied to pinpoint positional candidate genes, using Ensemble BioMart *Capra hircus* ARS1 data mining tool (https://m.ensembl.org/info/data/biomart/; Capra_hircus—Ensembl genome browser 108). Loci were investigated for each breed, focusing on previous studies about selection signatures mainly in goat, but also in other livestock species like sheep and cattle, since that the annotation of some genes in goat is still lacking or poor. Pathway enrichment analysis was performed to explore possible pathways involved in environmental adaptation. The genes identified from Ensemble BioMart were stored to perform a functional annotation using *Ovis aries*, *Bos taurus* and *Homo sapiens* databases by DAVID v6.8^67^.

## Supplementary Information

Supplementary Information 1.

Supplementary Information 2.

Supplementary Information 3.

Supplementary Information 4.

Supplementary Information 5.

Supplementary Information 6.

Supplementary Information 7.

## Data Availability

The datasets analysed during the current study is available in the Dryad Repository (https://doi.org/10.5061/dryad.v8g21pt).
