# Supplementary figures and images for: Discovering novel clues of natural selection on four worldwide goat breeds
Source: Sci Rep. 2023 Feb 6;13:2110. doi: 10.1038/s41598-023-27490-x (PMC9902602; doi:10.1038/s41598-023-27490-x)

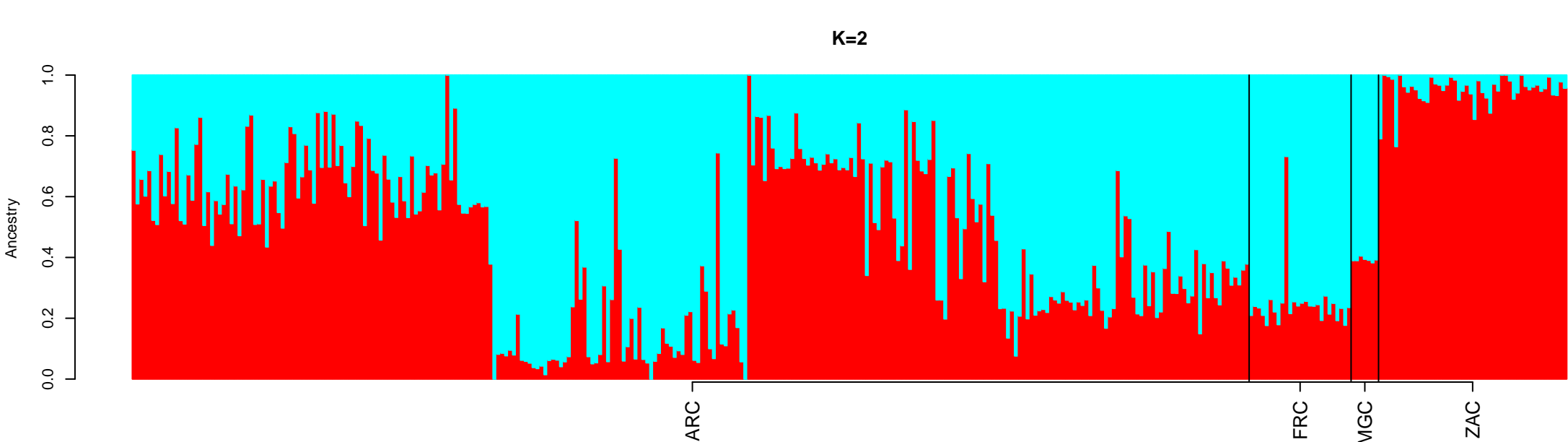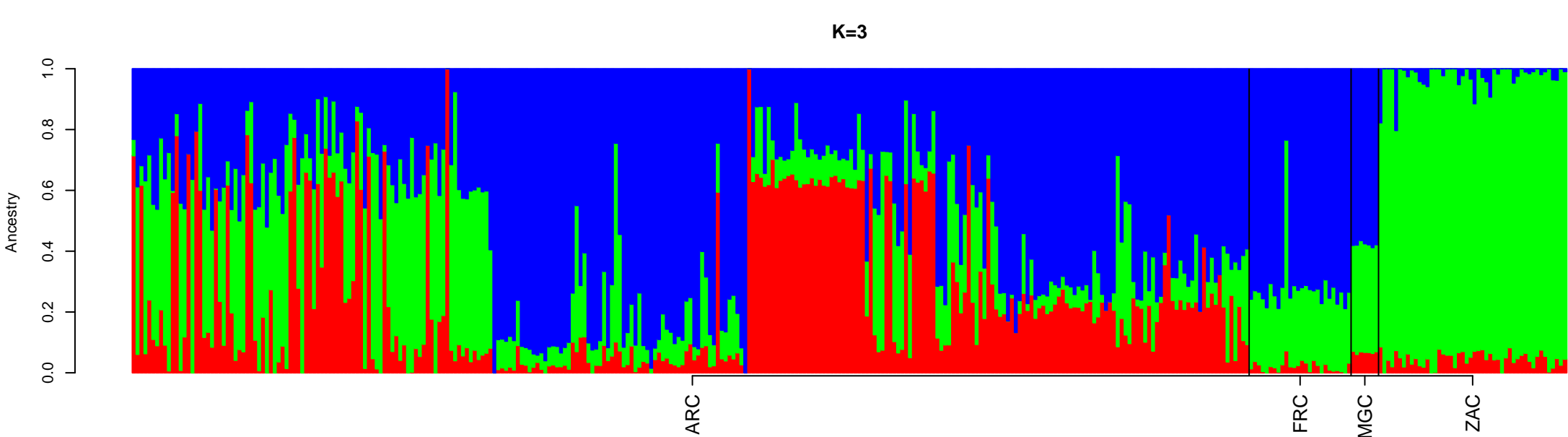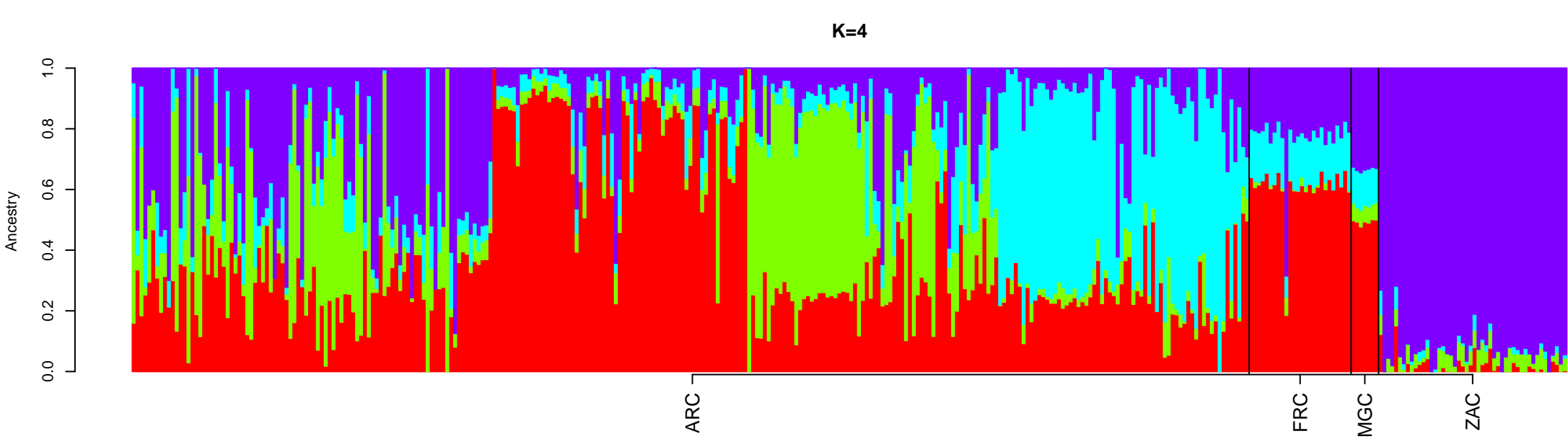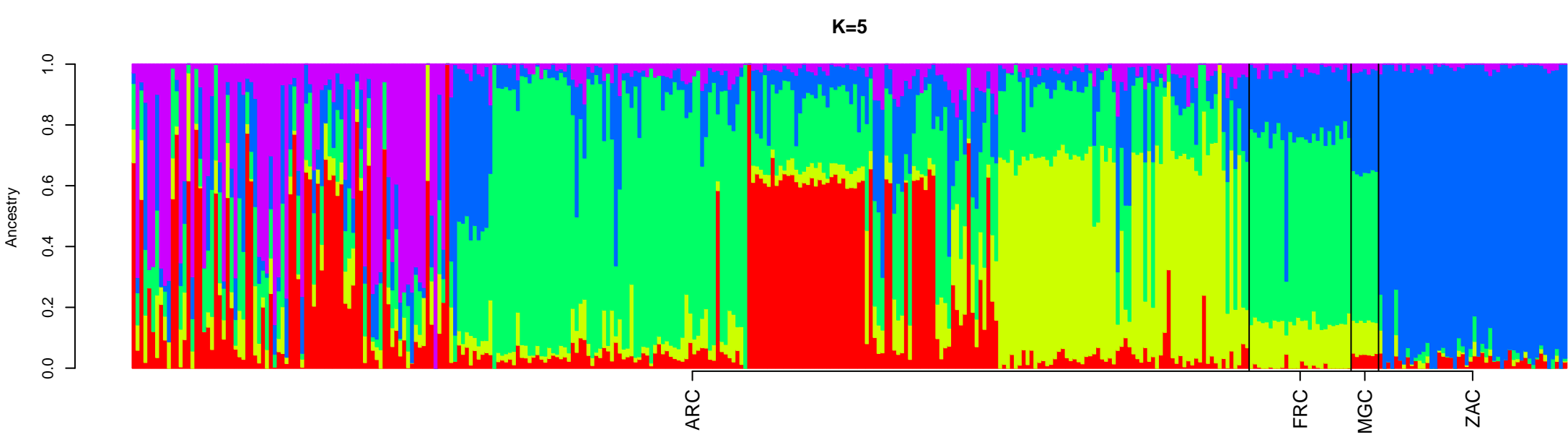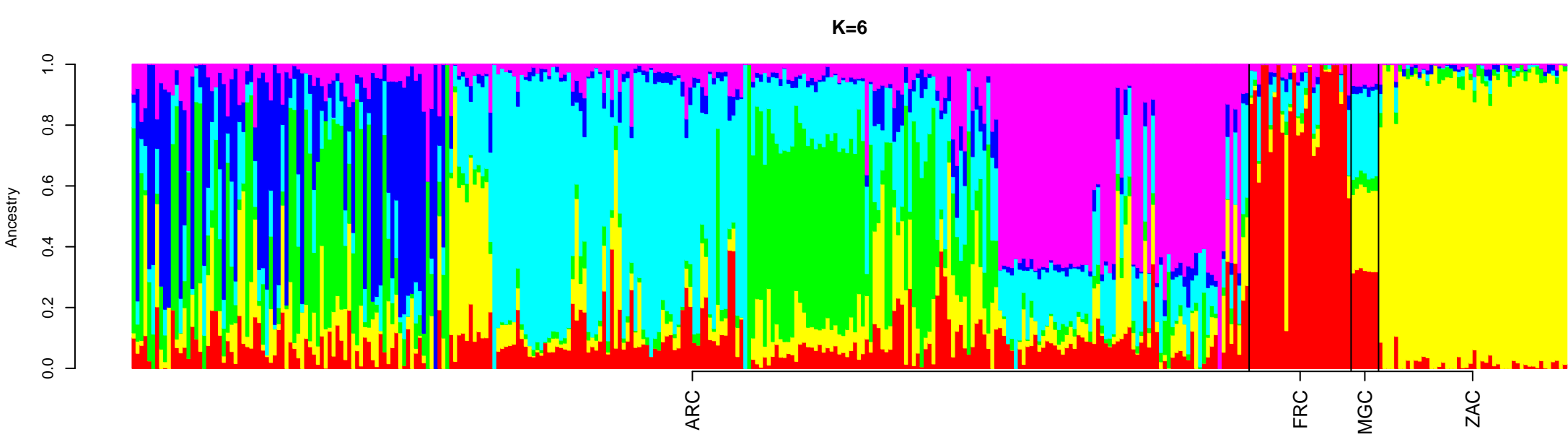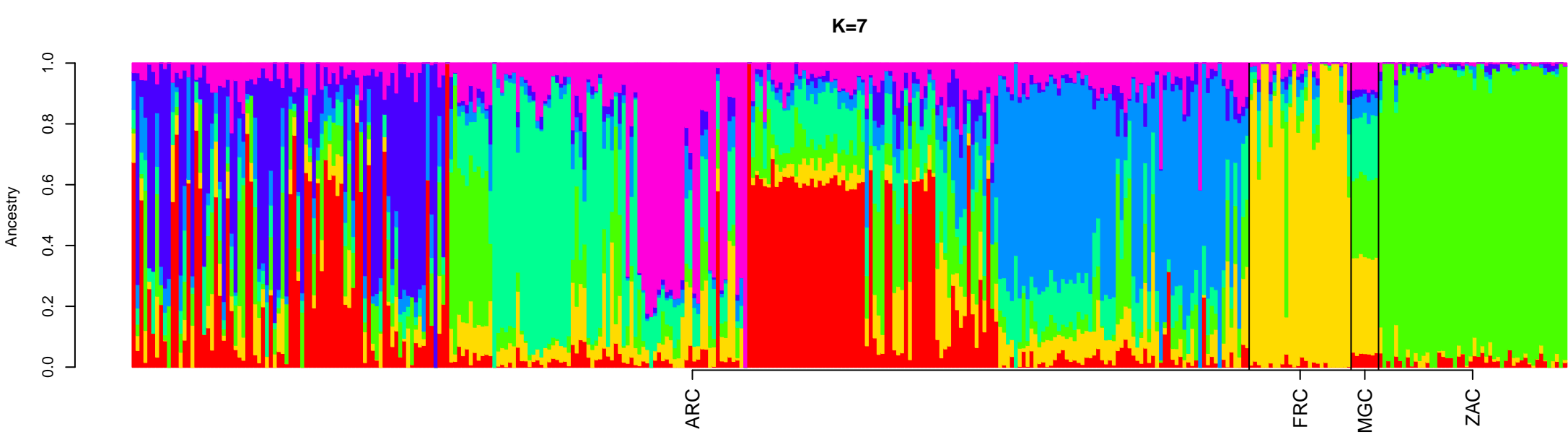

Supplement: Supplementary file 1 — Supplementary Information 1. [file 41598_2023_27490_MOESM1_ESM.pdf]

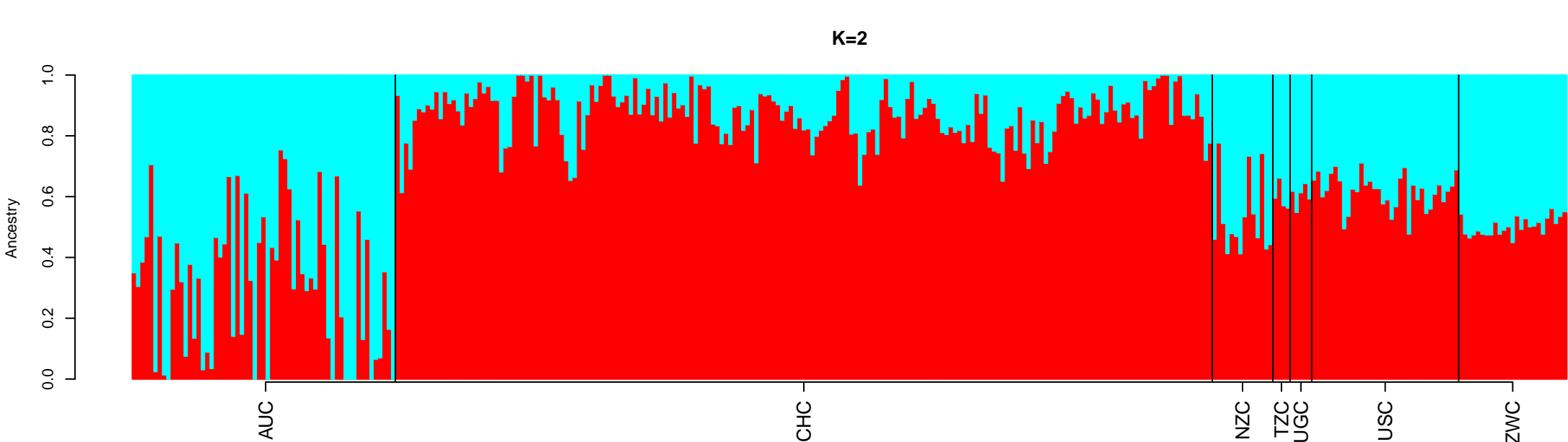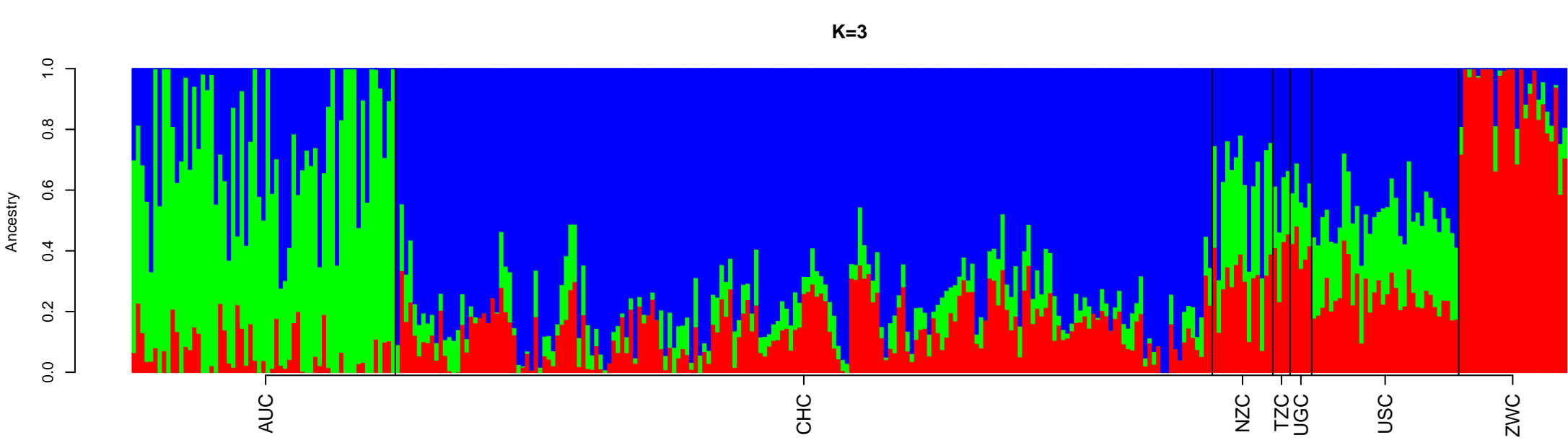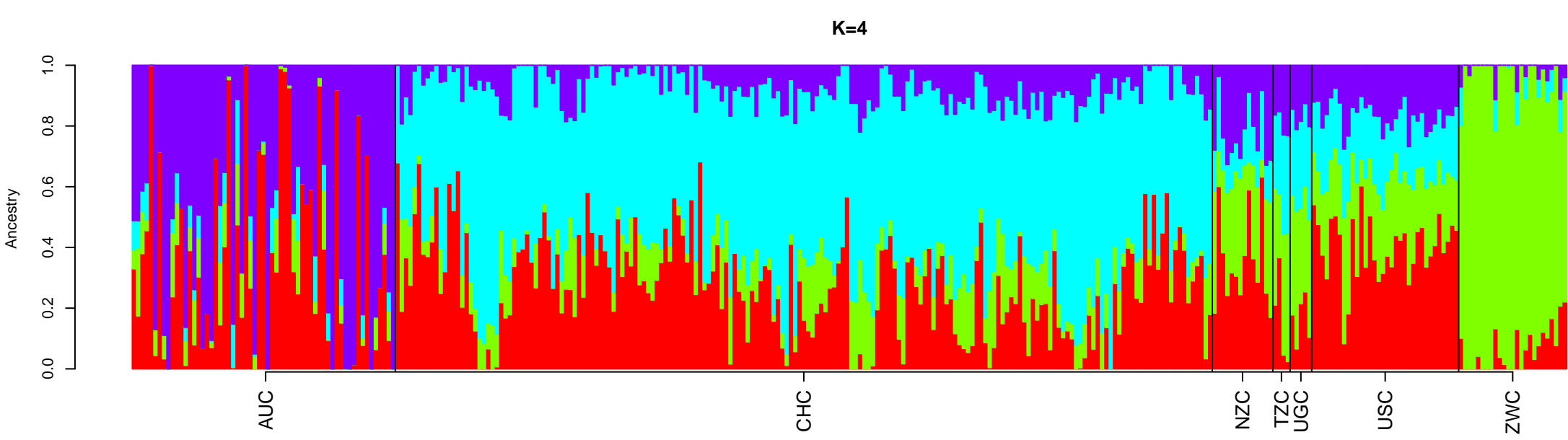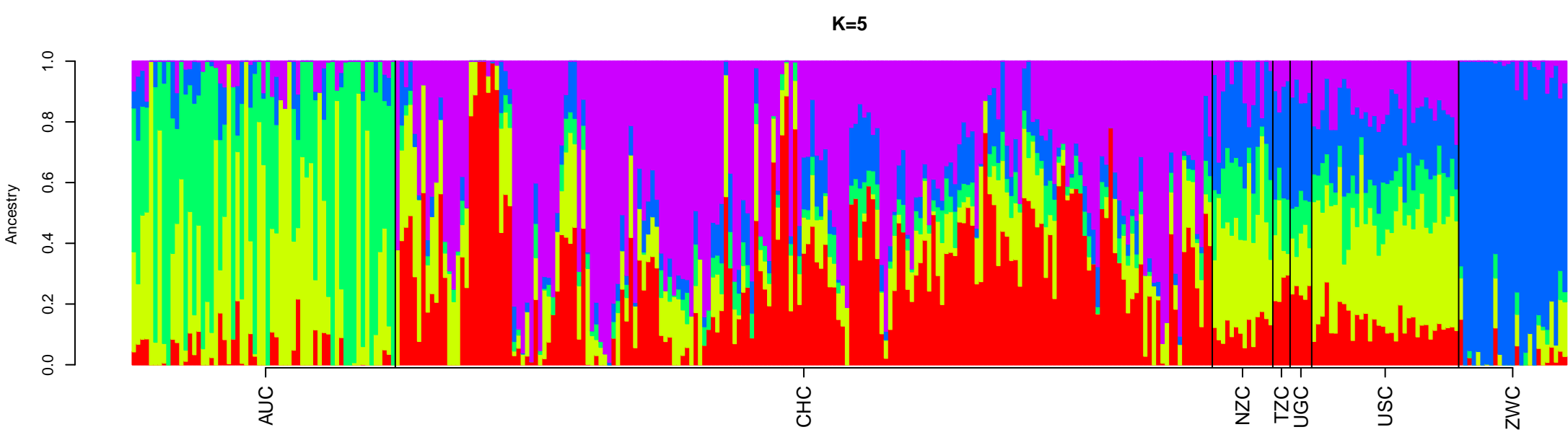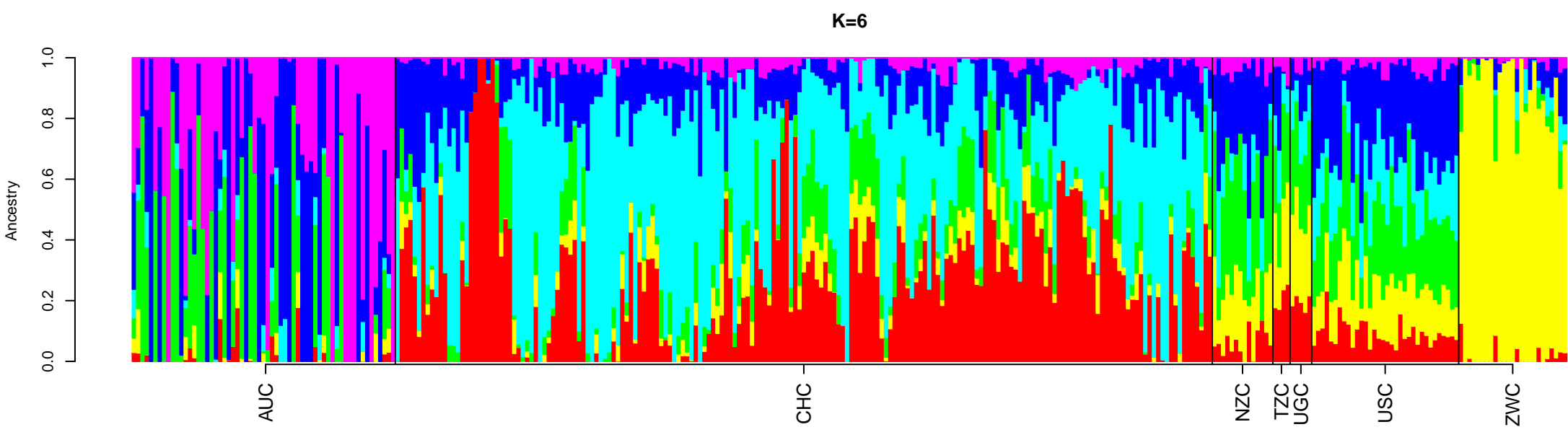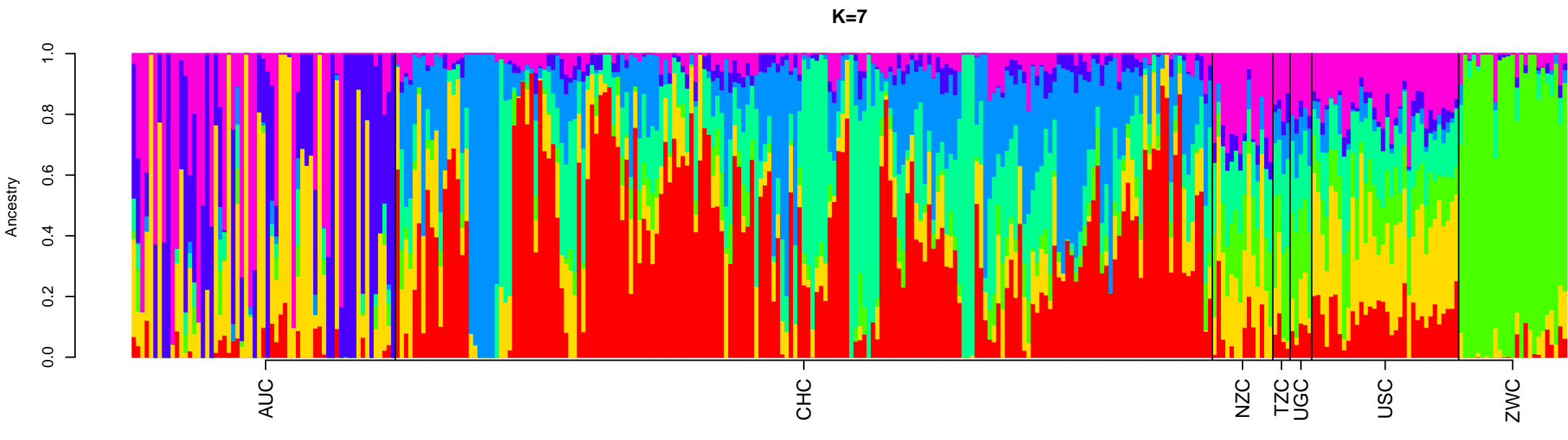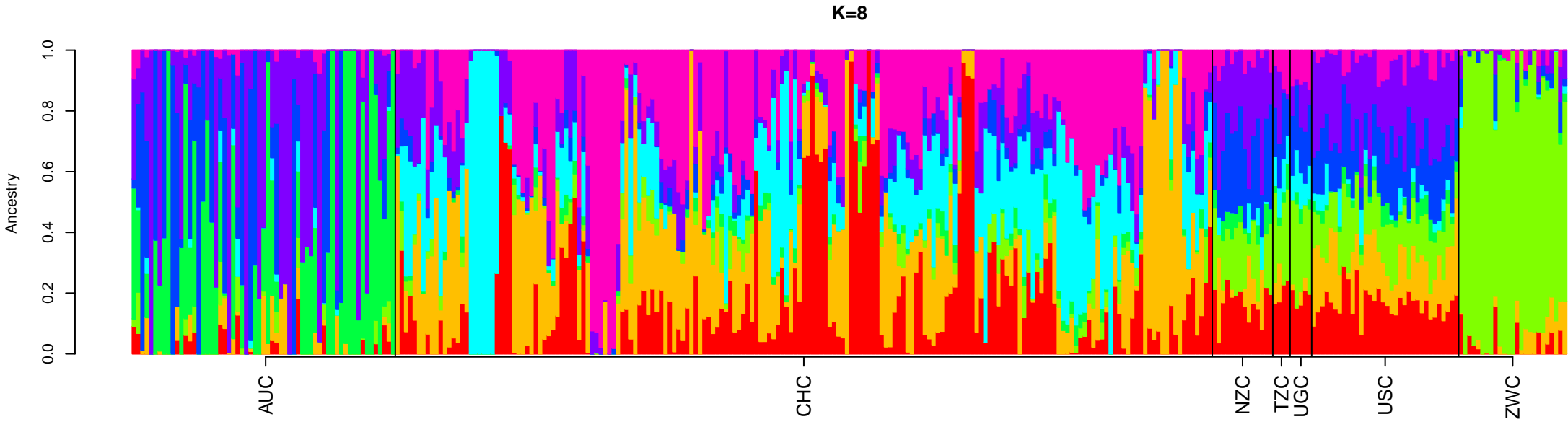

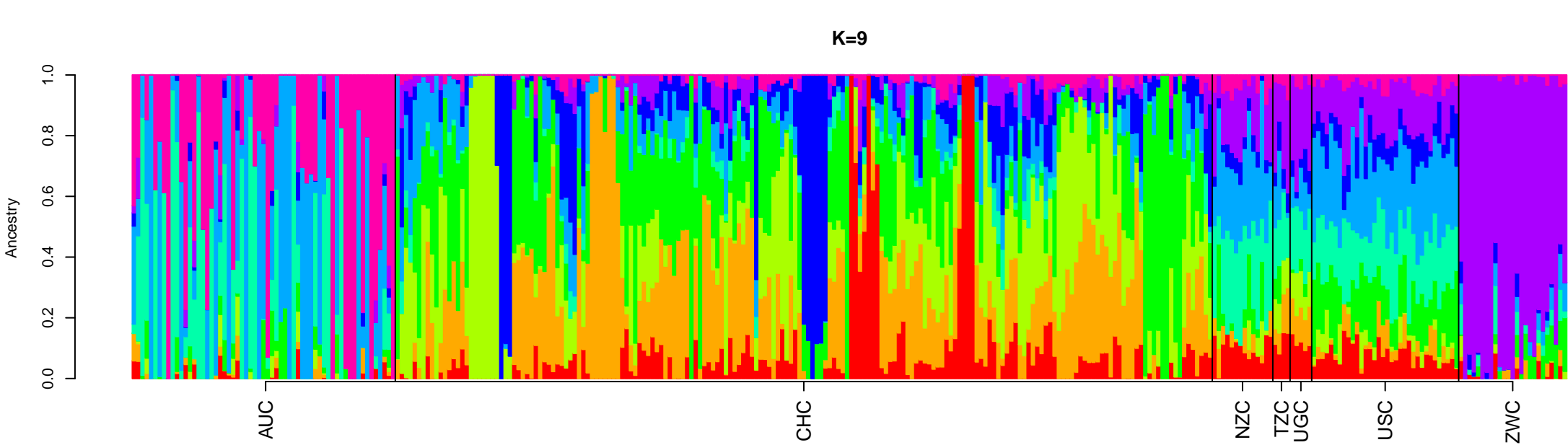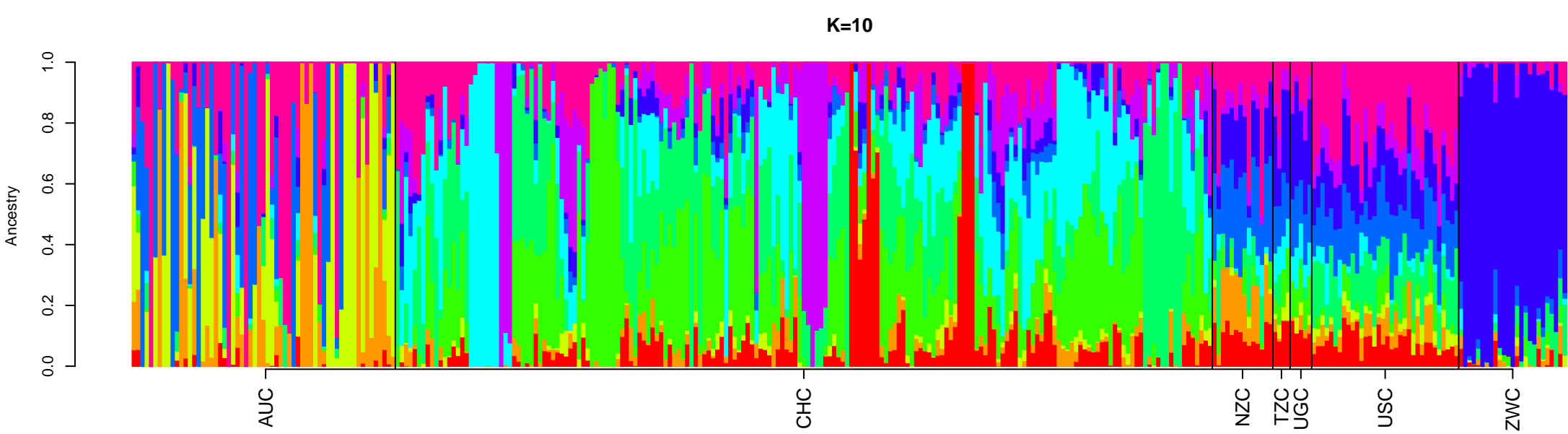

Supplement: Supplementary file 2 — Supplementary Information 2. [file 41598_2023_27490_MOESM2_ESM.pdf]

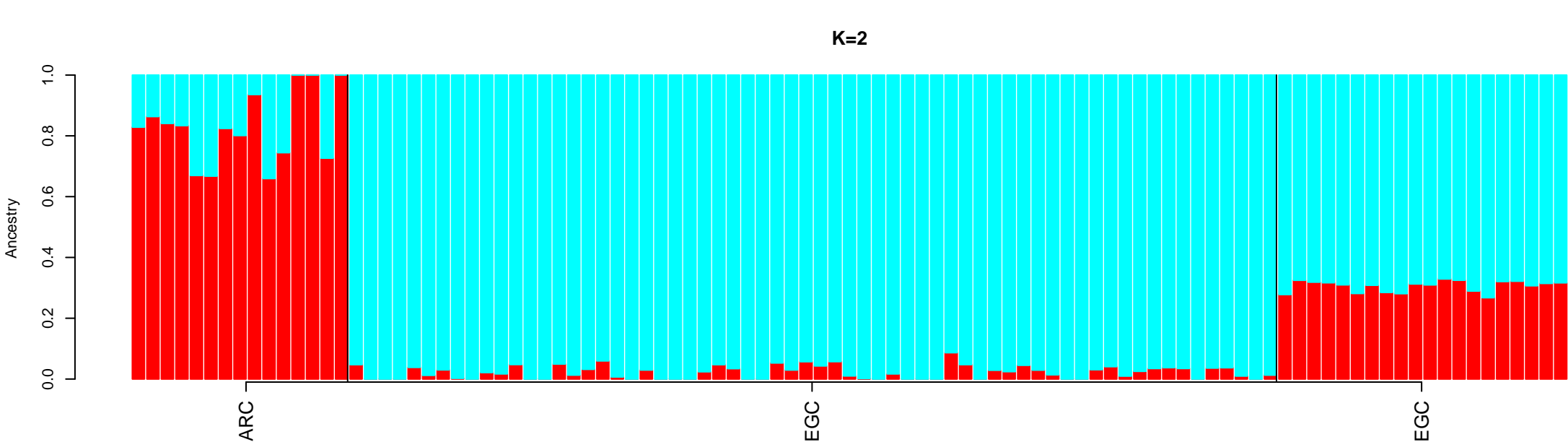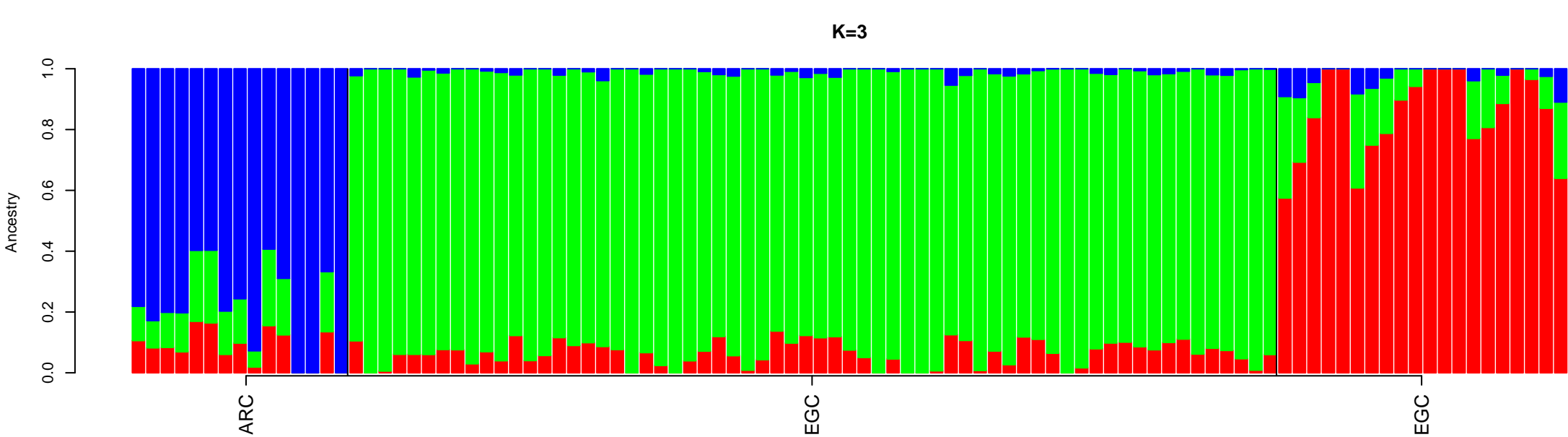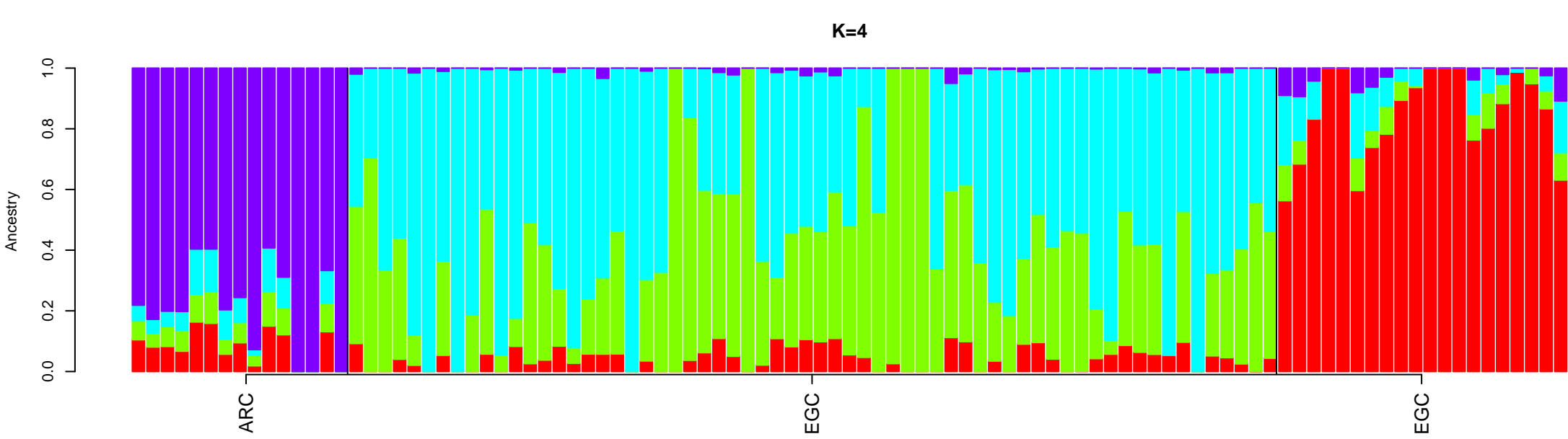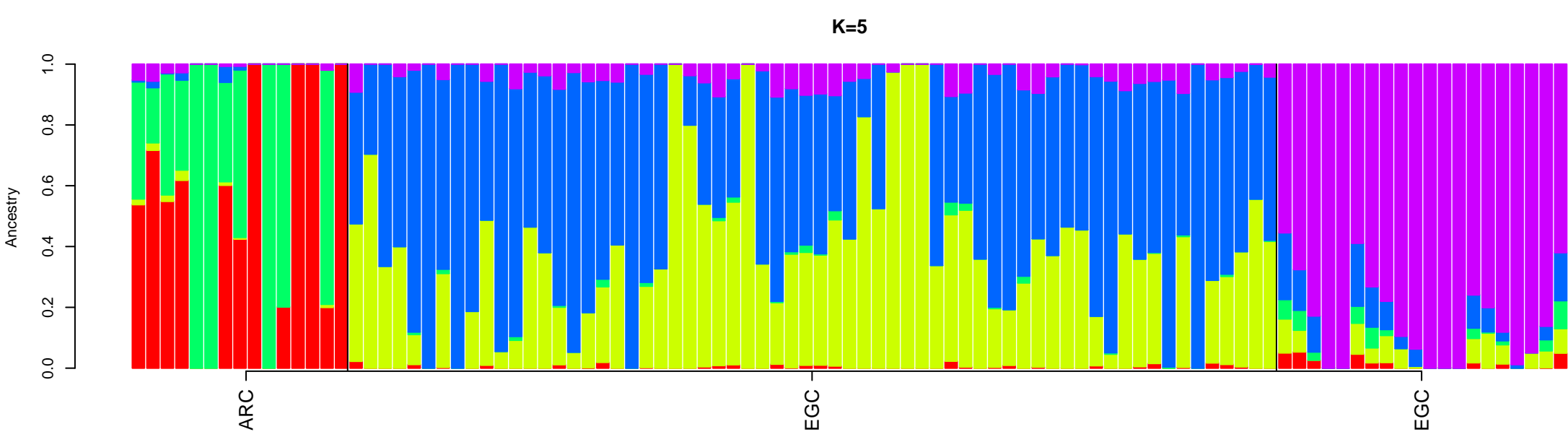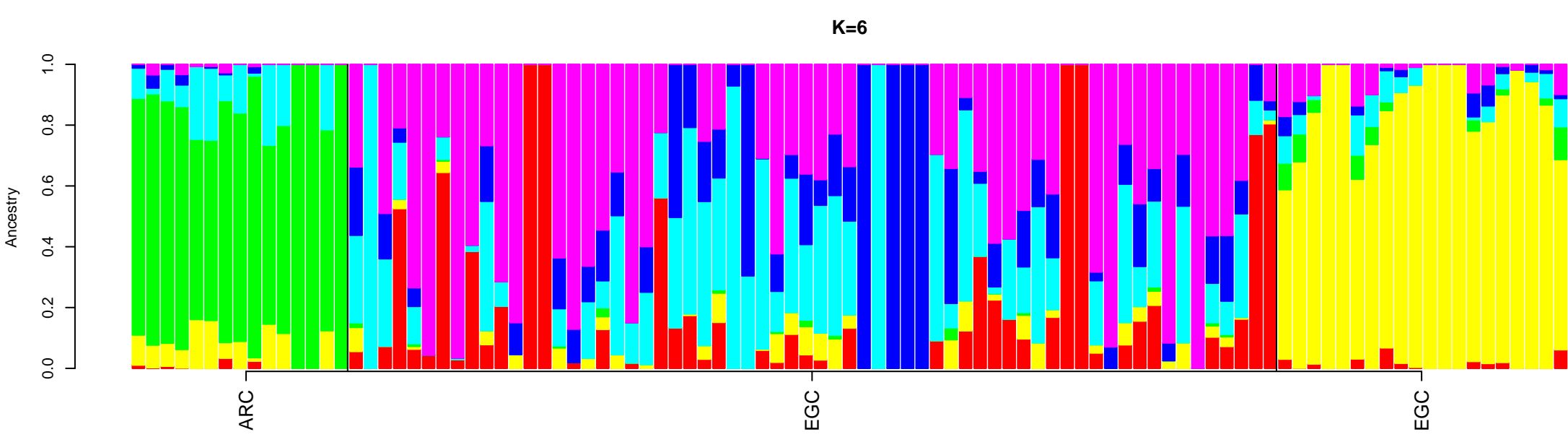

Supplement: Supplementary file 3 — Supplementary Information 3. [file 41598_2023_27490_MOESM3_ESM.pdf]

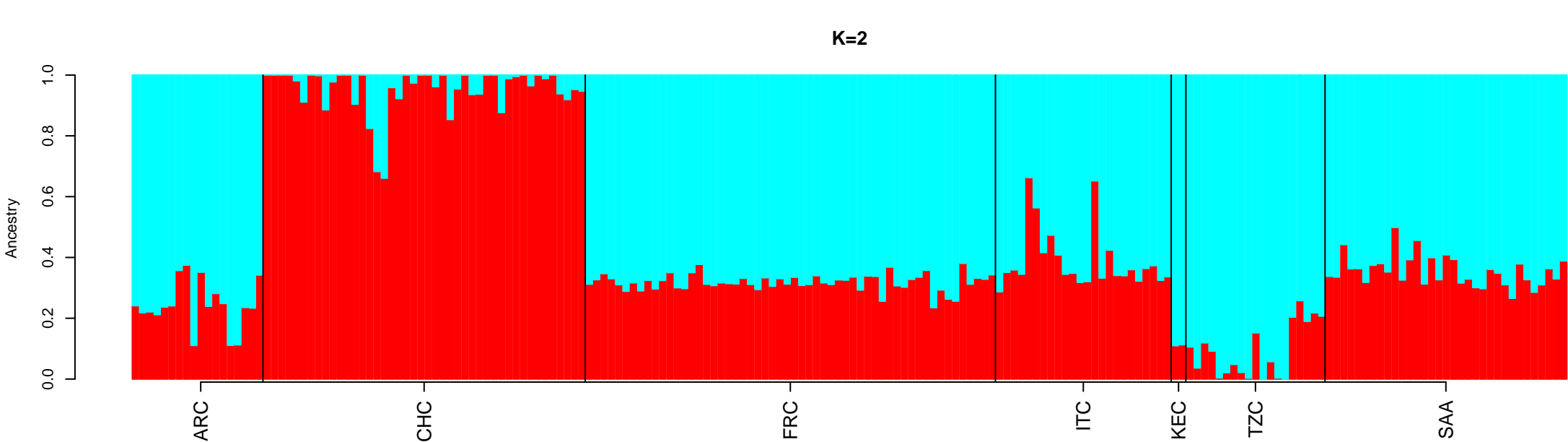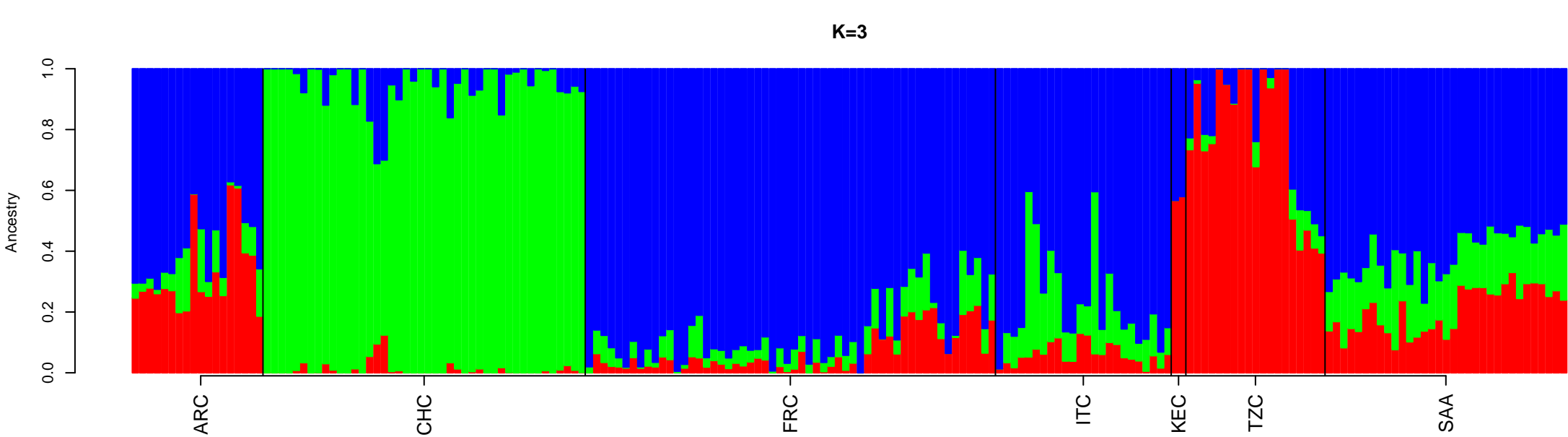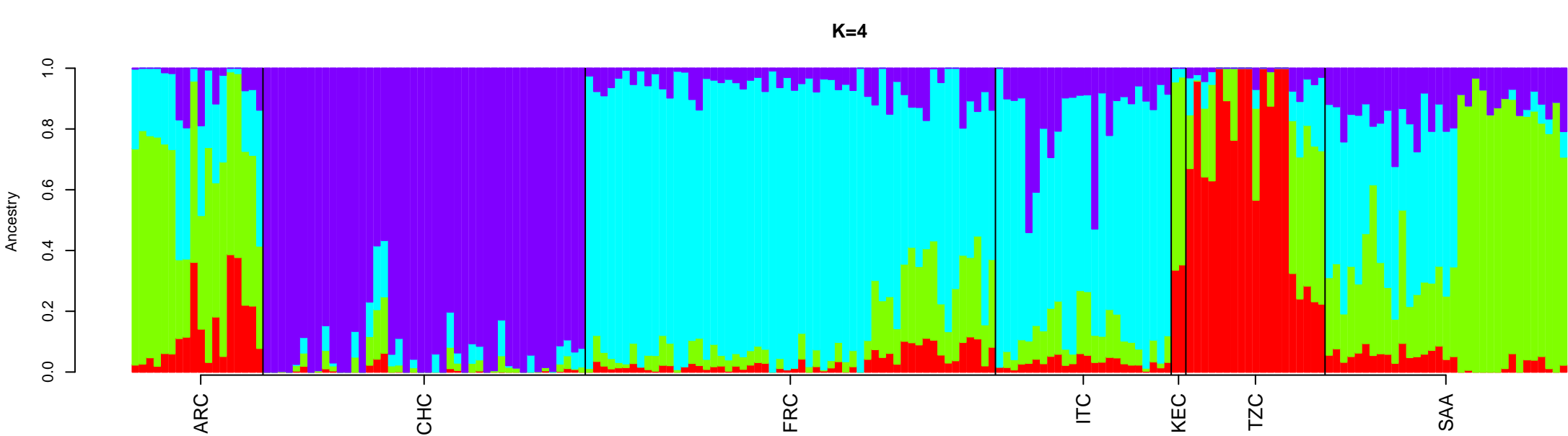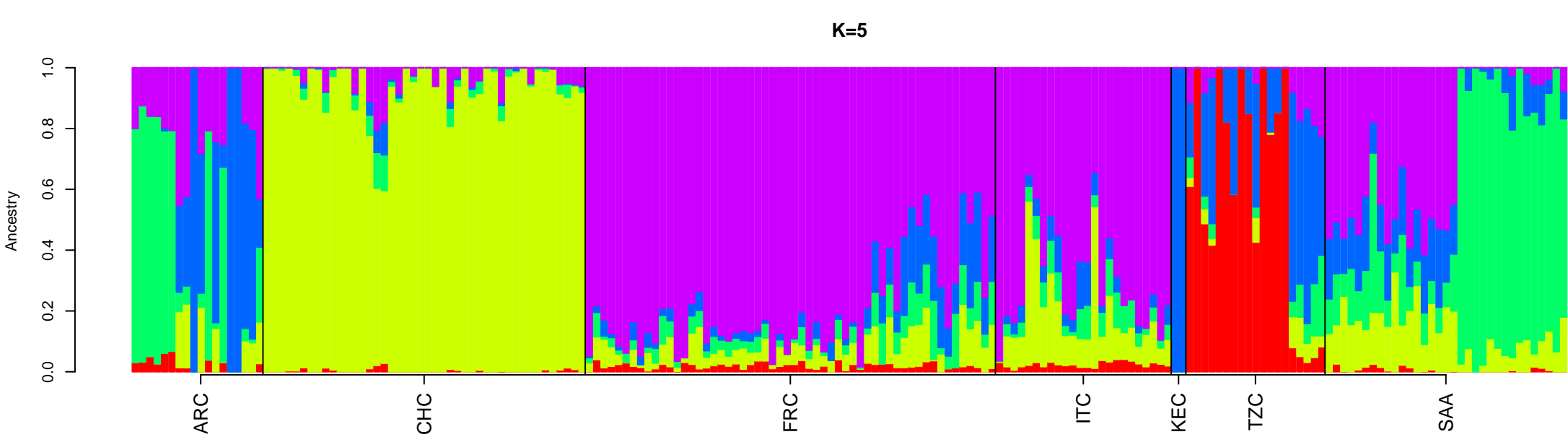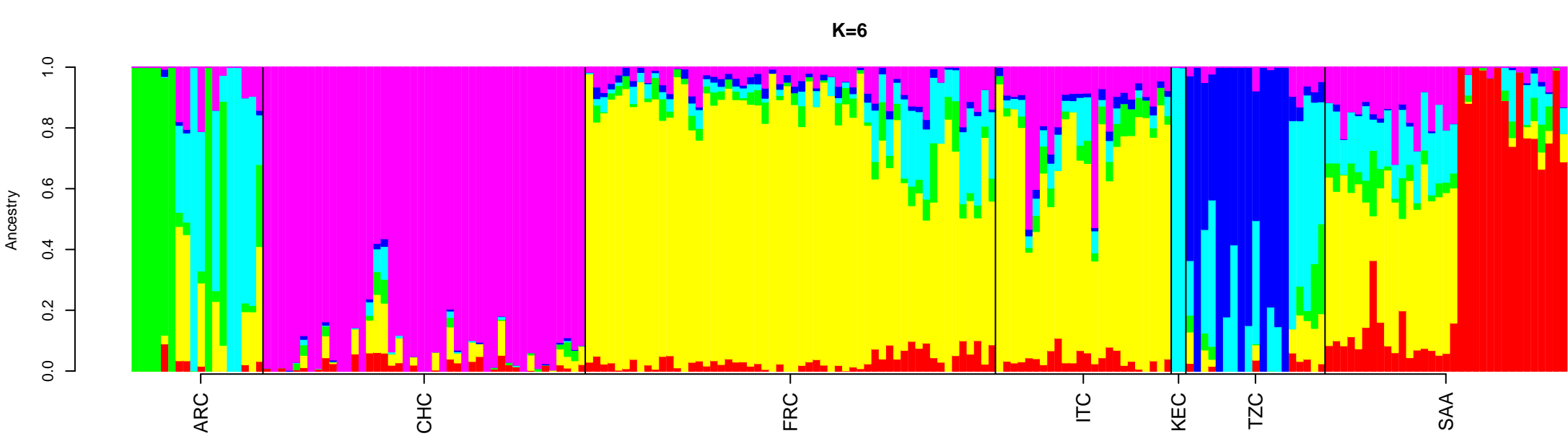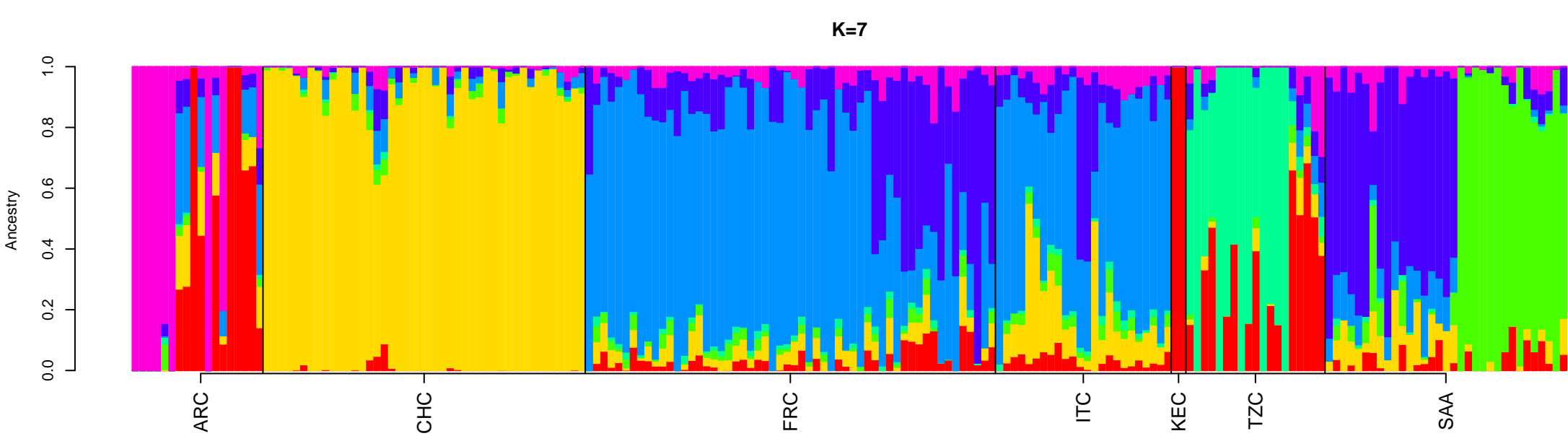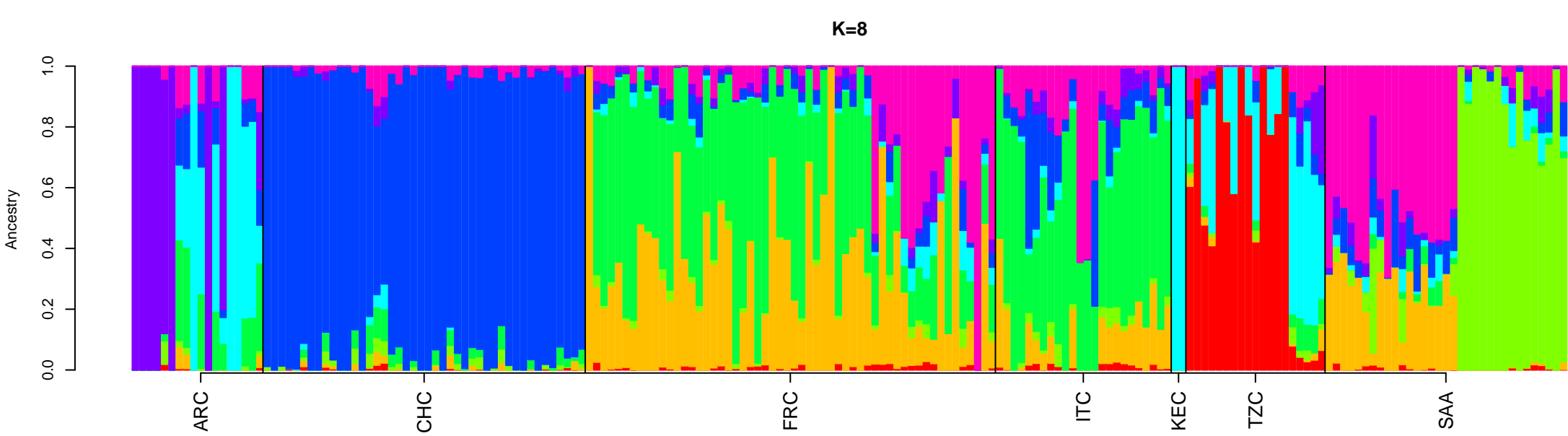

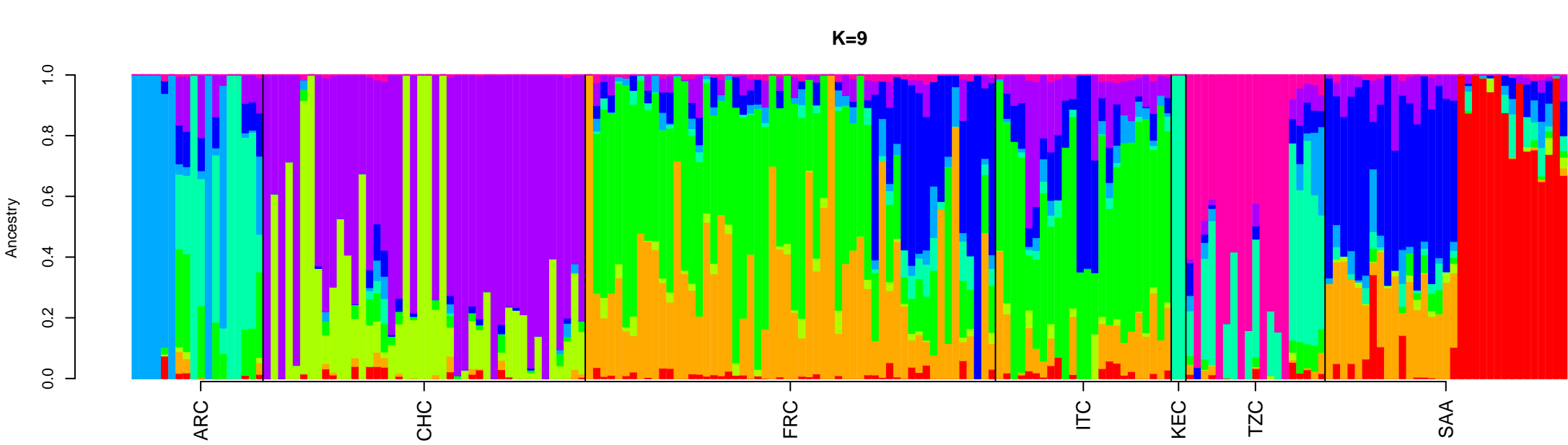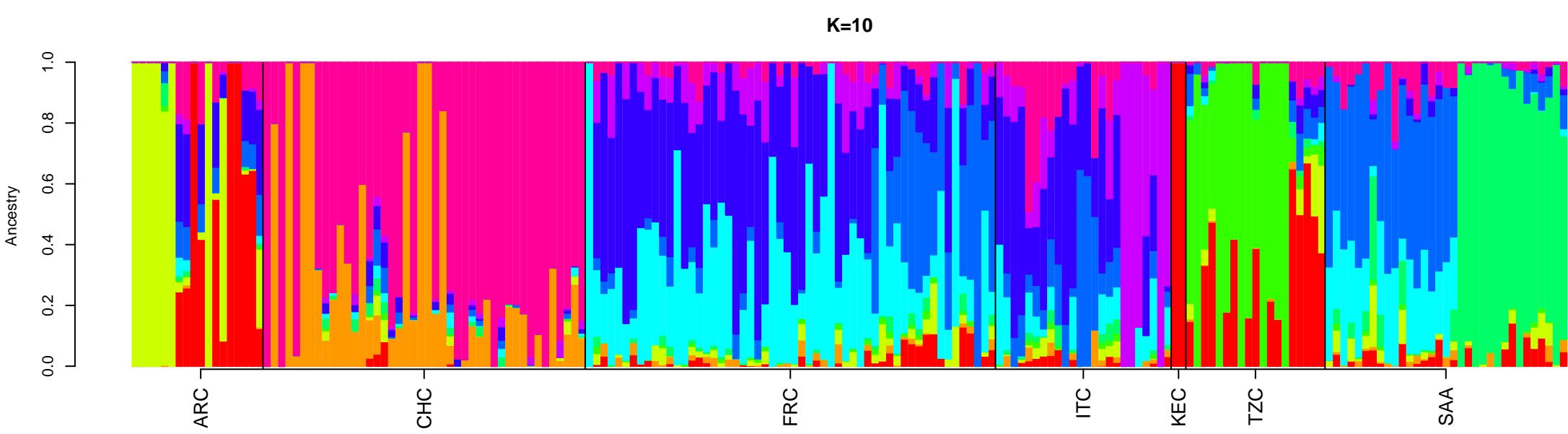

Supplement: Supplementary file 4 — Supplementary Information 4. [file 41598_2023_27490_MOESM4_ESM.pdf]

Supplementary Figure 5. Cross validation (CV) errors plots for each breed.

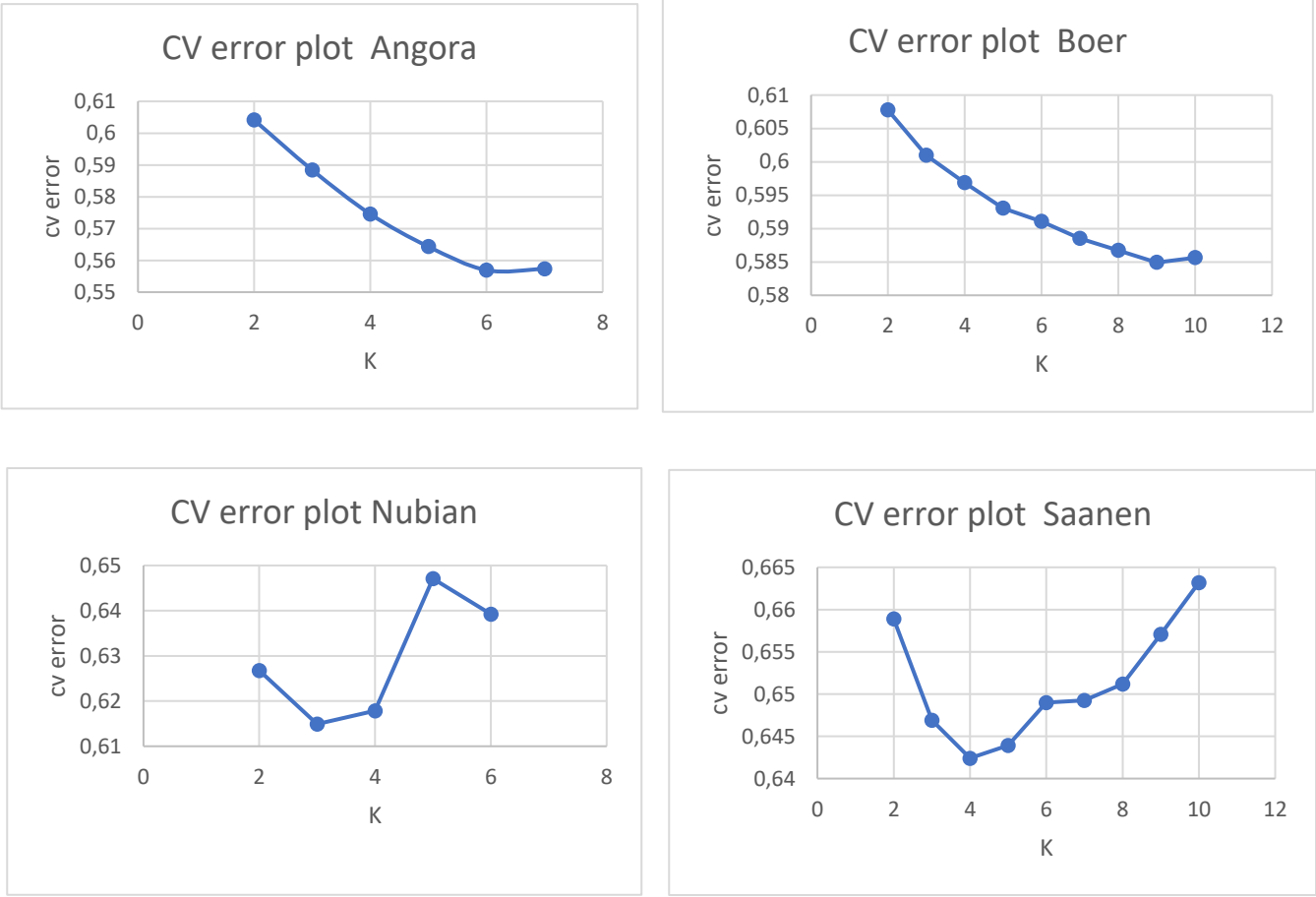

Supplement: Supplementary file 5 — Supplementary Information 5. [file 41598_2023_27490_MOESM5_ESM.pdf]
