# Supplementary material for: Discovering novel clues of natural selection on four worldwide goat breeds
Source: Sci Rep. 2023 Feb 6;13:2110. doi: 10.1038/s41598-023-27490-x (PMC9902602; doi:10.1038/s41598-023-27490-x)
Supplement: Supplementary file 6 — Supplementary Information 6. [file 41598_2023_27490_MOESM6_ESM.pdf]

Supplementary Figure 6

Scree plots for a) Angora, b) Boer, c) Nubian and d) Saanen breeds.

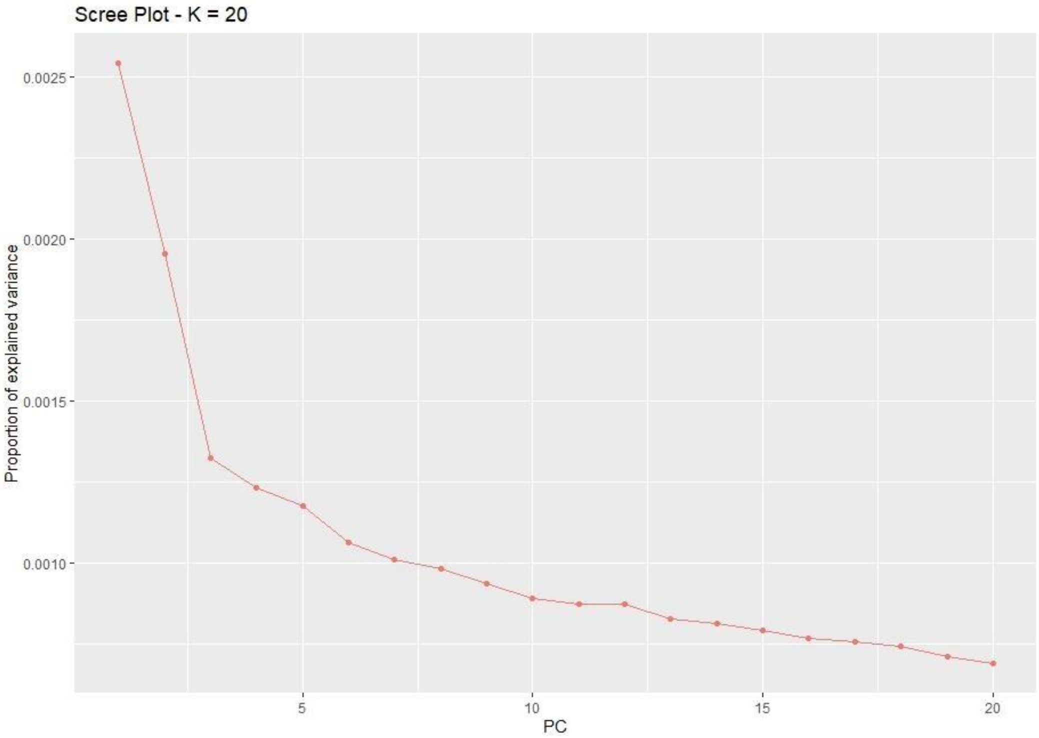

a)

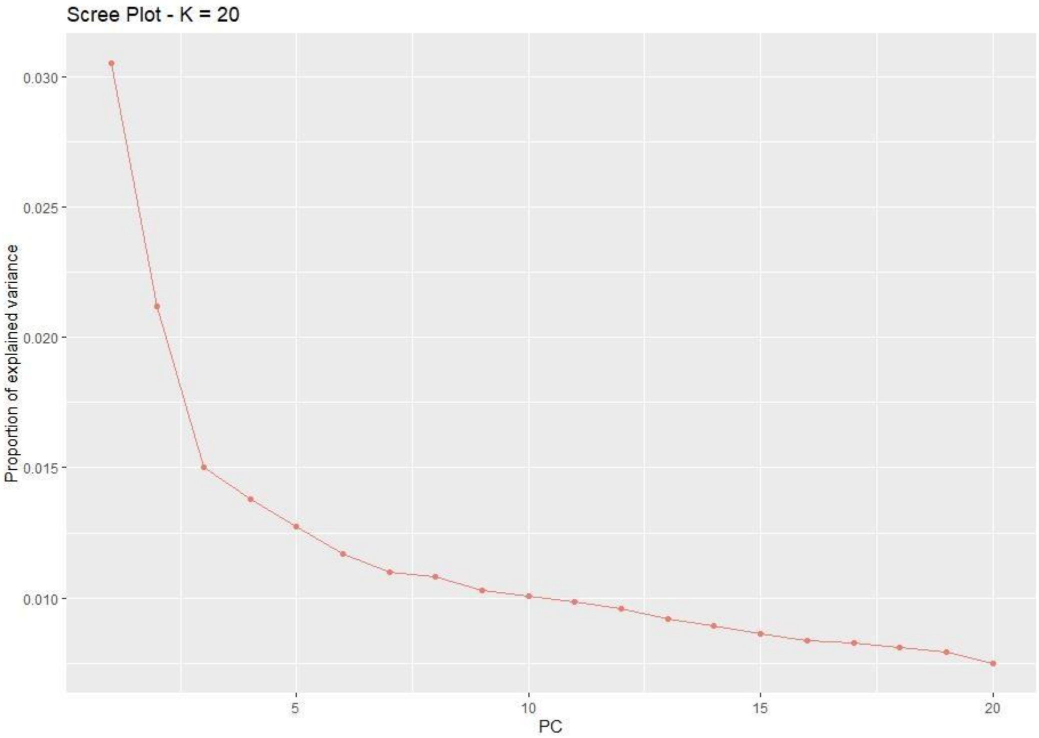

b)

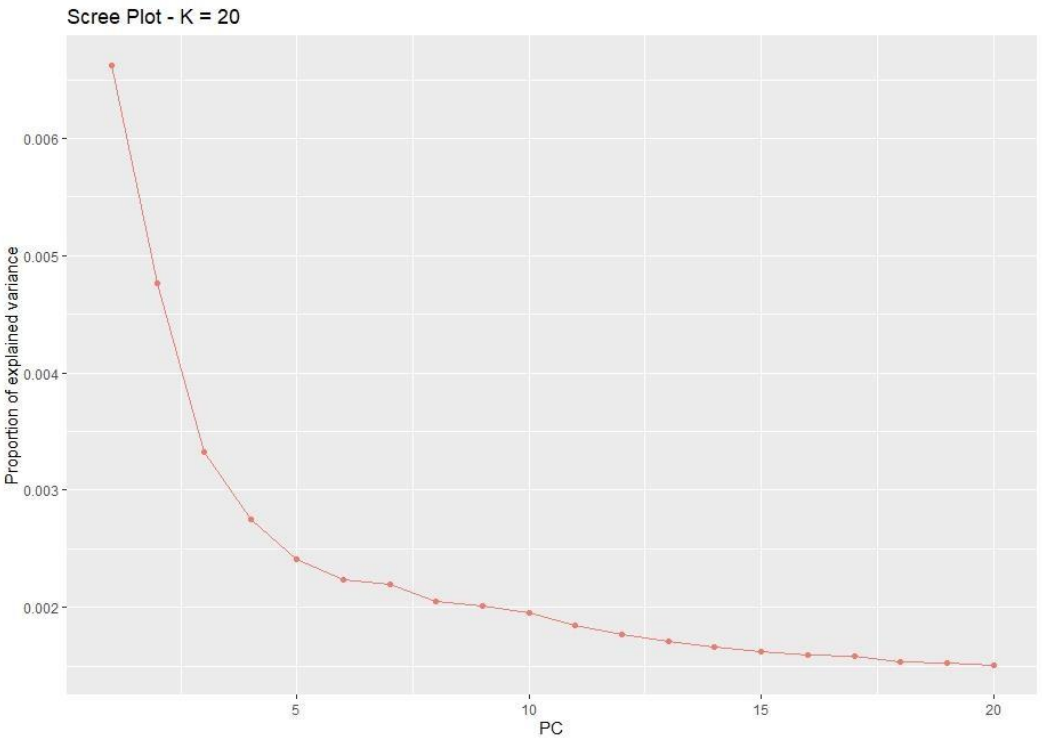
Scree Plot - K = 20
Proportion of explained variance
0.006
0.005
0.004
0.003
0.002
PC
5
10
15
20

c)

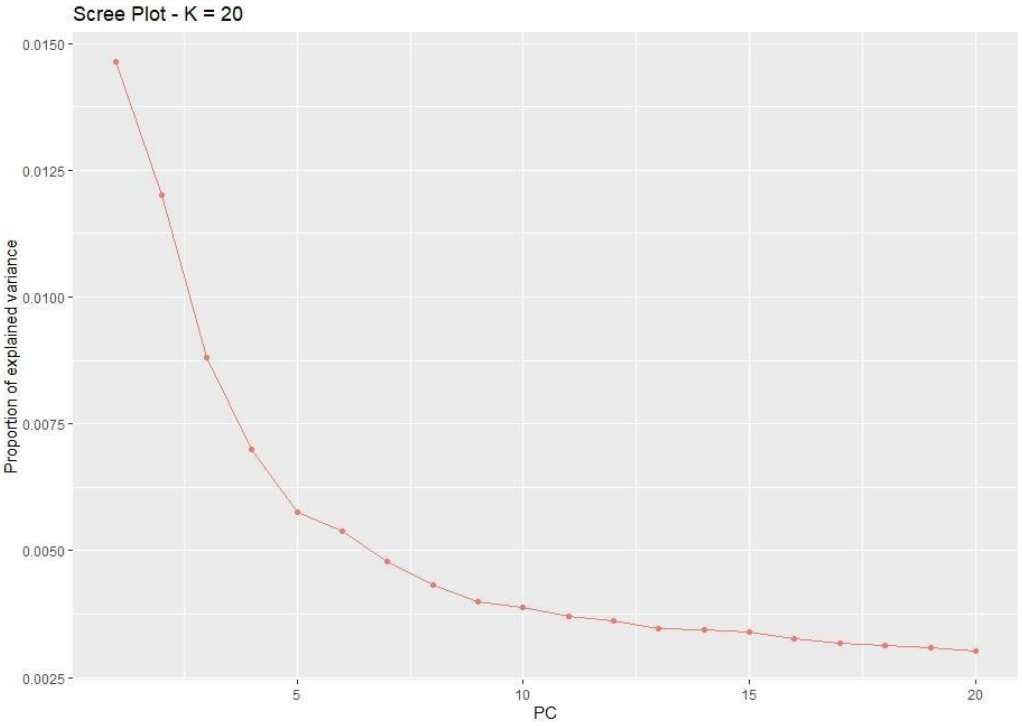
Scree Plot - K = 20
Proportion of explained variance
0.0150
0.0125
0.0100
0.0075
0.0050
0.0025
PC
5
10
15
20

d)

Supplementary Figure 7. Manhattan plots generated by detectRUNS analysis for each population belonging to the Angora goat breed. Each point corresponds to a SNP. Chromosomes are on the x-axis and the number of SNP inside the ROH is indicated on the y-axis.

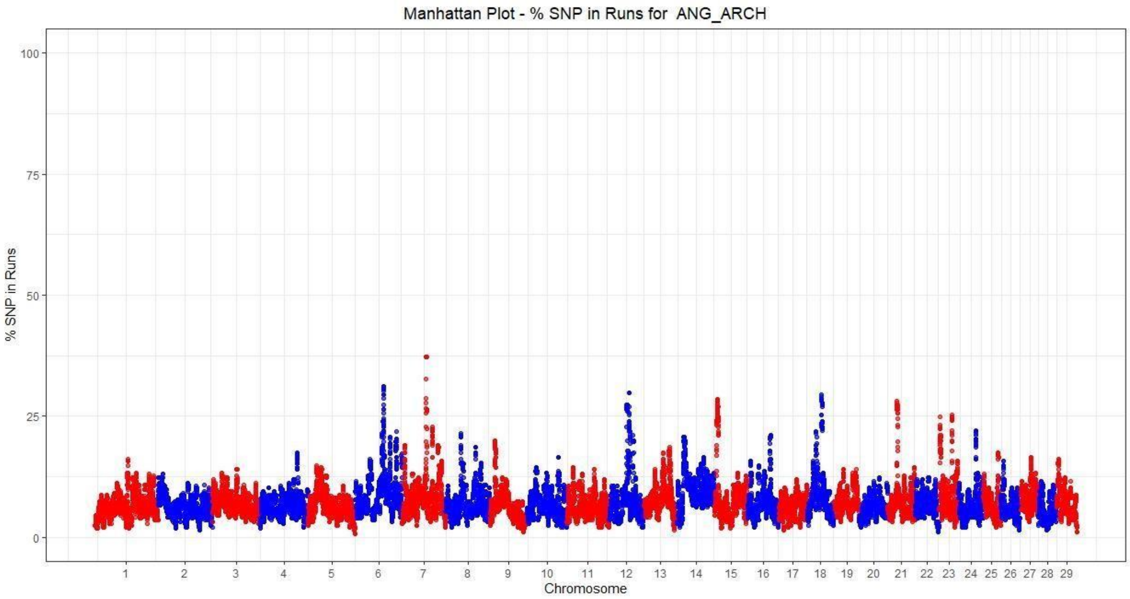

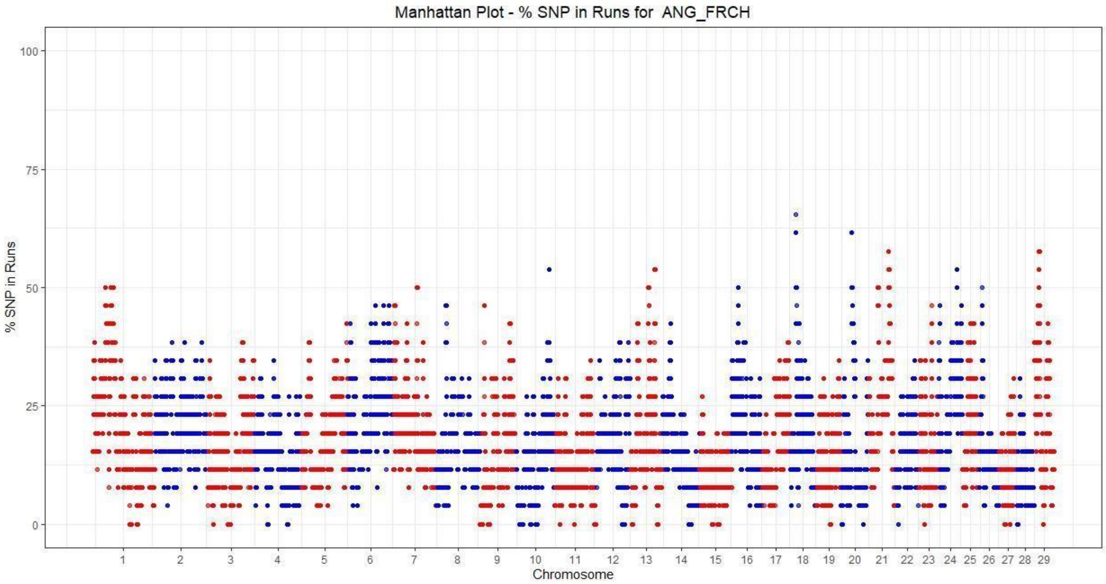

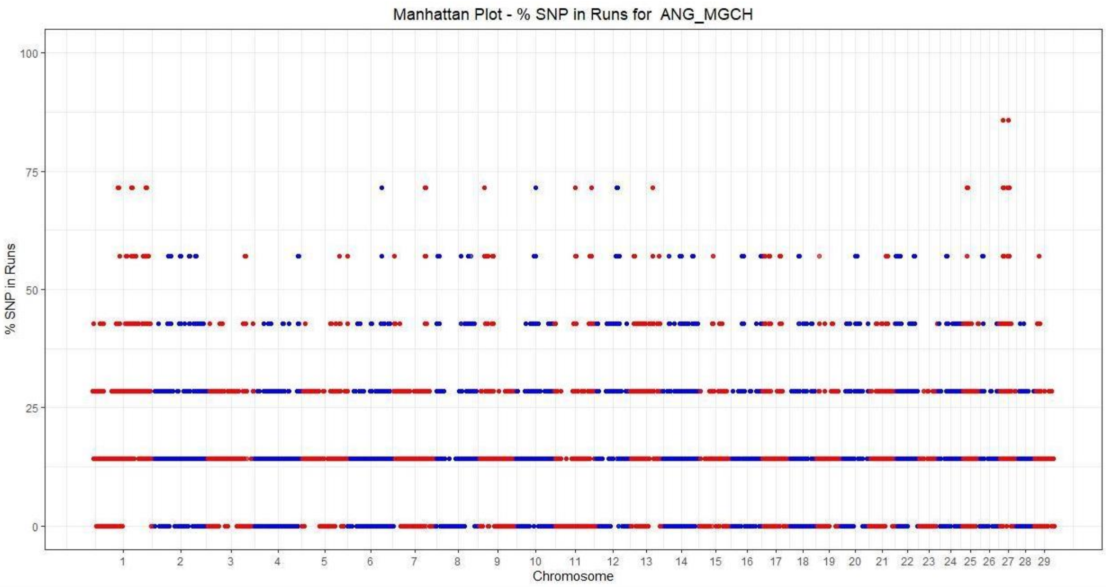
Manhattan Plot - % SNP in Runs for ANG_MGCH
% SNP in Runs
100
75
50
25
0
1 2 3 4 5 6 7 8 9 10 11 12 13 14 15 16 17 18 19 20 21 22 23 24 25 26 27 28 29
Chromosome

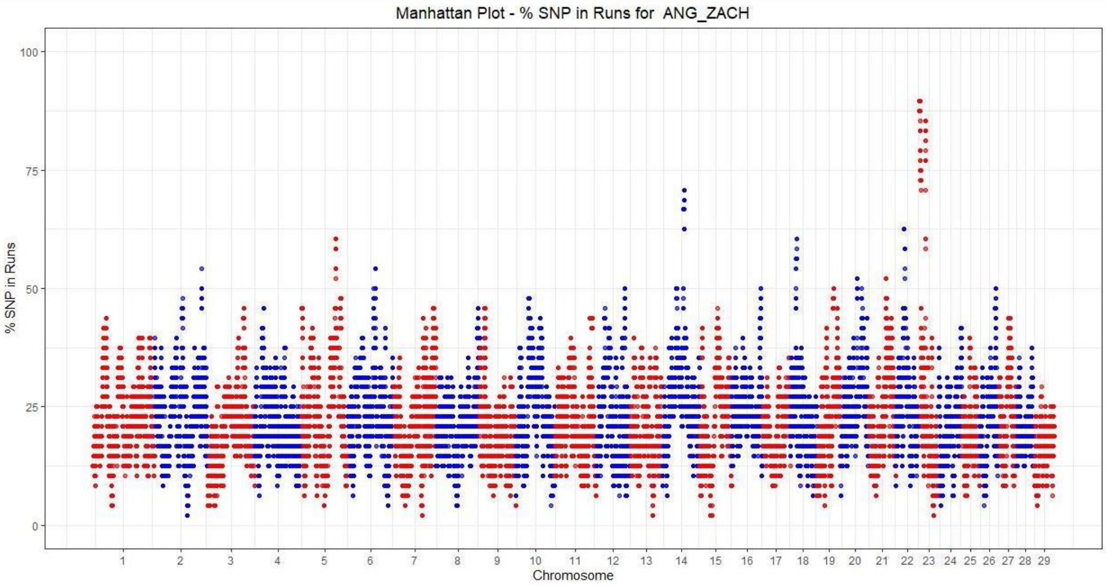
Manhattan Plot - % SNP in Runs for ANG_ZACH
% SNP in Runs
100
75
50
25
0
1 2 3 4 5 6 7 8 9 10 11 12 13 14 15 16 17 18 19 20 21 22 23 24 25 26 27 28 29
Chromosome

Supplementary Figure 8. Manhattan plots generated by detectRUNS analysis for each population belonging to the Boer goat breed. Each point corresponds to a SNP. Chromosomes are on the x-axis and the number of SNP inside the ROH is indicated on the y-axis.

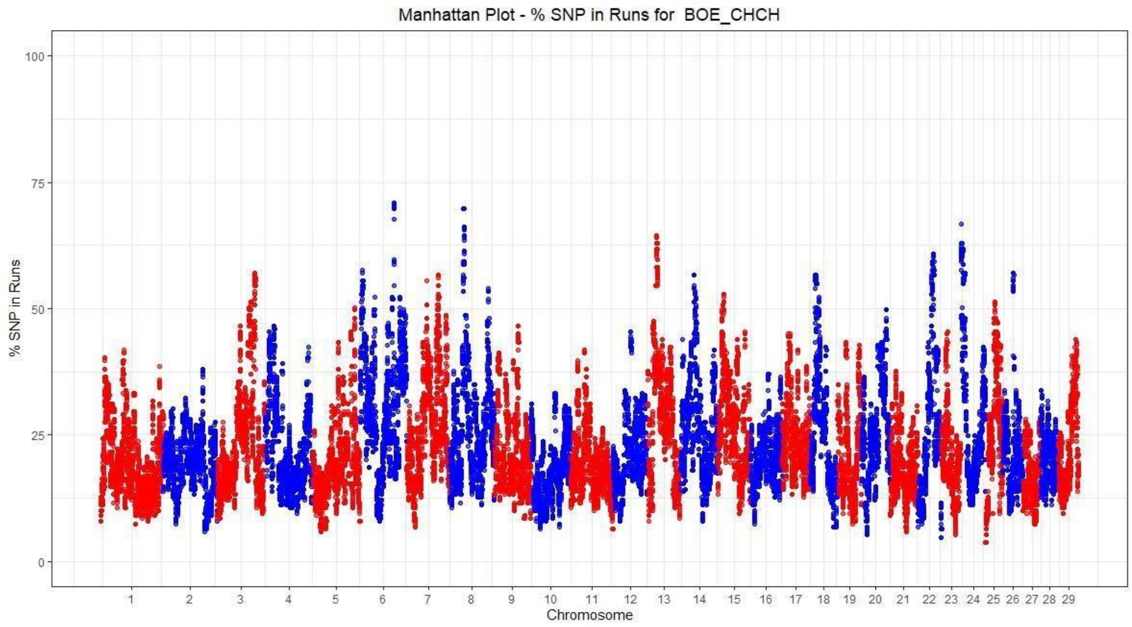

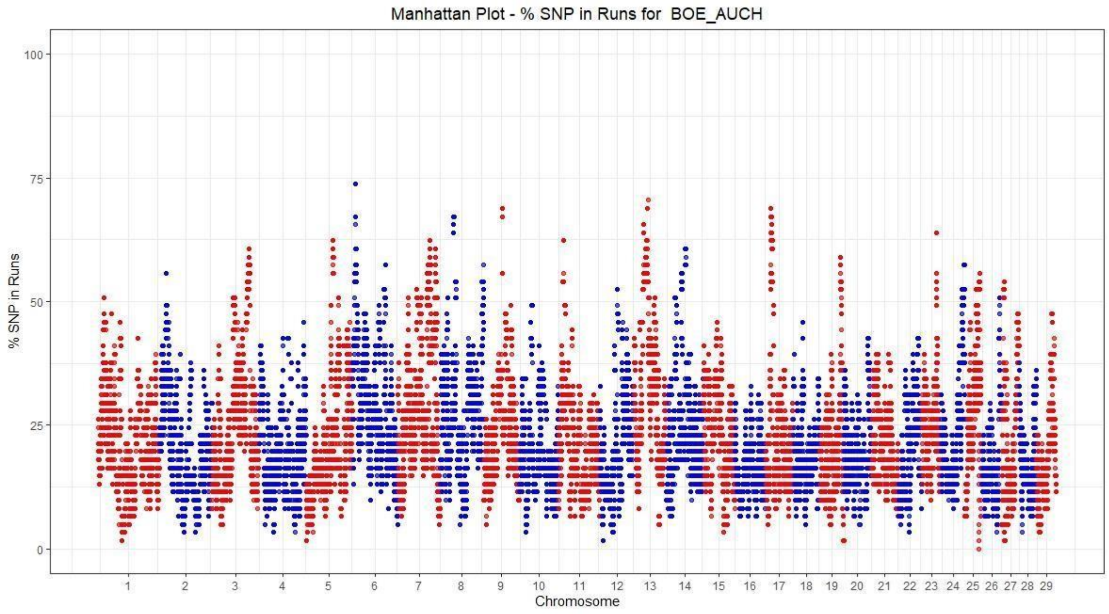

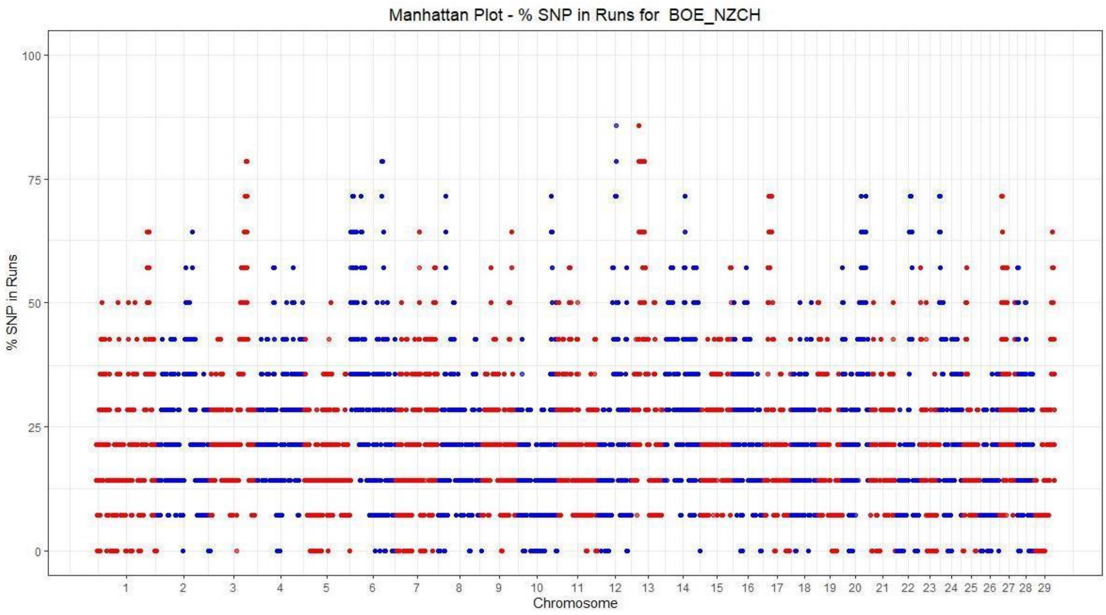
Manhattan Plot - % SNP in Runs for BOE_NZCH
% SNP in Runs
Chromosome
0
25
50
75
100
1 2 3 4 5 6 7 8 9 10 11 12 13 14 15 16 17 18 19 20 21 22 23 24 25 26 27 28 29

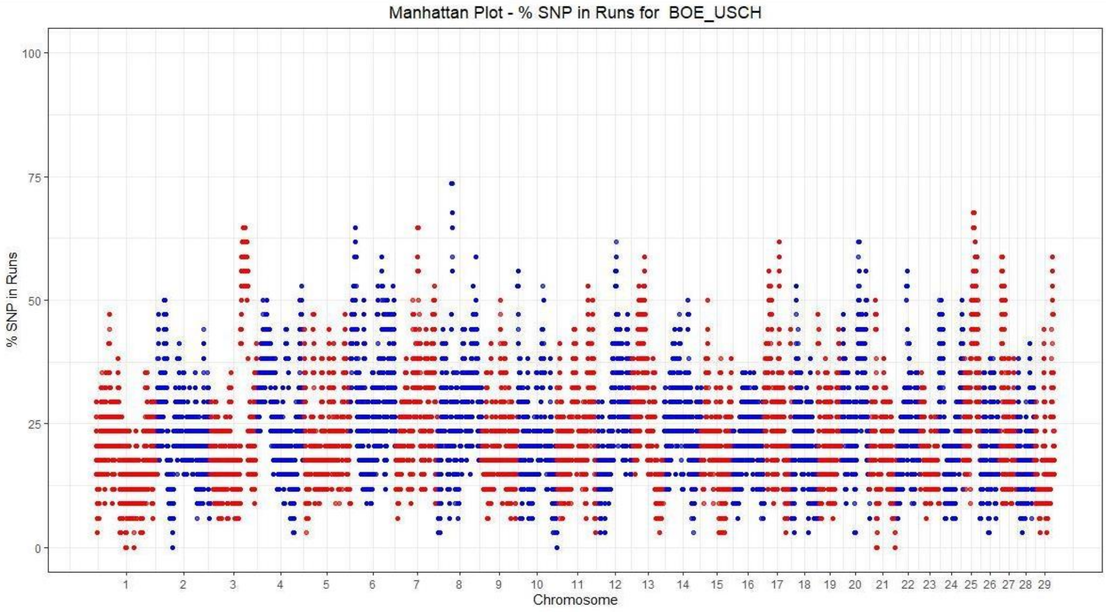
Manhattan Plot - % SNP in Runs for BOE_USCH
% SNP in Runs
Chromosome
0
25
50
75
100
1 2 3 4 5 6 7 8 9 10 11 12 13 14 15 16 17 18 19 20 21 22 23 24 25 26 27 28 29

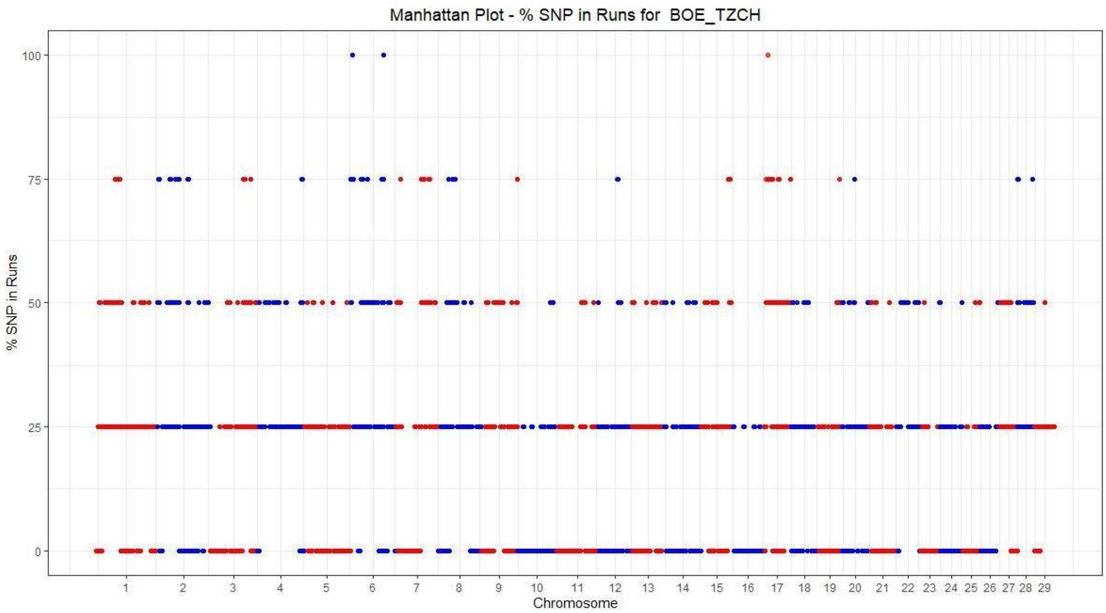

Manhattan Plot - % SNP in Runs for BOE_TZCH
% SNP in Runs
100
75
50
25
0
1 2 3 4 5 6 7 8 9 10 11 12 13 14 15 16 17 18 19 20 21 22 23 24 25 26 27 28 29
Chromosome

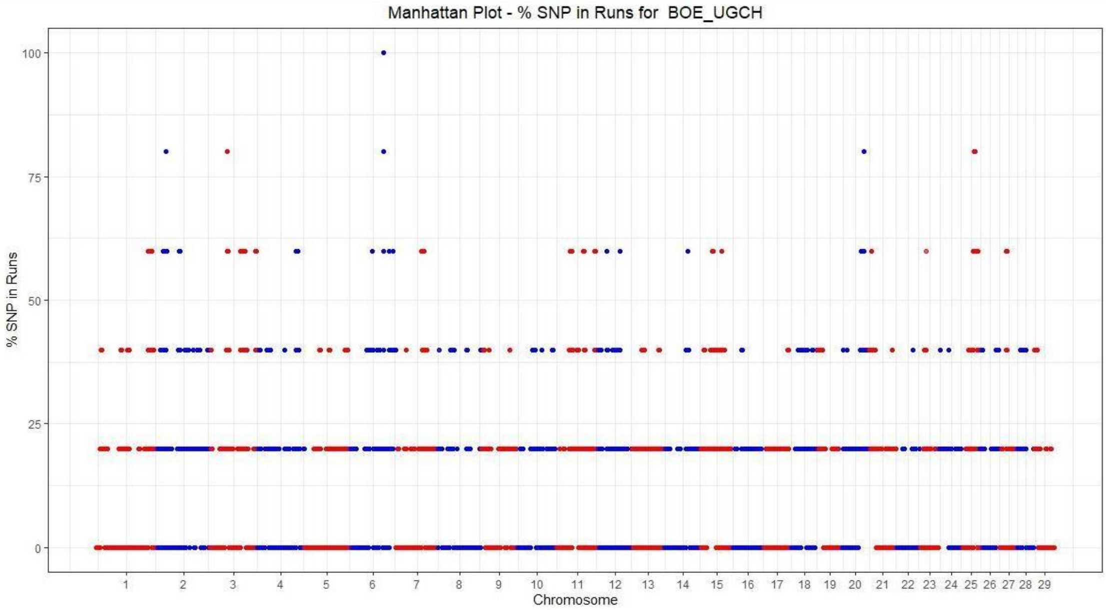

Manhattan Plot - % SNP in Runs for BOE_UGCH
% SNP in Runs
100
75
50
25
0
1 2 3 4 5 6 7 8 9 10 11 12 13 14 15 16 17 18 19 20 21 22 23 24 25 26 27 28 29
Chromosome

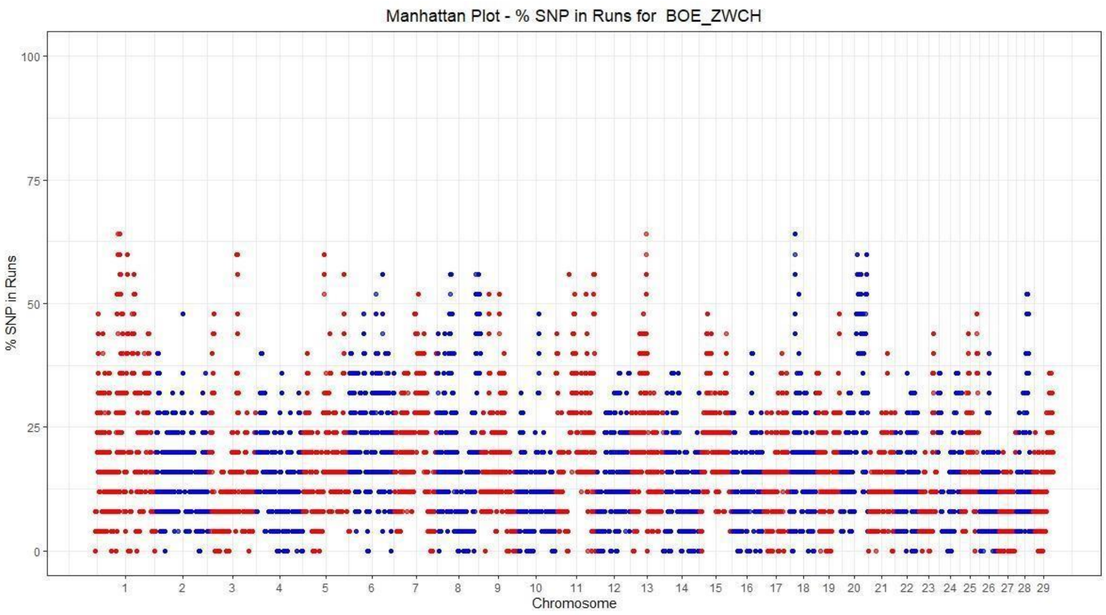

Manhattan Plot - % SNP in Runs for BOE_ZWCH
% SNP in Runs
100
75
50
25
0
1 2 3 4 5 6 7 8 9 10 11 12 13 14 15 16 17 18 19 20 21 22 23 24 25 26 27 28 29
Chromosome

Supplementary Figure 9. Manhattan plots generated by detectRUNS analysis for each population belonging to the Nubian goat breed. Each point corresponds to a SNP. Chromosomes are on the x-axis and the number of SNP inside the ROH is indicated on the y-axis.

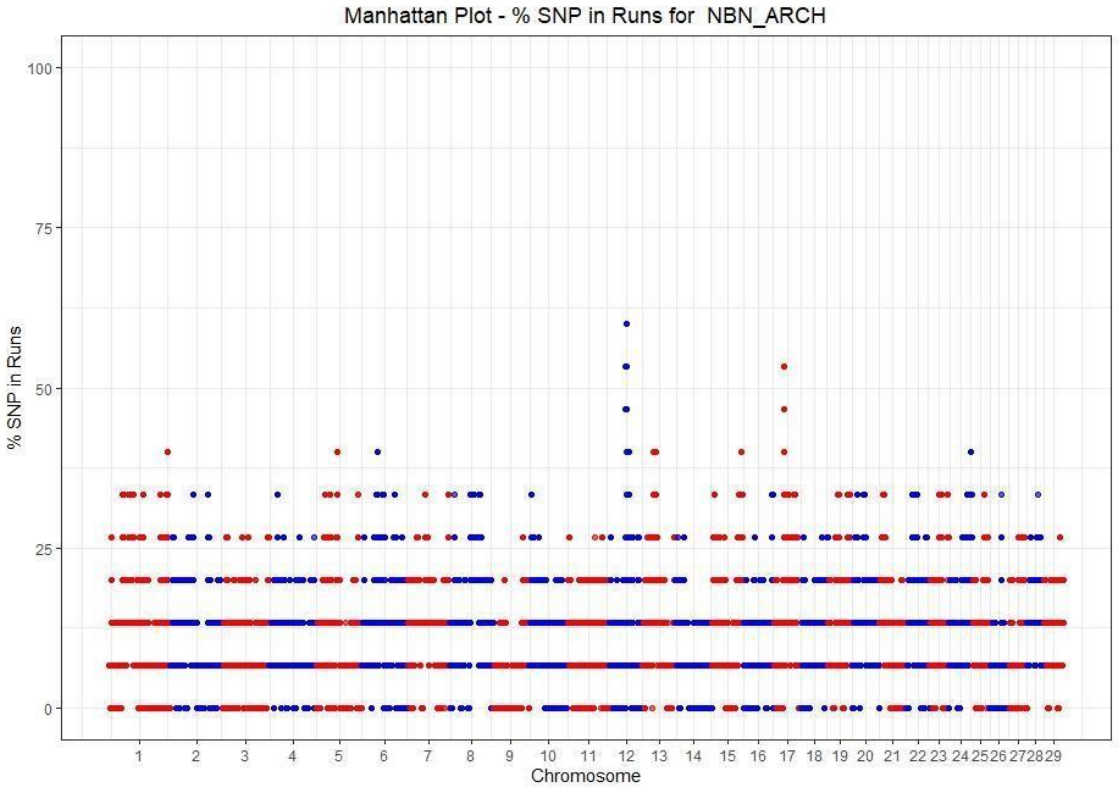

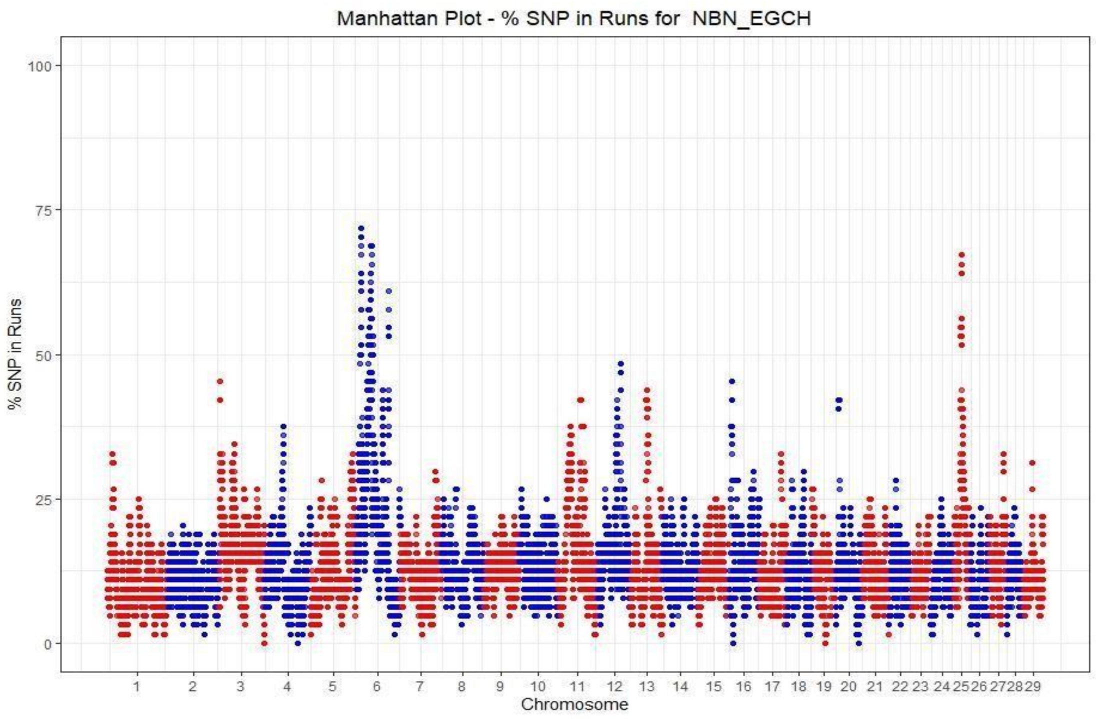
Manhattan Plot - % SNP in Runs for NBN_EGCH
% SNP in Runs
100
75
50
25
0
1 2 3 4 5 6 7 8 9 10 11 12 13 14 15 16 17 18 19 20 21 22 23 24 25 26 27 28 29
Chromosome

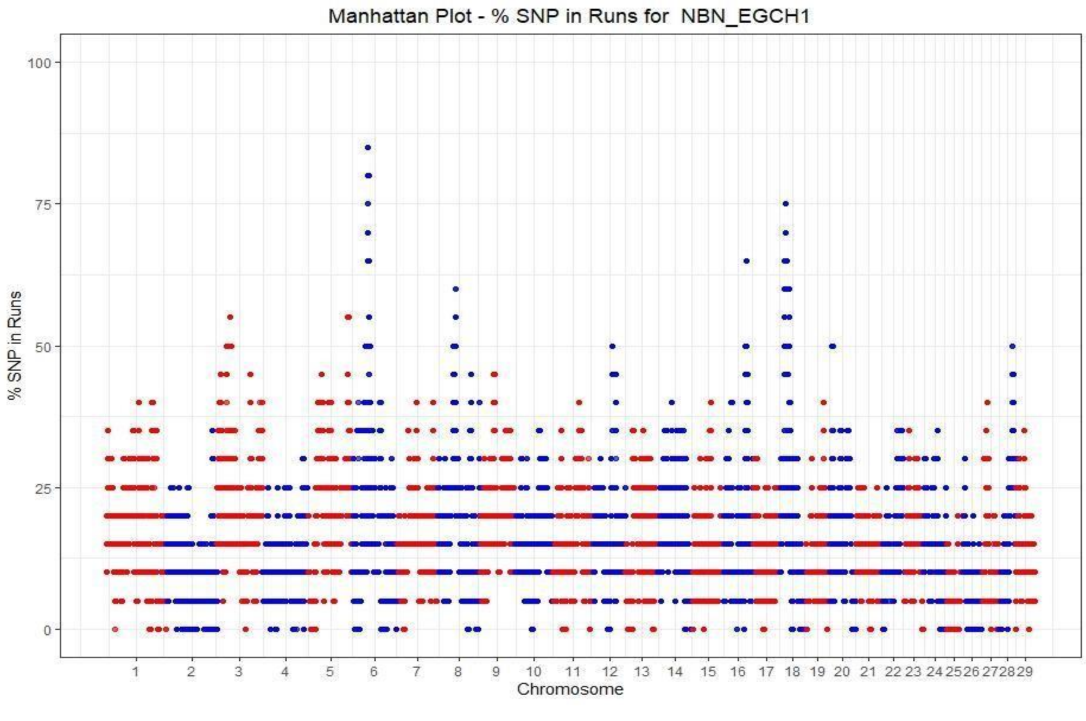
Manhattan Plot - % SNP in Runs for NBN_EGCH1
% SNP in Runs
100
75
50
25
0
1 2 3 4 5 6 7 8 9 10 11 12 13 14 15 16 17 18 19 20 21 22 23 24 25 26 27 28 29
Chromosome

Supplementary Figure 10. Manhattan plots generated by detectRUNS analysis for each population belonging to the Saanen goat breed. Each point corresponds to a SNP. Chromosomes are on the x-axis and the number of SNP inside the ROH is indicated on the y-axis.

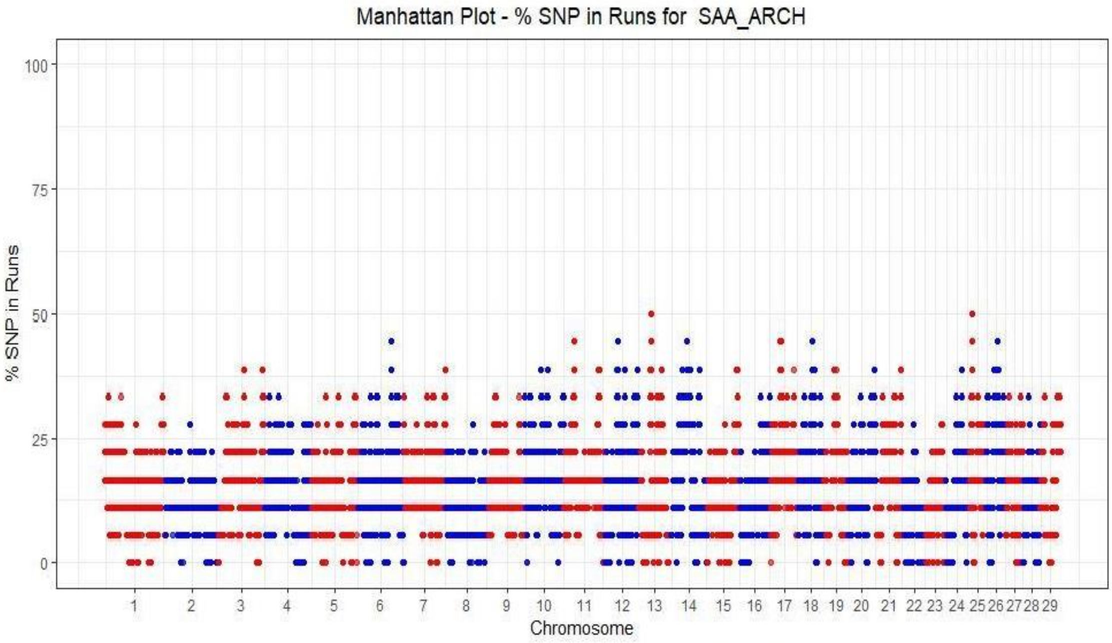

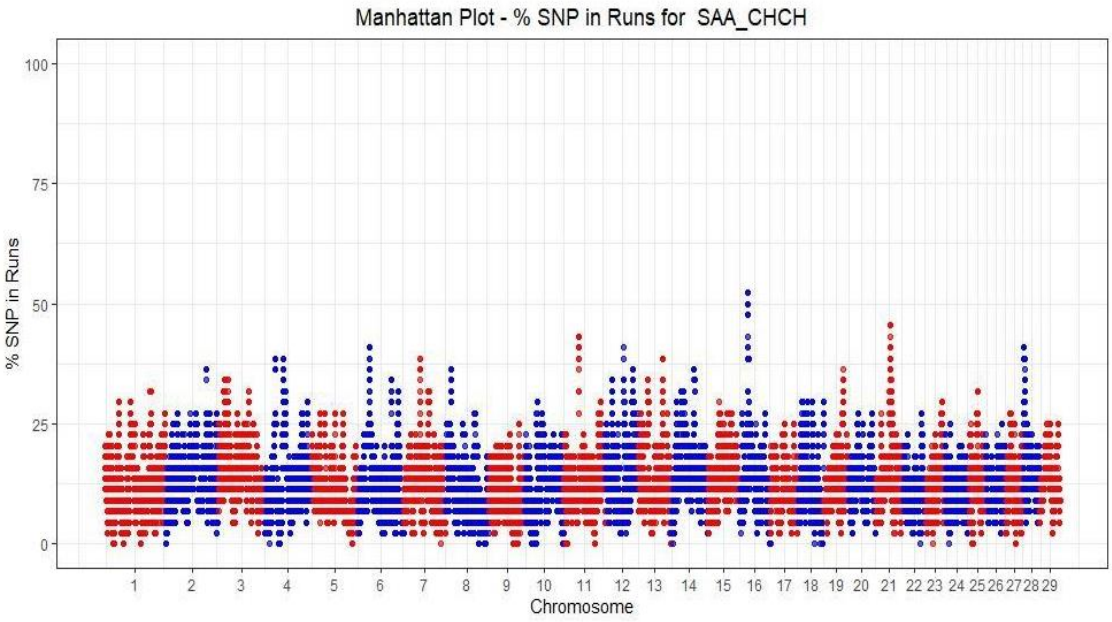

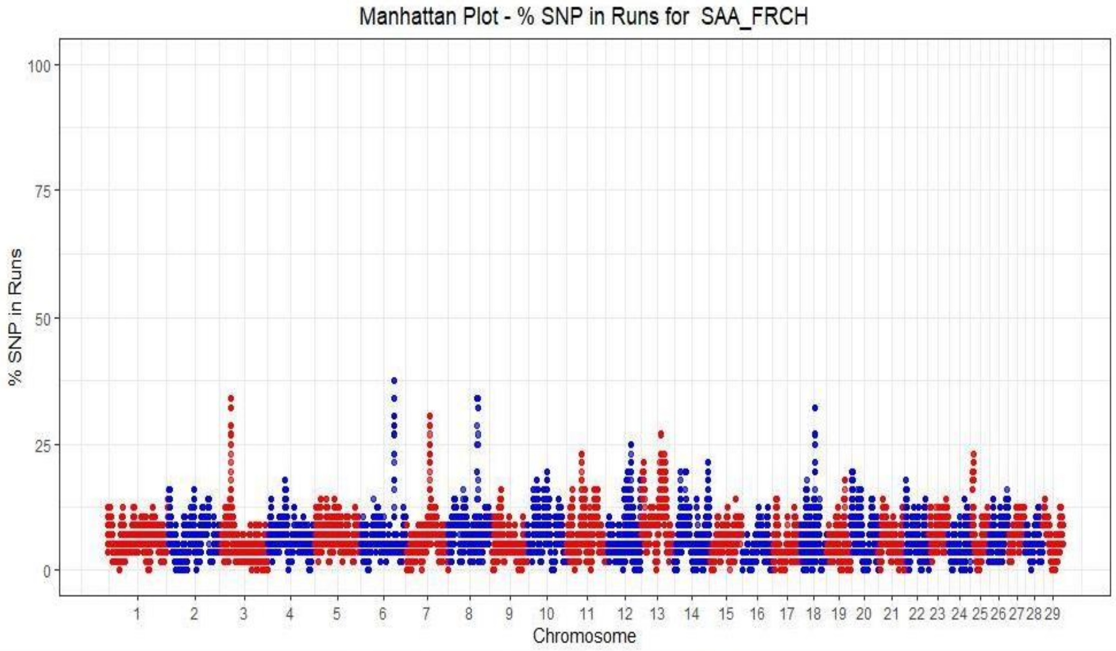
Manhattan Plot - % SNP in Runs for SAA_FRCH
100
75
50
25
0
% SNP in Runs
1 2 3 4 5 6 7 8 9 10 11 12 13 14 15 16 17 18 19 20 21 22 23 24 25 26 27 28 29
Chromosome

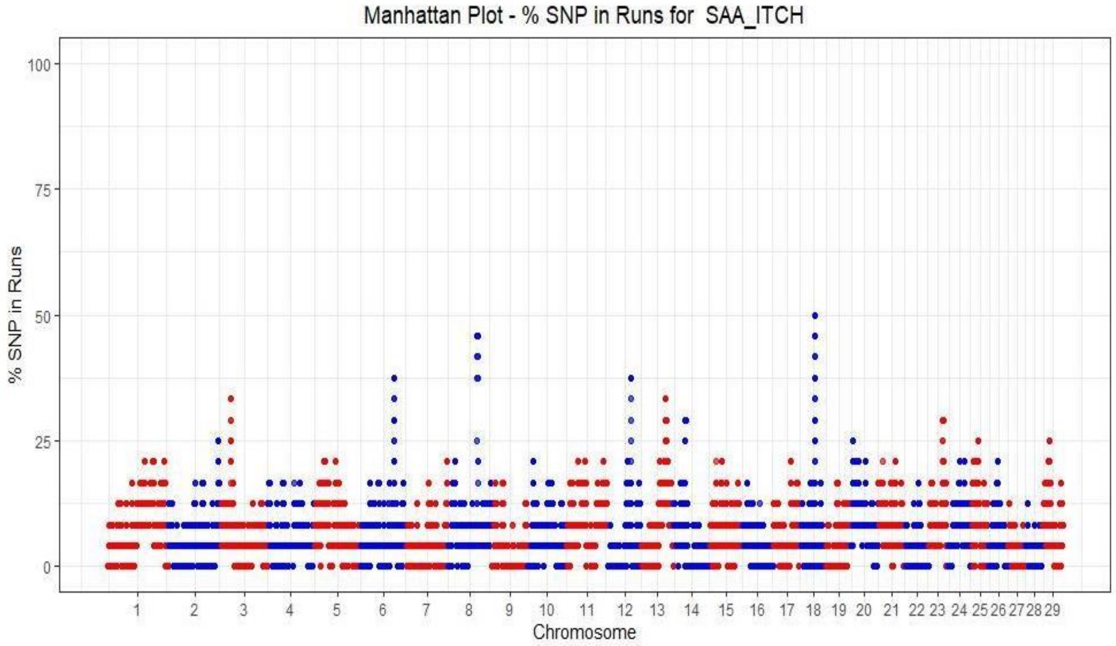
Manhattan Plot - % SNP in Runs for SAA_ITCH
100
75
50
25
0
% SNP in Runs
1 2 3 4 5 6 7 8 9 10 11 12 13 14 15 16 17 18 19 20 21 22 23 24 25 26 27 28 29
Chromosome

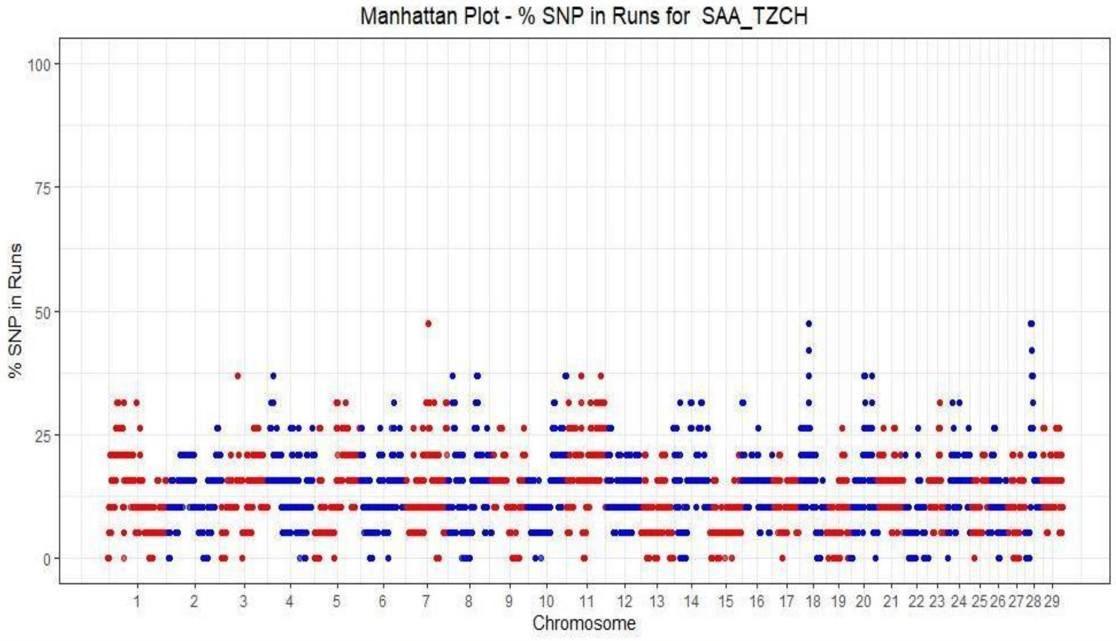
Manhattan Plot - % SNP in Runs for SAA_TZCH
% SNP in Runs
Chromosome
0
25
50
75
100
1 2 3 4 5 6 7 8 9 10 11 12 13 14 15 16 17 18 19 20 21 22 23 24 25 26 27 28 29

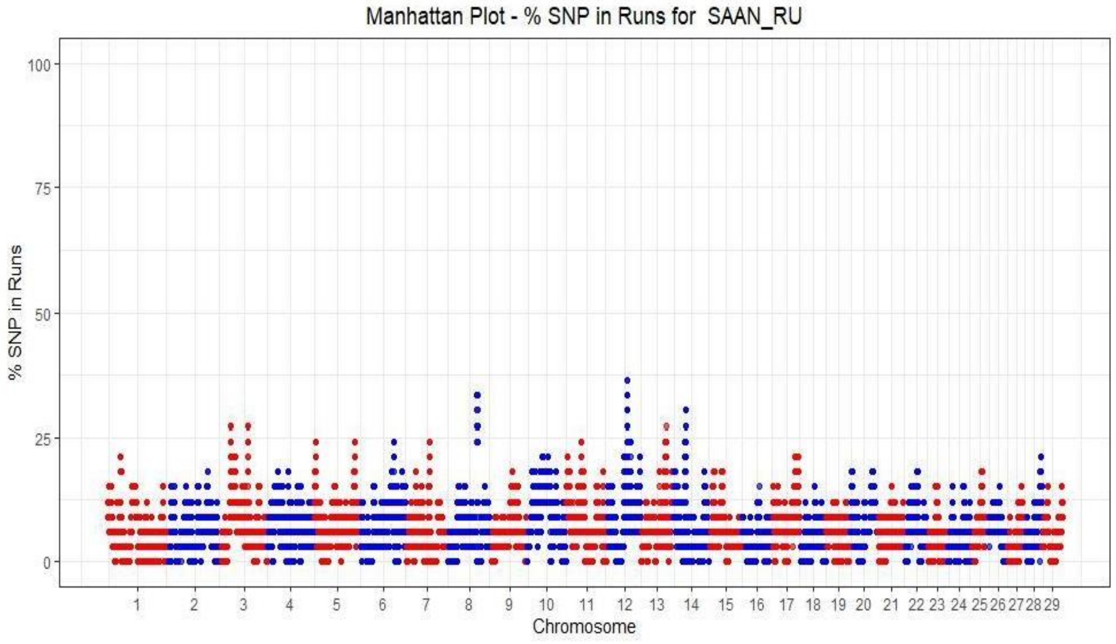
Manhattan Plot - % SNP in Runs for SAAN_RU
% SNP in Runs
Chromosome
0
25
50
75
100
1 2 3 4 5 6 7 8 9 10 11 12 13 14 15 16 17 18 19 20 21 22 23 24 25 26 27 28 29

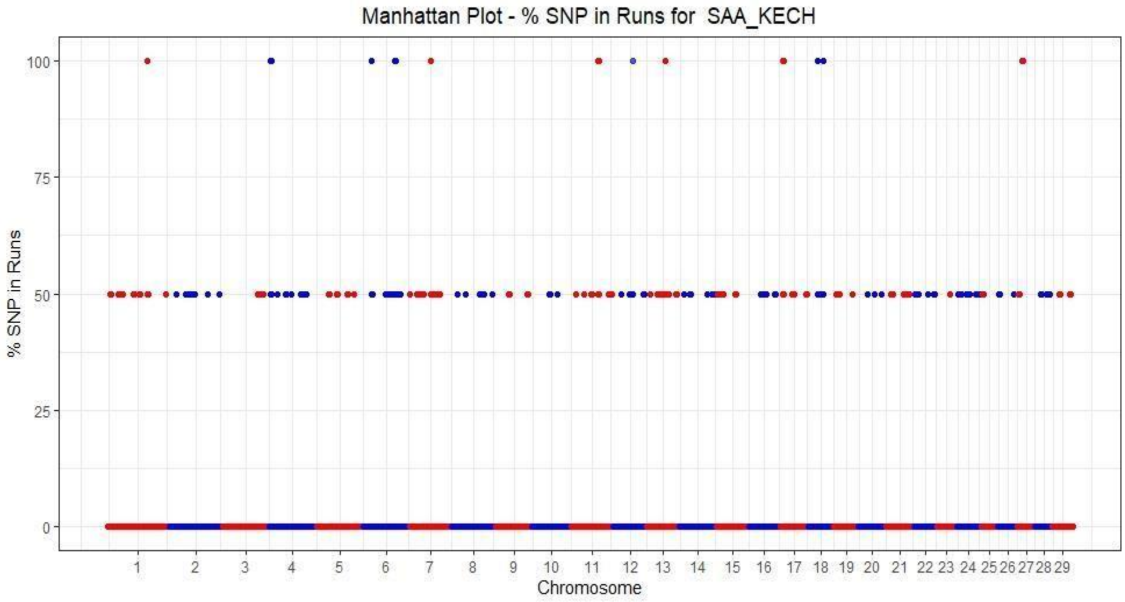

Manhattan Plot - % SNP in Runs for SAA_KECH
% SNP in Runs
100
75
50
25
0
1 2 3 4 5 6 7 8 9 10 11 12 13 14 15 16 17 18 19 20 21 22 23 24 25 26 27 28 29
Chromosome

Supplementary Figure 11. Mean of ROH for each individual. One different colour is assigned to the different populations belonging to each breed and the correspondence is explained in thelegend to the right side of the plot. A= Angora, B= Boer, C= Nubian, D= Saanen.

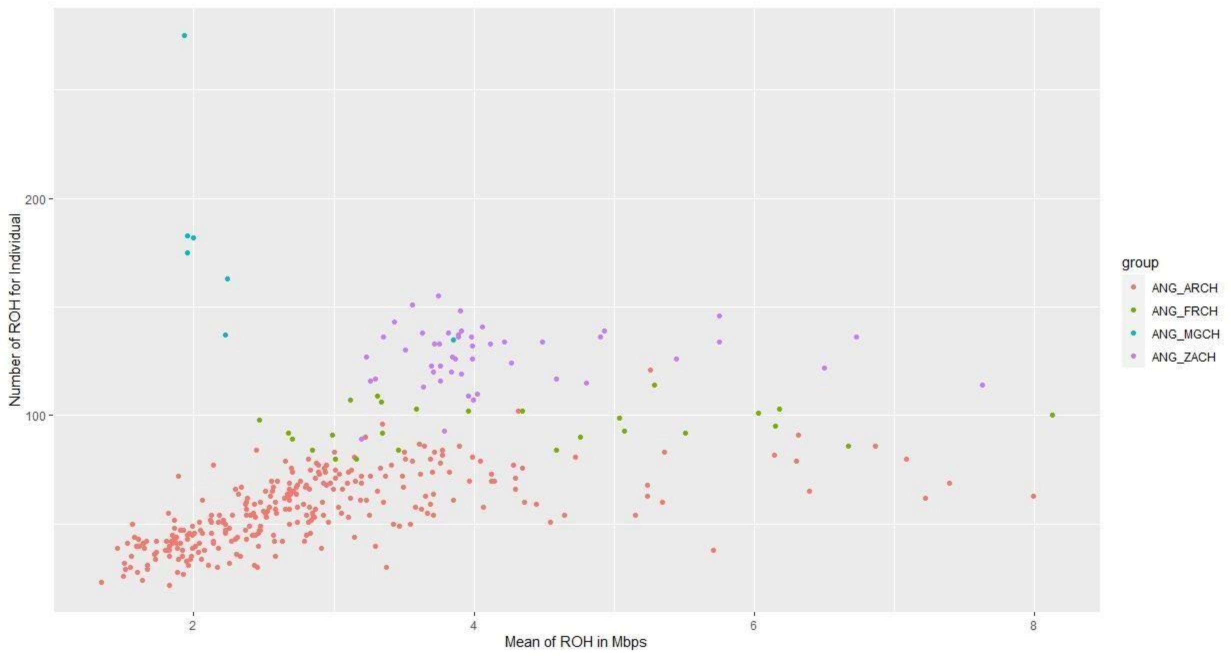

A

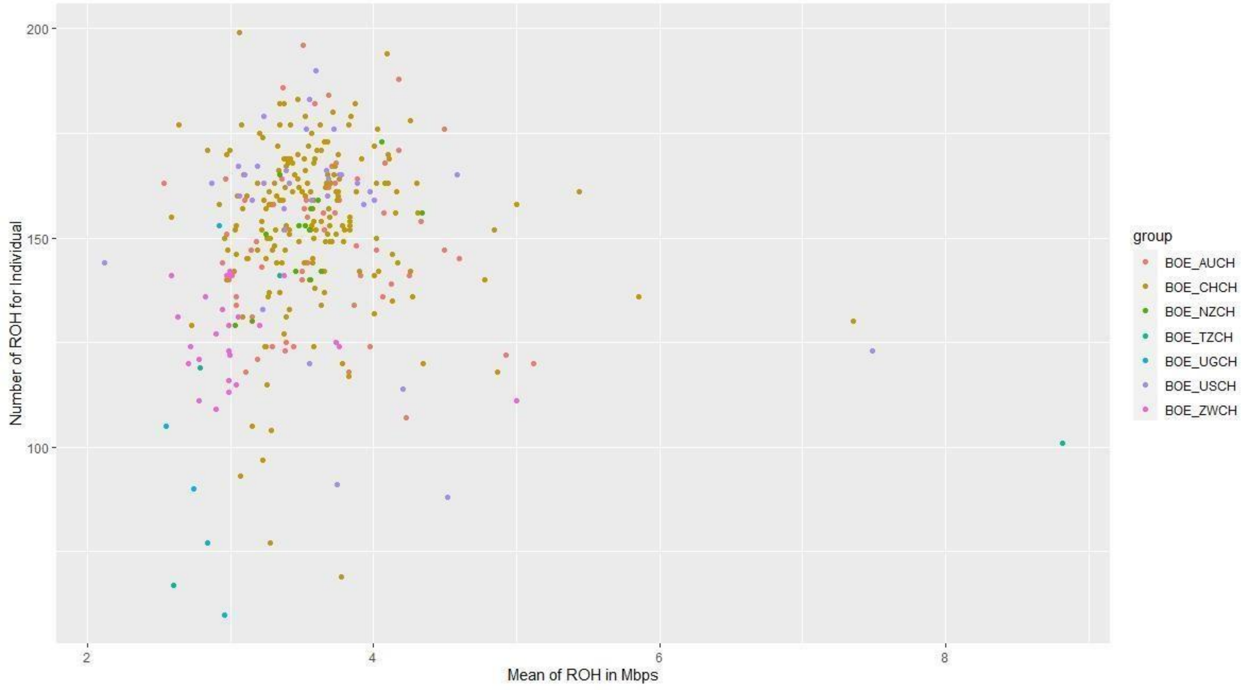

B

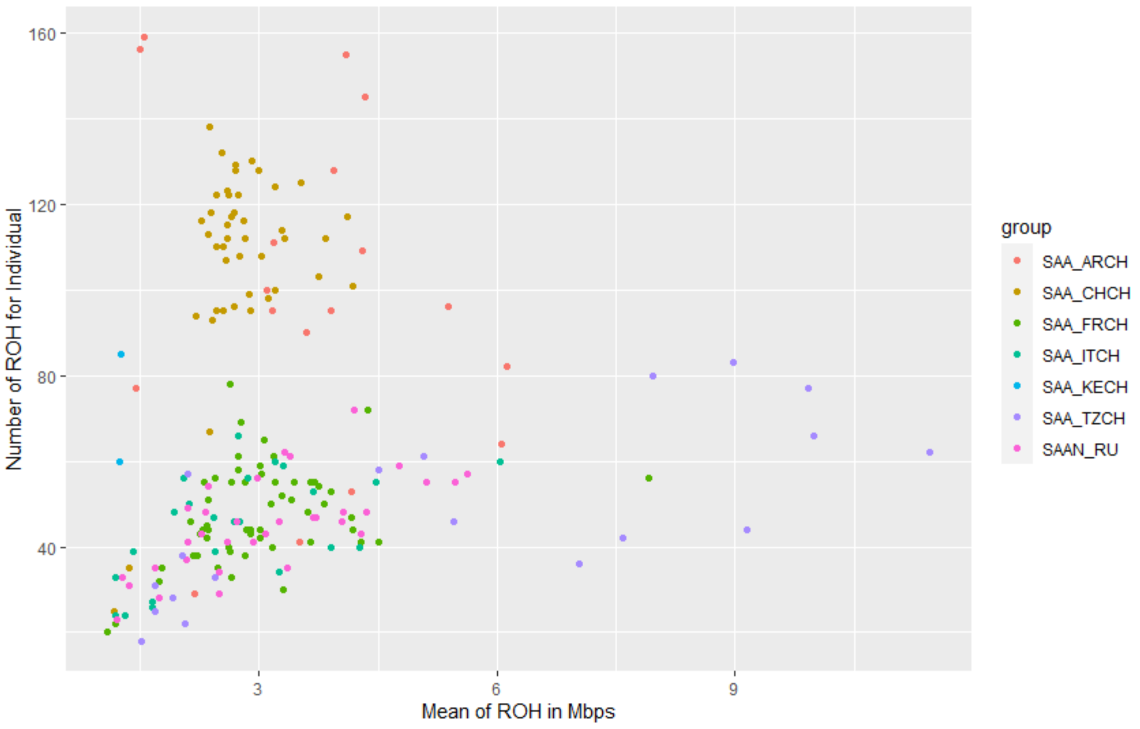
160
120
80
40
3
6
9
Number of ROH for Individual
Mean of ROH in Mbps
group
SAA_ARCH
SAA_CHCH
SAA_FRCH
SAA_ITCH
SAA_KECH
SAA_TZCH
SAAN_RU

C

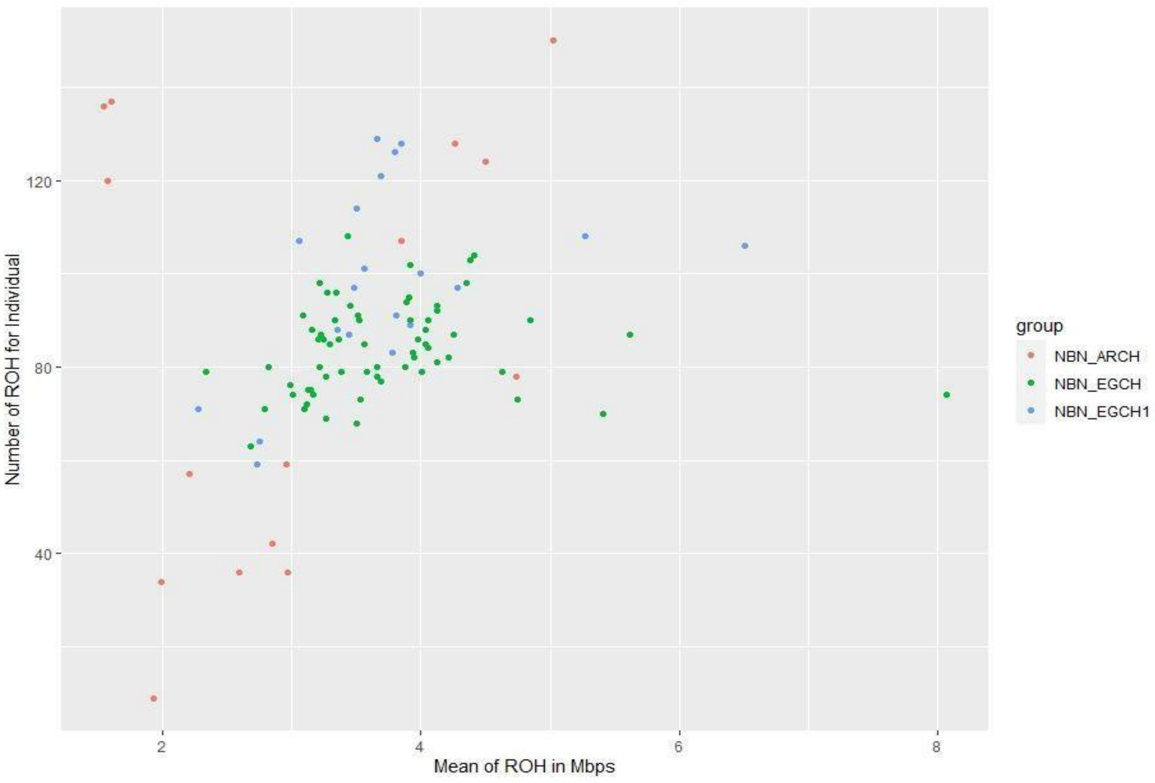
120
80
40
2
4
6
8
Number of ROH for Individual
Mean of ROH in Mbps
group
NBN_ARCH
NBN_EGCH
NBN_EGCH1

D

Supplementary Figure 12

Sum of ROH for each individual. One different colour is assigned to the different populations belonging to each breed and the correspondence is explained in the legend to the right side of the plot. A= Angora, B= Boer, C= Nubian, D= Saanen.

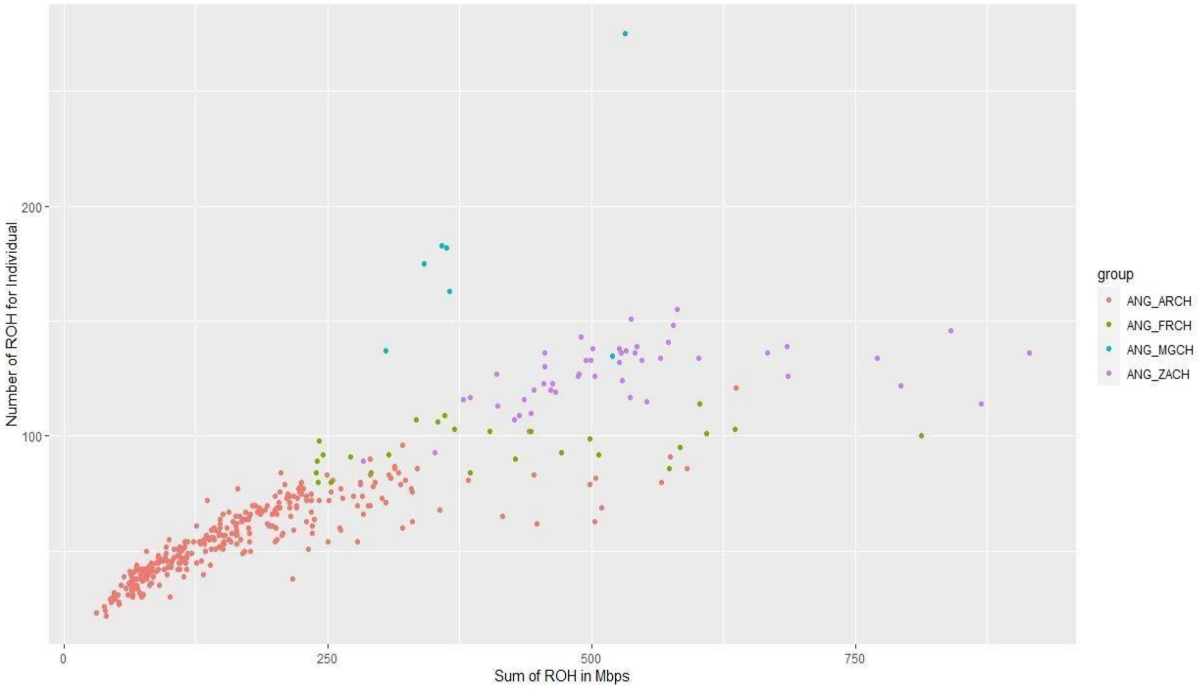

A

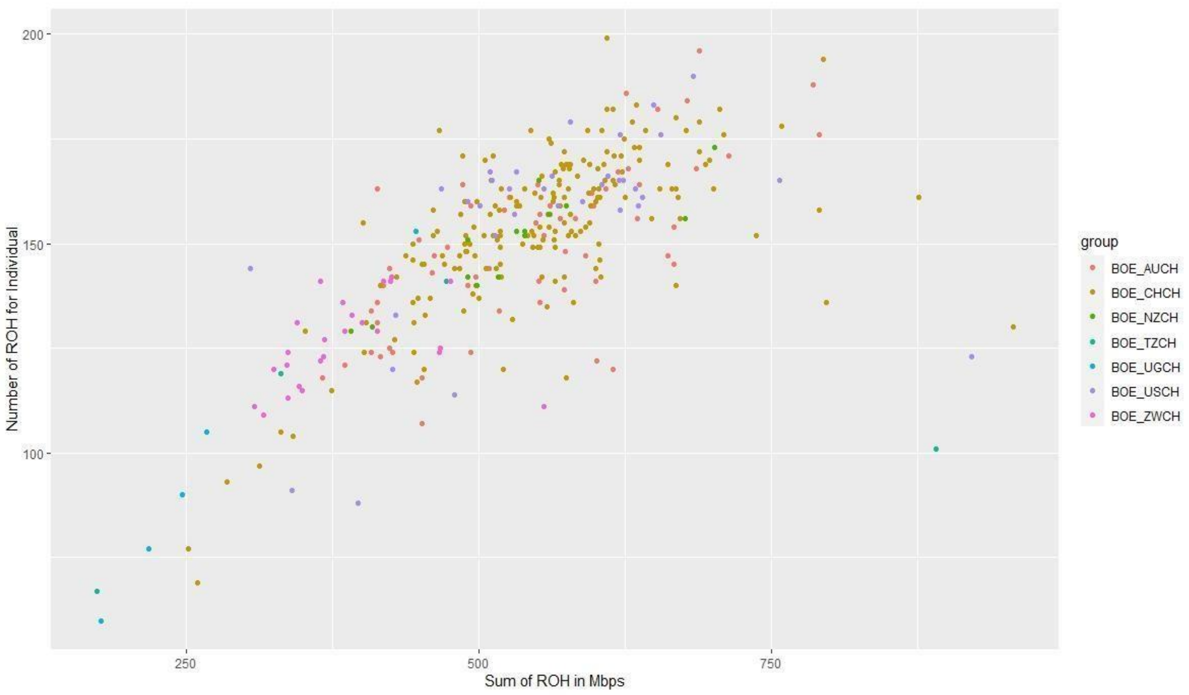

B

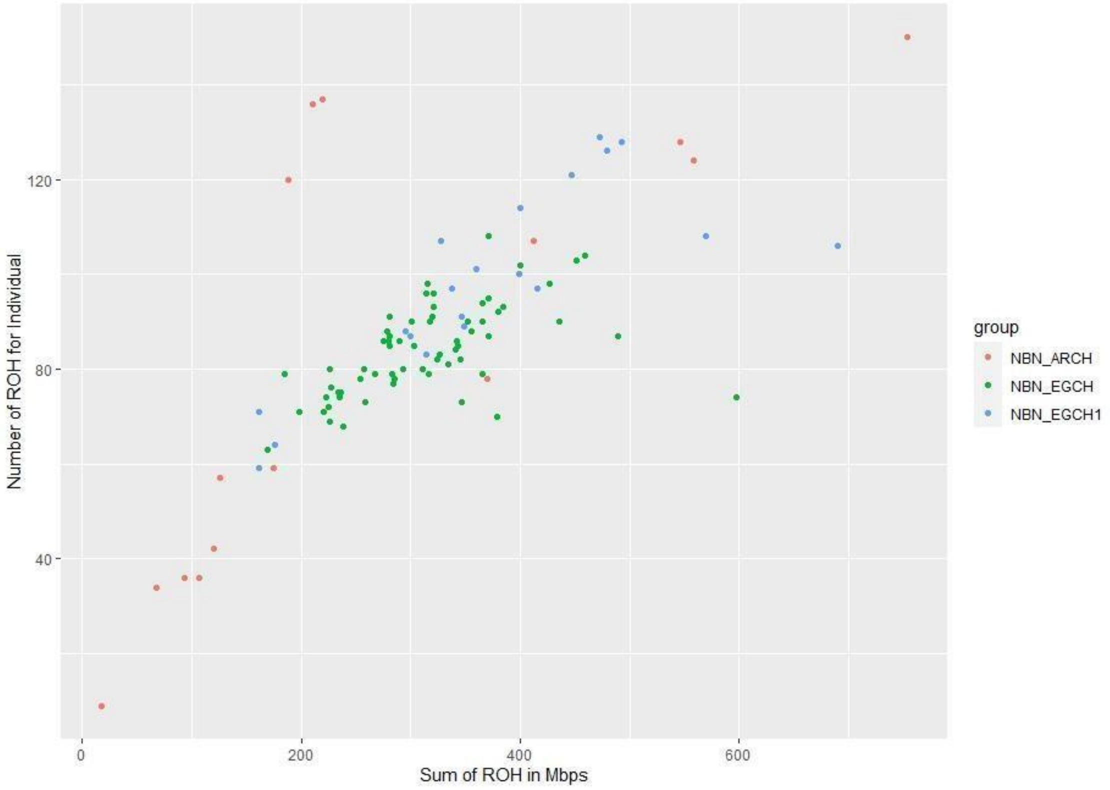

120
80
40
0
200
400
600
Number of ROH for Individual
Sum of ROH in Mbps
group
NBN_ARCH
NBN_EGCH
NBN_EGCH1

C

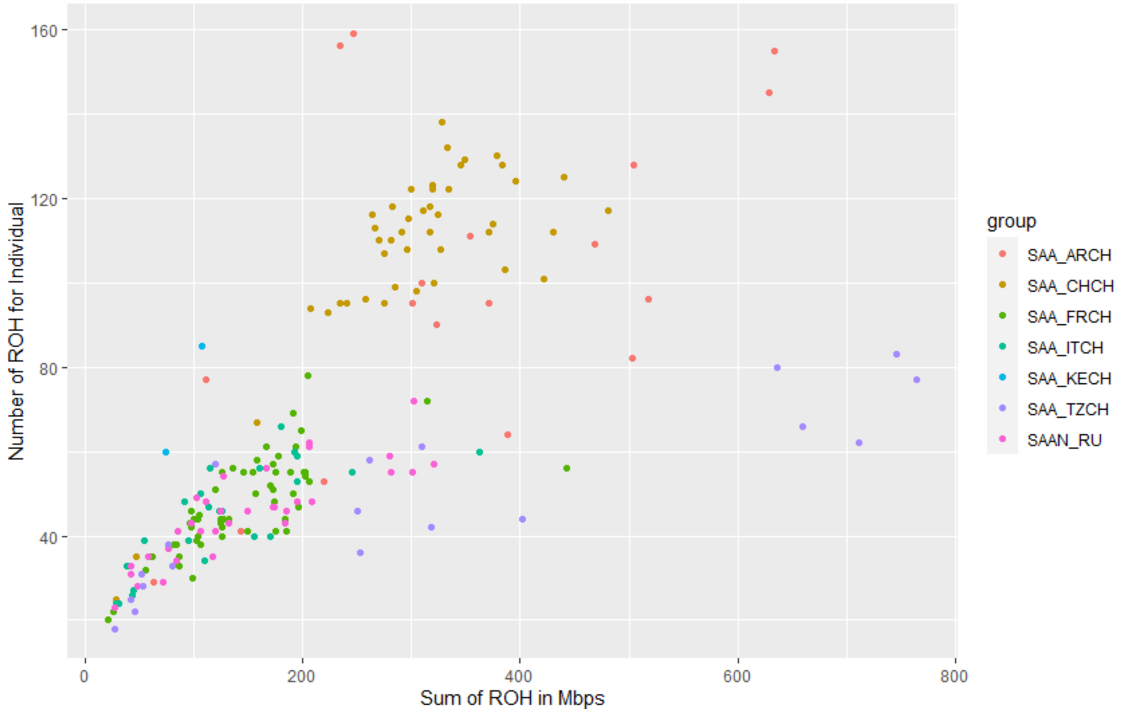

160
120
80
40
0
200
400
600
800
Number of ROH for Individual
Sum of ROH in Mbps
group
SAA_ARCH
SAA_CHCH
SAA_FRCH
SAA_ITCH
SAA_KECH
SAA_TZCH
SAAN_RU

D
