# Supplementary material for: Discovering novel clues of natural selection on four worldwide goat breeds
Source: Sci Rep. 2023 Feb 6;13:2110. doi: 10.1038/s41598-023-27490-x (PMC9902602; doi:10.1038/s41598-023-27490-x)
Supplement: Supplementary file 7 — Supplementary Information 7. [file 41598_2023_27490_MOESM7_ESM.docx]

**Supplementary Table 1**. Pairwise F_ST_ for each breed recalculated for this study.

| **ANGORA** | ANG_ARCH | ANG_FRCH | ANG_MGCH | ANG_ZACH |
| --- | --- | --- | --- | --- |
| ANG_ARCH | - |  |  |  |
| ANG_FRCH | 0.064 | - |  |  |
| ANG_MGCH | 0.113 | 0.153 | - | - |
| ANG_ZACH | 0.067 | 0.134 | 0.172 |  |

| **BOER** | BOE_  AUCH | BOE_  CHCH | BOE_  NZCH | BOE_  TZCH | BOE_  UGCH | BOE_  USCH | BOE_  ZWCH |
| --- | --- | --- | --- | --- | --- | --- | --- |
| BOE_AUCH | - |  |  |  |  |  |  |
| BOE_CHCH | 0.039 | - |  |  |  |  |  |
| BOE_NZCH | 0.037 | 0.036 | - |  |  |  |  |
| BOE_TZCH | 0.056 | 0.042 | 0.049 | - |  |  |  |
| BOE_UGCH | 0.055 | 0.040 | 0.043 | 0.028 | - |  |  |
| BOE_USCH | 0.029 | 0.019 | 0.019 | 0.037 | 0.033 | - |  |
| BOE_ZWCH | 0.073 | 0.061 | 0.065 | 0.065 | 0.060 | 0.054 | - |

| **NUBIAN** | NBN_ARCH | NBN_EGCH | NBN_EGCH1 |
| --- | --- | --- | --- |
| NBN_ARCH | - |  |  |
| NBN_EGCH | 0.115 | - |  |
| NBN_EGCH1 | 0.121 | 0.074 | - |

| **SAANEN** | SAA_  ARCH | SAA_  CHCH | SAA_  FRCH | SAA_  ITCH | SAA_  KECH | SAA_  TZCH | SAA_  RU |
| --- | --- | --- | --- | --- | --- | --- | --- |
| SAA_ARCH | - |  |  |  |  |  |  |
| SAA_CHCH | 0.087 | - |  |  |  |  |  |
| SAA_FRCH | 0.040 | 0.067 | - |  |  |  |  |
| SAA_ITCH | 0.043 | 0.060 | 0.009 | - |  |  |  |
| SAA_KECH | 0.102 | 0.192 | 0.131 | 0.137 | - |  |  |
| SAA_TZCH | 0.066 | 0.122 | 0.079 | 0.082 | 0.117 | - |  |
| SAAN_RU | 0.034 | 0.063 | 0.026 | 0.029 | 0.118 | 0.072 | - |

**Supplementary Table 2.** SNP outliers found with PCadapt analysis corresponding to the dataset of Nubian breed. before the more stringent filtering. The outliers are significant after Bonferroni’s correction.

| **SNP** | **Chromosome** | **Position bp** | **P-value** |
| --- | --- | --- | --- |
| scaffold640-19604 | 1 | 24806876 | 1.93E-05 |
| scaffold234-223305 | 1 | 25048419 | 1.11E-06 |
| scaffold1480-342600 | 1 | 47238651 | 2.50E-05 |
| scaffold306-140624 | 1 | 64366087 | 9.27E-05 |
| scaffold214-1862087 | 1 | 68806469 | 2.05E-05 |
| scaffold377-373657 | 1 | 85954509 | 0.02915325 |
| scaffold65-2970760 | 1 | 107099865 | 0.00038296 |
| scaffold1105-1147118 | 1 | 129672870 | 0.00679917 |
| scaffold220-862988 | 2 | 2704062 | 5.50E-05 |
| scaffold220-805268 | 2 | 2761810 | 2.90E-06 |
| scaffold670-1179894 | 2 | 10463291 | 1.79E-12 |
| scaffold631-655827 | 2 | 41557749 | 1.69E-16 |
| scaffold82-1513501 | 2 | 50281528 | 7.79E-06 |
| scaffold1489-481443 | 2 | 59233125 | 4.50E-05 |
| scaffold1200-700104 | 2 | 60184268 | 0.00013005 |
| scaffold1067-634195 | 2 | 66651654 | 0.00011095 |
| scaffold614-6654844 | 2 | 83343747 | 0.00017272 |
| scaffold790-847997 | 3 | 5618660 | 2.12E-15 |
| scaffold543-3073389 | 3 | 11168070 | 0.02882722 |
| scaffold1704-237124 | 3 | 15155116 | 2.46E-18 |
| scaffold687-633011 | 3 | 38106422 | 2.97E-09 |
| scaffold144-3561511 | 3 | 49305057 | 1.10E-05 |
| scaffold190-674518 | 3 | 62440182 | 0.00372036 |
| scaffold164-3052181 | 3 | 76665007 | 0.00137006 |
| scaffold659-1230265 | 3 | 96503671 | 0.00087002 |
| scaffold737-285083 | 3 | 98201775 | 0.00046853 |
| scaffold180-742677 | 3 | 100143884 | 2.96E-10 |
| scaffold4404-41796 | 3 | 101377443 | 0.00445318 |
| scaffold73-254628 | 3 | 101991367 | 3.51E-05 |
| scaffold2044-453011 | 4 | 1257240 | 5.17E-11 |
| scaffold494-6384135 | 4 | 43748046 | 0.00160808 |
| scaffold641-378530 | 4 | 118757652 | 1.92E-06 |
| scaffold399-1253481 | 5 | 2293959 | 1.14E-09 |
| scaffold399-541813 | 5 | 3006775 | 0.00031723 |
| scaffold157-390219 | 5 | 19733227 | 0.01929694 |
| scaffold1393-1065170 | 5 | 31983701 | 2.12E-06 |
| scaffold920-434644 | 5 | 42494415 | 0.03824099 |
| scaffold712-285403 | 5 | 50867311 | 7.90E-06 |
| scaffold712-793376 | 5 | 51380235 | 0.0004471 |
| scaffold107-4539893 | 5 | 80765343 | 4.81E-23 |
| scaffold1322-89443 | 5 | 86654136 | 5.36E-13 |
| scaffold740-2520610 | 5 | 87722650 | 2.50E-05 |
| scaffold740-2481302 | 5 | 87762049 | 0.00252804 |
| scaffold666-379511 | 5 | 96695139 | 0.00035513 |
| scaffold1657-22752 | 5 | 116979586 | 0.03972104 |
| scaffold999-35964 | 6 | 1951800 | 6.76E-09 |
| scaffold340-1028551 | 6 | 14358997 | 2.23E-05 |
| scaffold968-677832 | 6 | 29025606 | 0.00230343 |
| scaffold968-2472589 | 6 | 30831884 | 9.42E-08 |
| scaffold148-3457759 | 6 | 34602486 | 1.65E-08 |
| scaffold281-747096 | 6 | 37401587 | 0.01754726 |
| scaffold281-1816828 | 6 | 38473627 | 0.02194042 |
| scaffold281-2039192 | 6 | 38696985 | 9.51E-09 |
| scaffold281-2636514 | 6 | 39295173 | 1.01E-09 |
| scaffold1268-14447 | 6 | 39985135 | 0.00189724 |
| scaffold1273-10351 | 6 | 40745285 | 0.00126603 |
| scaffold1273-139768 | 6 | 40875771 | 9.44E-10 |
| scaffold821-569788 | 6 | 41727639 | 3.15E-06 |
| scaffold1199-856786 | 6 | 43160722 | 3.64E-12 |
| scaffold771-245006 | 6 | 46214946 | 0.00167365 |
| scaffold241-296190 | 6 | 49627140 | 0.03034261 |
| scaffold42-974605 | 6 | 52110326 | 1.71E-05 |
| scaffold42-849744 | 6 | 52236308 | 1.09E-05 |
| scaffold289-2632133 | 6 | 59096273 | 0.01323849 |
| scaffold1281-174261 | 6 | 91457367 | 0.00171959 |
| scaffold539-678975 | 6 | 93119275 | 1.75E-09 |
| scaffold690-1007687 | 6 | 107266984 | 5.33E-12 |
| scaffold2951-62362 | 6 | 115573662 | 0.00037923 |
| scaffold1698-77702 | 6 | 116572235 | 1.58E-09 |
| scaffold1539-76119 | 7 | 712000 | 3.88E-07 |
| scaffold12-96833 | 7 | 22429407 | 0.00281415 |
| scaffold316-398273 | 7 | 87956771 | 1.34E-12 |
| scaffold85-1952236 | 7 | 90294761 | 0.00022395 |
| scaffold85-1981640 | 7 | 90322724 | 3.26E-10 |
| scaffold85-2217820 | 7 | 90555022 | 0.00974949 |
| scaffold1816-12146 | 7 | 92698716 | 0.0049611 |
| scaffold1931-325394 | 7 | 95332783 | 3.65E-05 |
| scaffold2964-8032 | 7 | 99842640 | 0.0038565 |
| scaffold510-1954512 | 7 | 102936661 | 0.00773126 |
| scaffold16-354861 | 8 | 10445475 | 1.74E-06 |
| scaffold606-5228676 | 8 | 54499252 | 5.78E-05 |
| scaffold1454-717418 | 8 | 56724585 | 1.85E-07 |
| scaffold497-1065156 | 8 | 83254950 | 0.00018229 |
| scaffold359-699029 | 9 | 12448382 | 1.44E-17 |
| scaffold524-844497 | 9 | 26094636 | 0.00021648 |
| scaffold1159-1334940 | 9 | 39555783 | 0.00013067 |
| scaffold427-2278546 | 9 | 42861450 | 0.00409715 |
| scaffold1423-261833 | 9 | 68434441 | 1.08E-06 |
| scaffold448-3611060 | 9 | 76525804 | 0.01361096 |
| scaffold447-4082549 | 9 | 80461056 | 0.00022856 |
| scaffold271-446145 | 10 | 14801887 | 0.00913806 |
| scaffold133-1775977 | 10 | 17762769 | 0.00771712 |
| scaffold104-1406404 | 10 | 26111616 | 2.21E-13 |
| scaffold104-3871768 | 10 | 28622786 | 2.37E-12 |
| scaffold662-308388 | 10 | 39021630 | 0.0001116 |
| scaffold68-2874056 | 10 | 50631087 | 1.15E-05 |
| scaffold68-3827001 | 10 | 51578278 | 9.37E-06 |
| scaffold618-1267442 | 11 | 18273832 | 0.00991011 |
| scaffold52-2585171 | 11 | 29634064 | 5.34E-06 |
| scaffold247-8163187 | 11 | 45658214 | 0.00147247 |
| scaffold656-1117519 | 11 | 53730290 | 0.00146105 |
| scaffold185-11191589 | 11 | 62684733 | 1.06E-10 |
| scaffold804-2344420 | 11 | 98388947 | 1.79E-05 |
| scaffold1336-1458060 | 12 | 7301479 | 0.00287802 |
| scaffold717-7760530 | 12 | 57389946 | 2.09E-07 |
| scaffold717-6126750 | 12 | 59028213 | 0.00047076 |
| scaffold717-312862 | 12 | 64843796 | 1.01E-05 |
| scaffold70-543914 | 12 | 81452421 | 0.00044895 |
| scaffold371-925768 | 13 | 40590432 | 0.03100239 |
| scaffold14-190949 | 14 | 21232585 | 1.77E-06 |
| scaffold1370-223346 | 14 | 41848208 | 0.04568718 |
| scaffold1641-366560 | 14 | 45459456 | 1.64E-10 |
| scaffold1751-71296 | 14 | 91885536 | 1.33E-10 |
| scaffold953-464186 | 15 | 35438051 | 0.00679373 |
| scaffold845-729661 | 16 | 18155680 | 7.59E-05 |
| scaffold299-3166651 | 16 | 76570293 | 0.02604009 |
| scaffold1586-436664 | 17 | 6613662 | 0.04452725 |
| scaffold663-84666 | 17 | 67390408 | 2.59E-10 |
| scaffold779-482877 | 17 | 70294093 | 2.44E-10 |
| scaffold43-487975 | 18 | 25613620 | 3.49E-10 |
| scaffold1344-2516800 | 18 | 41693302 | 0.00152491 |
| scaffold1038-2191182 | 19 | 3115880 | 0.00058327 |
| scaffold502-738094 | 19 | 11441018 | 4.95E-05 |
| scaffold1229-208239 | 19 | 23553648 | 3.59E-06 |
| scaffold1328-466464 | 19 | 29567262 | 1.23E-08 |
| scaffold711-758192 | 20 | 20621746 | 0.01031057 |
| scaffold240-1773419 | 21 | 14199268 | 0.00187501 |
| scaffold1265-72004 | 21 | 18887929 | 0.00495438 |
| scaffold160-105988 | 21 | 26680802 | 0.00022295 |
| scaffold160-1114476 | 21 | 27691053 | 0.00144091 |
| scaffold154-2323816 | 22 | 31785158 | 2.97E-13 |
| scaffold827-4548148 | 22 | 45713484 | 0.00798923 |
| scaffold1497-2180770 | 22 | 52318319 | 0.00112921 |
| scaffold1869-358427 | 22 | 55275038 | 0.0044312 |
| scaffold505-165819 | 23 | 10931371 | 0.03423909 |
| scaffold994-280293 | 24 | 5186568 | 3.72E-05 |
| scaffold1022-261355 | 24 | 6836509 | 0.00727089 |
| scaffold1579-16472 | 24 | 34188996 | 0.01276285 |
| scaffold2188-369505 | 25 | 153084 | 1.93E-05 |
| scaffold1605-234785 | 25 | 34378956 | 1.11E-06 |
| scaffold973-1710364 | 26 | 49032698 | 2.50E-05 |
| scaffold509-1101654 | 28 | 11746431 | 9.27E-05 |
| scaffold509-2468879 | 28 | 13109386 | 2.05E-05 |
| scaffold838-3792681 | 28 | 27893879 | 0.02915325 |
| scaffold274-631324 | 29 | 31350789 | 0.00038296 |
| scaffold799-4952512 | 29 | 37432968 | 0.00679917 |
| scaffold1587-647949 | 29 | 47667204 | 5.50E-05 |
| scaffold695-114323 | 29 | 49757396 | 2.90E-06 |

**Supplementary Table 3.** Fst and FROH values for the four breeds. For the F_ST_ we compared our results to which of Colli et al.2018. FROH were obtained in this study.

| **NUBIAN** | ARCH | EGCH | EGCH1 |
| --- | --- | --- | --- |
| ARCH | - | - | - |
| EGCH | 0.115 | - | - |
| EGCH1 | 0.121 | 0.074 | - |
| **FROH** | 0.11 | 0.13 | 0.14 |

| **ANGORA** | ARCH | FRCH | MGCH | ZACH |
| --- | --- | --- | --- | --- |
| ARCH | - | - | - | - |
| FRCH | 0.068 | - | - | - |
| MGCH | 0.119 | 0.158 | - | - |
| ZACH | 0.074 | 0.135 | 0.178 | - |
| **FROH** | 0.069 | 0.167 | 0.161 | 0.217 |

| **SAANEN** | ARCH | CHCH | FRCH | ITCH | KECH | TZCH | RU |
| --- | --- | --- | --- | --- | --- | --- | --- |
| ARCH | - | - | - | - | - | - | - |
| CHCH | 0.087 | - | - | - | - | - | - |
| FRCH | 0.040 | 0.066 | - | - | - | - | - |
| ITCH | 0.043 | 0.059 | 0.009 | - | - | - | - |
| KECH | 0.102 | 0.192 | 0.131 | 0.137 | - | - | - |
| TZCH | 0.066 | 0.122 | 0.079 | 0.082 | 0.117 | - | - |
| RU | 0.034 | 0.063 | 0.025 | 0.029 | 0.118 | 0.072 | - |
| **FROH** | **0.142** | **0.123** | **0.059** | **0.055** | **0.035** | **0.124** | **0.060** |

| **BOER** | AUCH | CHCH | NZCH | TZCH | UGCH | USCH | ZWCH |
| --- | --- | --- | --- | --- | --- | --- | --- |
| AUCH | - | - | - | - | - | - | - |
| CHCH | 0.039 | - | - | - | - | - | - |
| NZCH | 0.037 | 0.035 | - | - | - | - | - |
| TZCH | 0.056 | 0.041 | 0.049 | - | - | - | - |
| UGCH | 0.055 | 0.040 | 0.043 | 0.027 | - | - | - |
| USCH | 0.028 | 0.019 | 0.019 | 0.037 | 0.033 | - | - |
| ZWCH | 0.072 | 0.061 | 0.064 | 0.065 | 0.060 | 0.054 | - |
| **FROH** | **0.220** | **0.225** | **0.216** | **0.189** | **0.110** | **0227** | **0.157** |

**Supplementary Table 3**. The complete list of Runs Of Homozygosity hotspots (ROH islands) discovered by detectRUNS R package for each breed and population. The number of markers that fall into the homozygous stretch is also indicated.

| Breed | Population | chr | Nº SNP | from | to |
| --- | --- | --- | --- | --- | --- |
| NUBIAN | ARCH | 12 | 12 | 43300149 | 44644947 |
|  | ARCH | 17 | 17 | 25127993 | 25405707 |
|  | EGCH | 25 | 25 | 18411314 | 21266675 |
|  | EGCH | 6 | 6 | 12801008 | 13020321 |
|  | EGCH | 6 | 6 | 13133655 | 14648765 |
|  | EGCH | 6 | 6 | 33660412 | 34924471 |
|  | EGCH | 6 | 6 | 37368764 | 39573331 |
|  | EGCH | 6 | 6 | 39985135 | 40254822 |
|  | EGCH | 6 | 6 | 40426392 | 41727639 |
|  | EGCH | 6 | 6 | 45625400 | 45863398 |
|  | EGCH | 6 | 6 | 85861248 | 86083934 |
|  | EGCH1 | 16 | 16 | 60235603 | 60740031 |
|  | EGCH1 | 18 | 18 | 10575697 | 11801703 |
|  | EGCH1 | 18 | 18 | 11864422 | 12213029 |
|  | EGCH1 | 18 | 18 | 13626437 | 16328074 |
|  | EGCH1 | 18 | 18 | 22246313 | 22674088 |
|  | EGCH1 | 3 | 3 | 34702985 | 35005840 |
|  | EGCH1 | 5 | 5 | 105913611 | 106479948 |
|  | EGCH1 | 6 | 6 | 39243101 | 41727639 |
|  | EGCH1 | 6 | 6 | 42955958 | 44170141 |
|  | EGCH1 | 8 | 8 | 45102350 | 45575107 |

| Breed | Population | chr | Nº SNP | from | to |
| --- | --- | --- | --- | --- | --- |
| ANGORA | FRCH | 10 | 21 | 85924553 | 86871754 |
|  | FRCH | 13 | 17 | 62717765 | 63686716 |
|  | FRCH | 18 | 13 | 14430468 | 15301856 |
|  | FRCH | 20 | 23 | 27198225 | 28379308 |
|  | FRCH | 21 | 7 | 51578120 | 52000410 |
|  | FRCH | 24 | 7 | 47985140 | 48357396 |
|  | FRCH | 29 | 14 | 13328789 | 14108382 |
|  | MGCH | 1 | 54 | 64022844 | 67339400 |
|  | MGCH | 1 | 50 | 83493210 | 86043145 |
|  | MGCH | 1 | 24 | 99076878 | 100493318 |
|  | MGCH | 1 | 5 | 100861790 | 101127527 |
|  | MGCH | 1 | 28 | 102235149 | 103686836 |
|  | MGCH | 1 | 5 | 107538971 | 107714492 |
|  | MGCH | 1 | 7 | 108096270 | 108393850 |
|  | MGCH | 1 | 13 | 109409915 | 110225650 |
|  | MGCH | 1 | 8 | 127678022 | 127963519 |
|  | MGCH | 1 | 17 | 128375725 | 129146430 |
|  | MGCH | 1 | 42 | 134800898 | 136903242 |
|  | MGCH | 1 | 7 | 141017992 | 141294910 |
|  | MGCH | 10 | 19 | 47855663 | 48783621 |
|  | MGCH | 10 | 16 | 48980133 | 49836402 |
|  | MGCH | 10 | 7 | 50111939 | 50631087 |
|  | MGCH | 11 | 13 | 53019981 | 53730290 |
|  | MGCH | 11 | 7 | 87999632 | 88509752 |
|  | MGCH | 11 | 24 | 93648434 | 94789872 |
|  | MGCH | 12 | 21 | 50554041 | 51611236 |
|  | MGCH | 12 | 23 | 53313885 | 54494131 |
|  | MGCH | 12 | 9 | 57770691 | 58238170 |
|  | MGCH | 13 | 21 | 9835530 | 10656880 |
|  | MGCH | 13 | 14 | 56314706 | 57241503 |
|  | MGCH | 14 | 27 | 43351920 | 44690618 |
|  | MGCH | 14 | 38 | 75022013 | 77182043 |
|  | MGCH | 16 | 15 | 27164243 | 27798106 |
|  | MGCH | 16 | 25 | 75343695 | 76570293 |
|  | MGCH | 17 | 13 | 5213285 | 5778369 |
|  | MGCH | 17 | 10 | 16340012 | 16861707 |
|  | MGCH | 17 | 21 | 16938878 | 18493430 |
|  | MGCH | 17 | 10 | 44341275 | 44885924 |
|  | MGCH | 17 | 15 | 46085390 | 46832863 |
|  | MGCH | 18 | 28 | 20761621 | 22508135 |
|  | MGCH | 2 | 8 | 35552713 | 35845542 |
|  | MGCH | 2 | 10 | 41247854 | 41802155 |
|  | MGCH | 2 | 23 | 65616915 | 66622515 |
|  | MGCH | 2 | 6 | 67057322 | 67379951 |
|  | MGCH | 2 | 22 | 89102570 | 90215983 |
|  | MGCH | 2 | 11 | 104987617 | 105471694 |
|  | MGCH | 2 | 4 | 106796556 | 107088777 |
|  | MGCH | 2 | 6 | 107166216 | 107383516 |
|  | MGCH | 20 | 21 | 40443269 | 41484891 |
|  | MGCH | 21 | 24 | 43483911 | 44615173 |
|  | MGCH | 21 | 10 | 47969900 | 48367258 |
|  | MGCH | 22 | 17 | 935947 | 1644317 |
|  | MGCH | 22 | 5 | 1887707 | 2147767 |
|  | MGCH | 22 | 19 | 10191804 | 11126640 |
|  | MGCH | 22 | 34 | 46603601 | 48378372 |
|  | MGCH | 22 | 6 | 48721565 | 48964641 |
|  | MGCH | 24 | 22 | 20913363 | 22001230 |
|  | MGCH | 25 | 22 | 11669504 | 12819054 |
|  | MGCH | 26 | 5 | 6993164 | 7185824 |
|  | MGCH | 26 | 7 | 9589617 | 9942156 |
|  | MGCH | 27 | 41 | 8880484 | 11202412 |
|  | MGCH | 27 | 12 | 11274136 | 11970130 |
|  | MGCH | 27 | 25 | 21912525 | 23048138 |
|  | MGCH | 27 | 18 | 24183966 | 25011036 |
|  | MGCH | 3 | 7 | 95345743 | 95693458 |
|  | MGCH | 3 | 31 | 95941565 | 97290881 |
|  | MGCH | 4 | 5 | 112640532 | 112798683 |
|  | MGCH | 5 | 10 | 97567890 | 98183523 |
|  | MGCH | 6 | 28 | 85723685 | 86198530 |
|  | MGCH | 7 | 20 | 895002 | 1727858 |
|  | MGCH | 7 | 27 | 80625938 | 81884915 |
|  | MGCH | 8 | 22 | 63488196 | 64437081 |
|  | MGCH | 8 | 13 | 82051652 | 82657601 |
|  | MGCH | 8 | 7 | 82965079 | 83254950 |
|  | MGCH | 9 | 21 | 11386812 | 12549232 |
|  | MGCH | 9 | 16 | 12653205 | 13395631 |
|  | MGCH | 9 | 20 | 19290550 | 20332533 |
|  | MGCH | 9 | 25 | 32283141 | 33733022 |
|  | ZACH | 14 | 30 | 53089605 | 54743414 |
|  | ZACH | 18 | 11 | 15053358 | 16281915 |
|  | ZACH | 22 | 21 | 20724342 | 21756218 |
|  | ZACH | 22 | 9 | 22939647 | 23513867 |
|  | ZACH | 23 | 61 | 20496 | 3483182 |
|  | ZACH | 23 | 39 | 14767663 | 16985434 |
|  | ZACH | 5 | 30 | 87227704 | 88557676 |
|  | ZACH | 6 | 7 | 70559150 | 71044898 |

| Breed | Population | chr | Nº SNP | from | to |
| --- | --- | --- | --- | --- | --- |
| SAANEN | SAA_KECH | 1 | 5 | 102775573 | 102996619 |
|  | SAA_KECH | 11 | 20 | 69271831 | 70035327 |
|  | SAA_KECH | 12 | 1 | 51582111 | 51582111 |
|  | SAA_KECH | 13 | 19 | 46230843 | 46837001 |
|  | SAA_KECH | 17 | 20 | 8797513 | 9901508 |
|  | SAA_KECH | 18 | 16 | 25699693 | 26529141 |
|  | SAA_KECH | 18 | 8 | 40264679 | 40553632 |
|  | SAA_KECH | 27 | 21 | 12058932 | 13019344 |
|  | SAA_KECH | 4 | 18 | 4033243 | 4944453 |
|  | SAA_KECH | 6 | 20 | 19887362 | 20745509 |
|  | SAA_KECH | 6 | 24 | 81745130 | 83054831 |
|  | SAA_KECH | 7 | 6 | 55819424 | 56083993 |

| Breed | Population | chr | num_SNP | from | to |
| --- | --- | --- | --- | --- | --- |
|  | BOE_AUCH | 11 | 25 | 11153528 | 12646658 |
|  | BOE_AUCH | 12 | 23 | 43300149 | 45383753 |
|  | BOE_AUCH | 13 | 15 | 21997144 | 22938705 |
|  | BOE_AUCH | 13 | 220 | 23412290 | 35857527 |
|  | BOE_AUCH | 13 | 9 | 35951605 | 36667250 |
|  | BOE_AUCH | 14 | 21 | 22776618 | 23680789 |
|  | BOE_AUCH | 14 | 44 | 38509610 | 41301980 |
|  | BOE_AUCH | 14 | 22 | 49212683 | 50248470 |
|  | BOE_AUCH | 17 | 80 | 12377270 | 18118235 |
|  | BOE_AUCH | 19 | 32 | 53608888 | 55248736 |
|  | BOE_AUCH | 19 | 4 | 55772313 | 55961243 |
|  | BOE_AUCH | 2 | 9 | 17292393 | 17696165 |
|  | BOE_AUCH | 23 | 21 | 36534442 | 37415463 |
|  | BOE_AUCH | 24 | 6 | 53706924 | 54147886 |
|  | BOE_AUCH | 24 | 21 | 57775467 | 58848402 |
|  | BOE_AUCH | 24 | 11 | 59490376 | 60026051 |
|  | BOE_AUCH | 25 | 6 | 28893524 | 29077480 |
|  | BOE_AUCH | 25 | 21 | 29845418 | 30744322 |
|  | BOE_AUCH | 25 | 24 | 36282370 | 37531956 |
|  | BOE_AUCH | 27 | 49 | 3810325 | 6043316 |
|  | BOE_AUCH | 3 | 5 | 52834633 | 53043473 |
|  | BOE_AUCH | 3 | 3 | 56642564 | 56741635 |
|  | BOE_AUCH | 3 | 3 | 57017014 | 57129182 |
|  | BOE_AUCH | 3 | 136 | 88554321 | 95693458 |
|  | BOE_AUCH | 5 | 25 | 68923042 | 70424314 |
|  | BOE_AUCH | 6 | 91 | 6514342 | 11595263 |
|  | BOE_AUCH | 6 | 8 | 81745130 | 82219569 |
|  | BOE_AUCH | 6 | 35 | 85946764 | 86403944 |
|  | BOE_AUCH | 7 | 12 | 48694955 | 49445383 |
|  | BOE_AUCH | 7 | 15 | 50677764 | 51325562 |
|  | BOE_AUCH | 7 | 15 | 74866993 | 75678116 |
|  | BOE_AUCH | 7 | 29 | 76089778 | 78014662 |
|  | BOE_AUCH | 7 | 14 | 80010488 | 80810413 |
|  | BOE_AUCH | 7 | 48 | 81186029 | 83477829 |
|  | BOE_AUCH | 7 | 21 | 96144539 | 97269673 |
|  | BOE_AUCH | 7 | 13 | 99462861 | 100249346 |
|  | BOE_AUCH | 8 | 5 | 18934213 | 19101919 |
|  | BOE_AUCH | 8 | 29 | 33854745 | 35319779 |
|  | BOE_AUCH | 8 | 21 | 39965634 | 41353029 |
|  | BOE_AUCH | 8 | 6 | 107942465 | 108205765 |
|  | BOE_AUCH | 8 | 19 | 110147702 | 111311415 |
|  | BOE_AUCH | 9 | 26 | 47126719 | 48407483 |
|  | BOE_CHCH | 13 | 77 | 21071570 | 25730453 |
|  | BOE_CHCH | 14 | 22 | 33534857 | 34916722 |
|  | BOE_CHCH | 14 | 9 | 38693015 | 39300418 |
|  | BOE_CHCH | 15 | 10 | 12527848 | 12926995 |
|  | BOE_CHCH | 15 | 24 | 13263035 | 14568604 |
|  | BOE_CHCH | 18 | 20 | 12049364 | 13190280 |
|  | BOE_CHCH | 18 | 11 | 15053358 | 15835471 |
|  | BOE_CHCH | 22 | 7 | 34887539 | 35168655 |
|  | BOE_CHCH | 22 | 76 | 35345734 | 39281949 |
|  | BOE_CHCH | 22 | 17 | 40106834 | 40927637 |
|  | BOE_CHCH | 24 | 28 | 265939 | 1780694 |
|  | BOE_CHCH | 24 | 37 | 3489570 | 5288912 |
|  | BOE_CHCH | 24 | 20 | 8545841 | 9494284 |
|  | BOE_CHCH | 25 | 8 | 22172049 | 22460983 |
|  | BOE_CHCH | 26 | 21 | 26532923 | 27545518 |
|  | BOE_CHCH | 3 | 6 | 78856396 | 79026201 |
|  | BOE_CHCH | 3 | 6 | 83390302 | 83736997 |
|  | BOE_CHCH | 3 | 41 | 93751703 | 95693458 |
|  | BOE_CHCH | 6 | 9 | 4504717 | 4803684 |
|  | BOE_CHCH | 6 | 11 | 5147912 | 5747165 |
|  | BOE_CHCH | 6 | 27 | 6514342 | 8054263 |
|  | BOE_CHCH | 6 | 8 | 81745130 | 82219569 |
|  | BOE_CHCH | 6 | 40 | 85946764 | 86617703 |
|  | BOE_CHCH | 6 | 5 | 101022226 | 101250006 |
|  | BOE_CHCH | 7 | 8 | 51191300 | 51514889 |
|  | BOE_CHCH | 7 | 14 | 78986494 | 79817948 |
|  | BOE_CHCH | 7 | 6 | 80073300 | 80351081 |
|  | BOE_CHCH | 8 | 45 | 34228108 | 36792443 |
|  | BOE_CHCH | 8 | 5 | 97255523 | 97607087 |
|  | BOE_NZCH | 1 | 27 | 131528543 | 133142464 |
|  | BOE_NZCH | 1 | 26 | 134581196 | 135918715 |
|  | BOE_NZCH | 10 | 18 | 87139204 | 88132129 |
|  | BOE_NZCH | 11 | 13 | 31869585 | 32457125 |
|  | BOE_NZCH | 11 | 27 | 32929858 | 34326607 |
|  | BOE_NZCH | 12 | 11 | 38148942 | 38609238 |
|  | BOE_NZCH | 12 | 46 | 43300149 | 46561846 |
|  | BOE_NZCH | 12 | 7 | 72945325 | 73214084 |
|  | BOE_NZCH | 13 | 30 | 16550892 | 18284539 |
|  | BOE_NZCH | 13 | 78 | 21071570 | 25797173 |
|  | BOE_NZCH | 13 | 22 | 27099672 | 28174550 |
|  | BOE_NZCH | 13 | 10 | 28935639 | 29423970 |
|  | BOE_NZCH | 13 | 32 | 31918186 | 33584903 |
|  | BOE_NZCH | 14 | 7 | 16226731 | 16622714 |
|  | BOE_NZCH | 14 | 40 | 49923878 | 52357199 |
|  | BOE_NZCH | 14 | 27 | 52503124 | 53628574 |
|  | BOE_NZCH | 14 | 8 | 74682609 | 75048615 |
|  | BOE_NZCH | 14 | 15 | 81146552 | 82092005 |
|  | BOE_NZCH | 15 | 19 | 75127718 | 76306241 |
|  | BOE_NZCH | 16 | 7 | 33868526 | 34152026 |
|  | BOE_NZCH | 16 | 5 | 34712765 | 34949375 |
|  | BOE_NZCH | 17 | 15 | 12092483 | 12826921 |
|  | BOE_NZCH | 17 | 112 | 13034038 | 20419879 |
|  | BOE_NZCH | 2 | 8 | 73544201 | 73868842 |
|  | BOE_NZCH | 2 | 19 | 90182903 | 91037521 |
|  | BOE_NZCH | 20 | 14 | 1385746 | 2115121 |
|  | BOE_NZCH | 20 | 42 | 48694352 | 51127003 |
|  | BOE_NZCH | 20 | 8 | 57338711 | 57625205 |
|  | BOE_NZCH | 20 | 8 | 58152055 | 58559998 |
|  | BOE_NZCH | 20 | 31 | 59906994 | 61535050 |
|  | BOE_NZCH | 22 | 118 | 33085641 | 39281949 |
|  | BOE_NZCH | 24 | 37 | 1494988 | 3229772 |
|  | BOE_NZCH | 25 | 12 | 7569990 | 8078853 |
|  | BOE_NZCH | 27 | 43 | 3923321 | 5848746 |
|  | BOE_NZCH | 27 | 26 | 10304461 | 12114700 |
|  | BOE_NZCH | 27 | 5 | 12453012 | 12685511 |
|  | BOE_NZCH | 27 | 11 | 17568202 | 18555737 |
|  | BOE_NZCH | 28 | 13 | 1368692 | 1906063 |
|  | BOE_NZCH | 29 | 21 | 44576267 | 45709872 |
|  | BOE_NZCH | 29 | 17 | 46223281 | 47226986 |
|  | BOE_NZCH | 3 | 15 | 79875903 | 80675857 |
|  | BOE_NZCH | 3 | 22 | 85856559 | 87190283 |
|  | BOE_NZCH | 3 | 71 | 89364966 | 93346132 |
|  | BOE_NZCH | 3 | 29 | 93751703 | 95177895 |
|  | BOE_NZCH | 4 | 11 | 43595687 | 44130443 |
|  | BOE_NZCH | 4 | 14 | 91552063 | 92092771 |
|  | BOE_NZCH | 6 | 6 | 1756523 | 2047262 |
|  | BOE_NZCH | 6 | 44 | 5147912 | 8054263 |
|  | BOE_NZCH | 6 | 11 | 14254732 | 14732934 |
|  | BOE_NZCH | 6 | 16 | 15501584 | 16301985 |
|  | BOE_NZCH | 6 | 40 | 26964833 | 29025606 |
|  | BOE_NZCH | 6 | 13 | 36360840 | 37023063 |
|  | BOE_NZCH | 6 | 27 | 80618555 | 82219569 |
|  | BOE_NZCH | 6 | 35 | 85946764 | 86403944 |
|  | BOE_NZCH | 7 | 12 | 99570958 | 100303196 |
|  | BOE_NZCH | 8 | 29 | 18556391 | 19855553 |
|  | BOE_NZCH | 9 | 20 | 23077830 | 23996428 |
|  | BOE_NZCH | 9 | 27 | 75523935 | 77039202 |
|  | BOE_TZCH | 1 | 77 | 48085411 | 52553074 |
|  | BOE_TZCH | 1 | 13 | 54901685 | 55474455 |
|  | BOE_TZCH | 1 | 31 | 56299249 | 57786867 |
|  | BOE_TZCH | 1 | 10 | 59409177 | 59794887 |
|  | BOE_TZCH | 12 | 19 | 50253456 | 51252291 |
|  | BOE_TZCH | 15 | 20 | 71029579 | 72124753 |
|  | BOE_TZCH | 15 | 31 | 73074723 | 74918028 |
|  | BOE_TZCH | 17 | 21 | 10729752 | 11791586 |
|  | BOE_TZCH | 17 | 28 | 12377270 | 13849020 |
|  | BOE_TZCH | 17 | 29 | 18847537 | 20389997 |
|  | BOE_TZCH | 17 | 28 | 21549906 | 22921164 |
|  | BOE_TZCH | 17 | 28 | 38183606 | 39473881 |
|  | BOE_TZCH | 17 | 8 | 67631894 | 67902841 |
|  | BOE_TZCH | 2 | 36 | 3381613 | 5179270 |
|  | BOE_TZCH | 2 | 22 | 33337846 | 34668549 |
|  | BOE_TZCH | 2 | 68 | 51165564 | 55850416 |
|  | BOE_TZCH | 2 | 25 | 78214820 | 79767454 |
|  | BOE_TZCH | 20 | 27 | 31734968 | 33064194 |
|  | BOE_TZCH | 28 | 29 | 1011821 | 2212194 |
|  | BOE_TZCH | 28 | 30 | 38533498 | 39829470 |
|  | BOE_TZCH | 3 | 94 | 85359533 | 90313098 |
|  | BOE_TZCH | 4 | 25 | 114179121 | 115826699 |
|  | BOE_TZCH | 6 | 36 | 513324 | 2347436 |
|  | BOE_TZCH | 6 | 73 | 3615807 | 7672687 |
|  | BOE_TZCH | 6 | 53 | 26964833 | 29815950 |
|  | BOE_TZCH | 6 | 19 | 31851817 | 32996417 |
|  | BOE_TZCH | 6 | 7 | 45042013 | 45331924 |
|  | BOE_TZCH | 6 | 11 | 81606037 | 82237885 |
|  | BOE_TZCH | 6 | 35 | 85946764 | 86403944 |
|  | BOE_TZCH | 7 | 24 | 64948126 | 66172784 |
|  | BOE_TZCH | 7 | 20 | 85312880 | 86261716 |
|  | BOE_TZCH | 8 | 19 | 26058187 | 26906241 |
|  | BOE_TZCH | 8 | 131 | 36601889 | 44046942 |
|  | BOE_UGCH | 1 | 22 | 134543743 | 135732399 |
|  | BOE_UGCH | 1 | 19 | 142505324 | 143324182 |
|  | BOE_UGCH | 11 | 34 | 33447956 | 35288701 |
|  | BOE_UGCH | 11 | 20 | 37591807 | 38857210 |
|  | BOE_UGCH | 11 | 20 | 69304960 | 70073534 |
|  | BOE_UGCH | 11 | 21 | 97849731 | 99151641 |
|  | BOE_UGCH | 12 | 5 | 23149388 | 23415206 |
|  | BOE_UGCH | 12 | 10 | 56270967 | 56639881 |
|  | BOE_UGCH | 14 | 23 | 60132791 | 61336381 |
|  | BOE_UGCH | 15 | 29 | 29087396 | 30900595 |
|  | BOE_UGCH | 15 | 6 | 52271614 | 52449476 |
|  | BOE_UGCH | 2 | 20 | 15252241 | 16103527 |
|  | BOE_UGCH | 2 | 40 | 21774549 | 23627967 |
|  | BOE_UGCH | 2 | 15 | 56544211 | 57382166 |
|  | BOE_UGCH | 20 | 11 | 50308512 | 50736582 |
|  | BOE_UGCH | 20 | 26 | 56740926 | 57954150 |
|  | BOE_UGCH | 21 | 22 | 3923319 | 5012984 |
|  | BOE_UGCH | 25 | 102 | 26590525 | 31632295 |
|  | BOE_UGCH | 25 | 23 | 36251456 | 37462519 |
|  | BOE_UGCH | 27 | 26 | 16945841 | 18603239 |
|  | BOE_UGCH | 3 | 49 | 43423648 | 45838988 |
|  | BOE_UGCH | 3 | 28 | 77743009 | 79026201 |
|  | BOE_UGCH | 3 | 20 | 87341066 | 88174479 |
|  | BOE_UGCH | 3 | 25 | 116061501 | 117570387 |
|  | BOE_UGCH | 4 | 21 | 100962814 | 102093391 |
|  | BOE_UGCH | 4 | 15 | 103059400 | 103782098 |
|  | BOE_UGCH | 6 | 23 | 56613395 | 57827231 |
|  | BOE_UGCH | 6 | 39 | 85913548 | 86530449 |
|  | BOE_UGCH | 6 | 12 | 99977370 | 100727119 |
|  | BOE_UGCH | 6 | 25 | 109456269 | 110470931 |
|  | BOE_UGCH | 7 | 22 | 66054823 | 67642242 |
|  | BOE_UGCH | 7 | 14 | 72493234 | 73122843 |
|  | BOE_USCH | 11 | 15 | 79626957 | 80263413 |
|  | BOE_USCH | 12 | 46 | 43300149 | 46561846 |
|  | BOE_USCH | 13 | 7 | 16550892 | 17104519 |
|  | BOE_USCH | 13 | 20 | 30808794 | 31966423 |
|  | BOE_USCH | 13 | 3 | 32226075 | 32300817 |
|  | BOE_USCH | 17 | 2 | 12377270 | 12426219 |
|  | BOE_USCH | 17 | 6 | 13688601 | 14201587 |
|  | BOE_USCH | 17 | 12 | 14409170 | 15112473 |
|  | BOE_USCH | 17 | 5 | 16413479 | 16717396 |
|  | BOE_USCH | 17 | 31 | 38994171 | 40430958 |
|  | BOE_USCH | 18 | 14 | 12150483 | 12788768 |
|  | BOE_USCH | 20 | 9 | 43050436 | 43798601 |
|  | BOE_USCH | 22 | 25 | 25724214 | 27190249 |
|  | BOE_USCH | 25 | 17 | 22289506 | 22996833 |
|  | BOE_USCH | 25 | 64 | 24397156 | 27595116 |
|  | BOE_USCH | 25 | 28 | 29845418 | 31057461 |
|  | BOE_USCH | 25 | 12 | 33157943 | 33819072 |
|  | BOE_USCH | 27 | 17 | 5056119 | 5848746 |
|  | BOE_USCH | 29 | 19 | 44653728 | 45709872 |
|  | BOE_USCH | 29 | 6 | 46223281 | 46608534 |
|  | BOE_USCH | 3 | 5 | 79496338 | 79769740 |
|  | BOE_USCH | 3 | 272 | 79875903 | 94536756 |
|  | BOE_USCH | 3 | 20 | 94855248 | 95693458 |
|  | BOE_USCH | 6 | 32 | 11828141 | 13195756 |
|  | BOE_USCH | 6 | 24 | 13301964 | 14613402 |
|  | BOE_USCH | 6 | 9 | 80618555 | 81082930 |
|  | BOE_USCH | 6 | 16 | 81234384 | 82219569 |
|  | BOE_USCH | 7 | 37 | 54734578 | 56595929 |
|  | BOE_USCH | 8 | 30 | 35016727 | 36792443 |
|  | BOE_USCH | 8 | 24 | 88800059 | 89973519 |
|  | BOE_ZWCH | 1 | 21 | 56552803 | 57597486 |
|  | BOE_ZWCH | 1 | 31 | 62835455 | 64625165 |
|  | BOE_ZWCH | 1 | 17 | 82425110 | 83493210 |
|  | BOE_ZWCH | 1 | 31 | 99914986 | 101459994 |
|  | BOE_ZWCH | 11 | 20 | 33775384 | 34829666 |
|  | BOE_ZWCH | 11 | 14 | 98011738 | 98861512 |
|  | BOE_ZWCH | 13 | 9 | 38182963 | 38592849 |
|  | BOE_ZWCH | 13 | 27 | 38981915 | 40342302 |
|  | BOE_ZWCH | 18 | 20 | 12091788 | 13225681 |
|  | BOE_ZWCH | 18 | 18 | 21081558 | 21894816 |
|  | BOE_ZWCH | 20 | 9 | 43050436 | 43798601 |
|  | BOE_ZWCH | 20 | 47 | 44063000 | 47095219 |
|  | BOE_ZWCH | 20 | 28 | 47576667 | 49220814 |
|  | BOE_ZWCH | 20 | 9 | 49335596 | 49956593 |
|  | BOE_ZWCH | 20 | 10 | 50702246 | 51127003 |
|  | BOE_ZWCH | 20 | 42 | 65604840 | 67583226 |
|  | BOE_ZWCH | 28 | 7 | 27588531 | 27893879 |
|  | BOE_ZWCH | 3 | 10 | 70859283 | 71335899 |
|  | BOE_ZWCH | 5 | 29 | 53267677 | 54898557 |
|  | BOE_ZWCH | 5 | 9 | 104354519 | 104808889 |
|  | BOE_ZWCH | 6 | 35 | 85946764 | 86403944 |
|  | BOE_ZWCH | 7 | 9 | 59028681 | 59498016 |
|  | BOE_ZWCH | 8 | 9 | 34846886 | 35319779 |
|  | BOE_ZWCH | 8 | 16 | 99030950 | 100070733 |
|  | BOE_ZWCH | 8 | 9 | 109457167 | 109743438 |
|  | BOE_ZWCH | 9 | 10 | 20989344 | 21537186 |
|  | BOE_ZWCH | 9 | 8 | 47920174 | 48407483 |
